# Merging Information From Infrared and Autofluorescence Fundus Images for Monitoring of Chorioretinal Atrophic Lesions

**DOI:** 10.1167/tvst.9.9.38

**Published:** 2020-08-25

**Authors:** Giovanni Ometto, Giovanni Montesano, Saman Sadeghi Afgeh, Georgios Lazaridis, Xiaoxuan Liu, Pearse A. Keane, David P. Crabb, Alastair K. Denniston

**Affiliations:** 1 Division of Optometry and Visual Sciences, School of Health Sciences, City, University of London, London, UK; 2 Moorfields Eye Hospital NHS Foundation Trust, London, UK; 3 Data Science Institute, City, University of London, London, UK; 4 Centre for Medical Image Computing, University College London, London, UK; 5 Department of Ophthalmology, Queen Elizabeth Hospital Birmingham, University Hospitals Birmingham NHS Foundation Trust, Birmingham, UK; 6 Academic Unit of Ophthalmology, Institute of Inflammation & Ageing, College of Medical and Dental Sciences, University of Birmingham, Edgbaston, Birmingham, UK; 7 Health Data Research UK, London, UK; 8 NIHR Biomedical Research Centre at Moorfields Eye Hospital NHS Foundation Trust and UCL Institute of Ophthalmology, UK

**Keywords:** segmentation, multimodal, autofluorescence, infrared, uveitis

## Abstract

**Purpose:**

To develop a method for automated detection and progression analysis of chorioretinal atrophic lesions using the combined information of standard infrared (IR) and autofluorescence (AF) fundus images.

**Methods:**

Eighteen eyes (from 16 subjects) with punctate inner choroidopathy were analyzed. Macular IR and blue AF images were acquired in all eyes with a Spectralis HRA+OCT device (Heidelberg Engineering, Heidelberg, Germany). Two clinical experts manually segmented chorioretinal lesions on the AF image. AF images were aligned to the corresponding IR. Two random forest models were trained to classify pixels of lesions, one based on the AF image only, the other based on the aligned IR-AF. The models were validated using a leave-one-out cross-validation and were tested against the manual segmentation to compare their performance. A time series from one eye was identified and used to evaluate the method based on the IR-AF in a case study.

**Results:**

The method based on the AF images correctly classified 95% of the pixels (i.e., in vs. out of the lesion) with a Dice's coefficient of 0.80. The method based on the combined IR-AF correctly classified 96% of the pixels with a Dice's coefficient of 0.84.

**Conclusions:**

The automated segmentation of chorioretinal lesions using IR and AF shows closer alignment to manual segmentation than the same method based on AF only. Merging information from multimodal images improves the automatic and objective segmentation of chorioretinal lesions even when based on a small dataset.

**Translational Relevance:**

Merged information from multimodal images improves segmentation performance of chorioretinal lesions.

## Introduction

Punctuate inner choroidopathy (PIC) is a rare condition that was first recognized by Watzke in 1984 as a group of the White Dot Syndromes.^1^ The disease is an inflammatory choroiditis that does not affect the anterior chamber or vitreous cavity. It generally affects eyes of young and myopic women and its cause is unknown.^2^ It is characterized by the appearance of multifocal, well-circumscribed, small lesions that resolve in a few weeks, leaving atrophic spots with variable pigmentation. These episodes are symptomatic, with patients reporting blurred central vision, flashes of light, and paracentral scotomas. Symptoms can disappear with the resolution of the lesion, but about 40% of the patients experience more severe visual loss with the development of choroidal neovascularization (CNV).^3^^–^^5^

The detection and monitoring of PIC are assisted by a number of imaging techniques including optical coherence tomography (OCT) and fundus autofluorescence (AF).^6^^,^^7^ Whereas OCT provides good three-dimensional views of the evolution of individual inflammatory lesions (and detection of CNV), the AF provides the best overview of the number of atrophic PIC lesions, their size and the total area of the macula that has been affected. Specialists therefore routinely use the evaluation of hypoautofluorescent areas on AF images to estimate disease progression over time and to assess efficacy of treatment; additionally hyperautofluorescent areas may indicate new disease activity although this is a less consistent phenomenon.^8^ Visual assessment is, however, significantly hampered by its subjective nature (based on direct visual comparison of scans between visits) and is not supported by any numerical information that could be used to provide objective indices to support treatment decisions or progression monitoring. Estimates of lesions in AF can be improved by semiautomatic segmentation, such as the one provided by the Region Finder software (Heidelberg Engineering, Heidelberg, Germany), a region-based segmentation that often requires manual correction.^9^ There is also considerable interest around the automated segmentation of geographic atrophic lesions in patients with age-related macular degeneration.^10^^–^^19^ However, to the best of our knowledge, there are no published algorithms for the automated segmentation of chorioretinal lesions in PIC and similar uveitic syndromes, and clinicians are therefore currently dependent on subjective, qualitative assessment to detect change between visits and inform treatment decisions. This is likely due to the scarcity of large datasets, rarity of the disease and the morphological complexity of these lesions that make the training of such algorithms a challenging task. Such complexity would also translate into a taxing endeavor for clinicians, required to manually correct segmentations of multiple sparse lesions.

Chorioretinal atrophic lesions are also visible in images acquired in infrared (IR) and color modalities. In these images, lesions appear as sharply demarcated regions of absent retinal pigment epithelium through which the choroid or sclera is visible. Although their appearance in these images might not be as contrasted as in AF, they provide complementary information that can improve the performance of algorithms for automatic segmentation.

In this work, we present a proof of concept for a machine learning algorithm, which combines the information of IR and AF images to produce an automatic segmentation of PIC atrophic lesions. We demonstrate that it is feasible to develop and test these methods using a small dataset. We compare the results of the proposed method with those from another algorithm, based on the same model but trained on the AF only. Finally, we present a case study to explore the potential benefits of the technique for the monitoring of progression.

## Methods

### Dataset

All patients attending the specialist PIC clinic at University Hospitals Birmingham NHS Foundation Trust, UK, have a standardized set of scans (the “Birmingham PIC Protocol”) which comprises: 30° OCT of macula (“fast macula”) and retinal nerve fiber layer (RNFL); 30° Bluepeak AutoFluorescence (AF); 30° multicolor (three laser reflectance), 55° wide-field posterior pole OCT, AF, and Multicolor, Ultrawide field Optos Color, and AF. This protocol was approved from the NRES East Midlands Ethics Committee (Ref: 14/EM/1163). Written informed consent was gathered from all subjects. This protocol adhered to the tenets of the declaration of Helsinki. The scans of 16 patients (with 18 affected eyes) with PIC had at least one visit with all modalities acquired and were identified for this study. Each of the 18 eyes had a 6 mm × 6 mm macular OCT volume with an associated 768 × 768-pixels IR image and a 768 × 768-pixels AF image. All volumes and AF images were acquired using a Spectralis HRA+OCT device (Heidelberg Engineering, Heidelberg, Germany) using 820 nm and 488 nm wavelengths for IR and AF, respectively. All patients enrolled in this study had “classic” PIC with predominantly central lesions; patients who had multifocal choroiditis without the central lesions or who had progressive subretinal fibrosis were not included in this study. All stages of PIC were eligible for inclusion.

### Manual Segmentation

**Figure 1.**
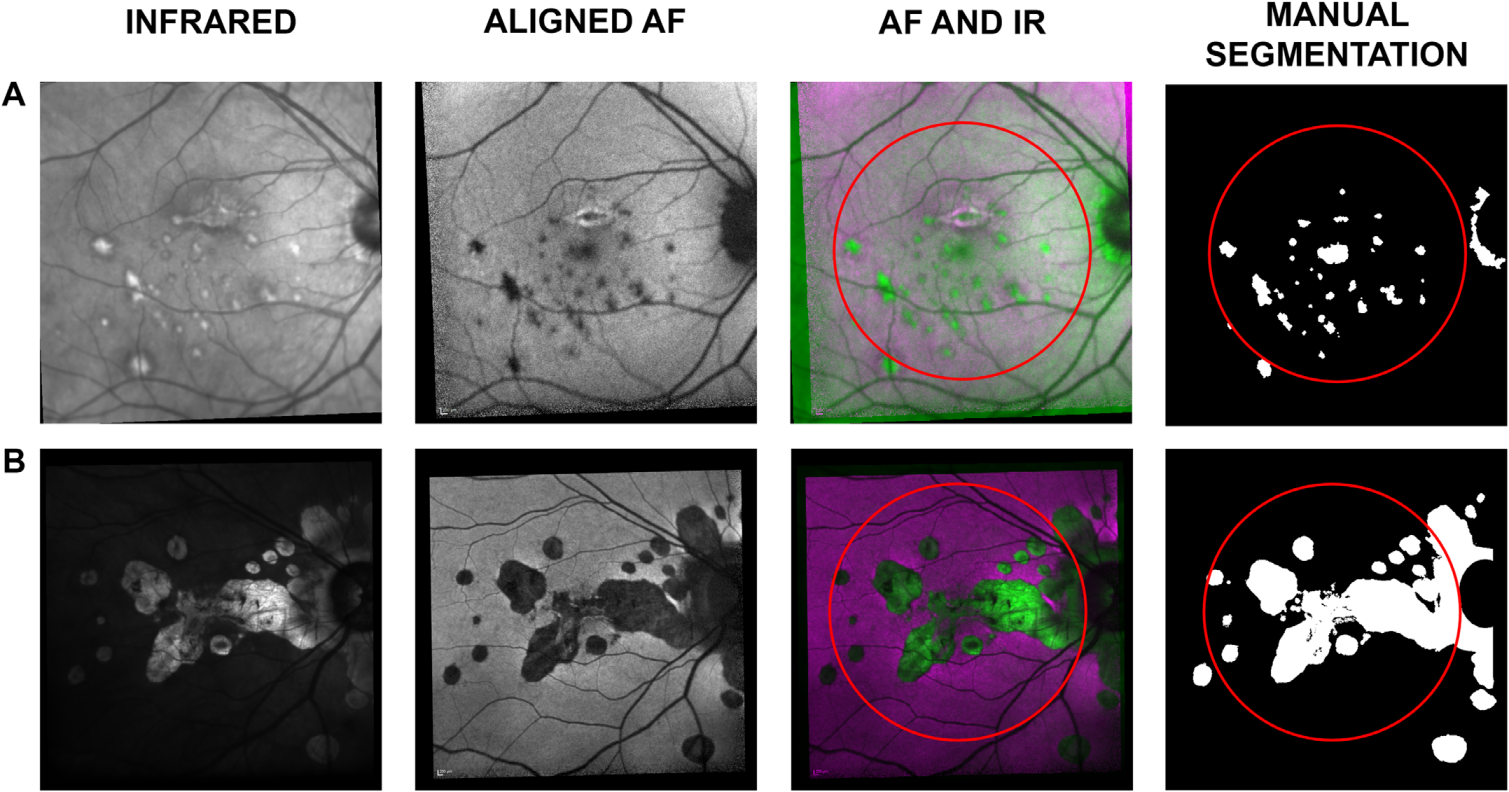
Rows A and B illustrate data from two of the 18 selected eyes. The first column shows the IR image, acquired with the macular OCT scan. The second column shows the aligned AF image. The third column shows a combination of the IR and AF, where the *magenta* represents intensities in the AF higher than in the IR and vice versa in *green*. The fourth column shows the manual segmentation as a binary map of “0” (non-lesion, in *black*) and “1” (lesion, in *white*). The *red circles* in the third and fourth columns show the central 22.5°, the area used for training and testing the automatic classification.

A clinical expert (GM) manually segmented the pixels of chorioretinal lesions on the AF image based on the appearance of the AF image supported by the IR and OCT. The segmentation was then reviewed by a second clinical expert (XL). The task was carried out using the ImageSegmenter app available in Matlab R2019a (Mathworks, Natick, MA, USA) with the Image Processing Toolbox. The segmentation produced eighteen 768 × 768 binary maps, classifying each pixel of the IR image as “0” (nonlesion) or “1” (lesion) (Fig. 1). Areas of peripapillary atrophy were also classified as lesions. This choice was necessary to avoid ambiguities in the segmentation of lesions that merged with these areas (see Fig. 1B).

### AF-IR Image Alignment

Matlab image registration function *imregconfig* was used to automatically align normalized AF images to the relative, normalized IR using a 100-step optimization process. The function was set to align mono-modal images, as the information captured by these two modalities was largely non-complementary, for the exception of areas with lesions. The clinical grader (GM) visually inspected the results of the alignment and performed manual matching where the automated algorithm failed. Manual alignment was obtained through the localization of four landmarks on the pair of images. The landmarks were used to calculate the parameters of the local weighted mean transformation, which was then used to align the AF. Control points and transformation parameters were obtained using the Image Processing Toolbox functions *cpselect* and *fitgeotrans*, respectively.

### Automatic Segmentation Methods

A machine learning classifier (random forest with 25 trees) was trained to categorize pixels of the images into two classes, “0” (non-lesion) or “1” (lesion), using the manual segmentation as the reference.^20^

Each observation was identified by a pixel location of the IR and aligned AF and was characterized by eight attributes. The latter were obtained with an adaptive histogram equalization of both IR and AF images operated on 4 differently sized, neighboring regions: 15 × 15, 31 × 31, 151 × 151 and 301 × 301 pixels. This process, equivalent to a pre-processing stage of local-intensity normalization, generated eight equalized images, four from IR and four from AF. Therefore, at each pixel location, these images provided an attribute that incorporated information of neighboring intensities.

Only pixels within the central 22.5° (3/4 of the field of view of the lens) were used in the validation of the model (red circles in Fig. 1). This restriction was introduced to exclude peripheral areas, often noisy due to scarce illumination, and to guarantee that the included macular area had been captured by both IR and AF despite acquisition misalignments.

Finally, a proportion (10%) of randomly selected observations from the “lesion” class was selected and matched by the same number of randomly selected “non-lesions.” This produced balanced training-datasets while allowing faster training.

The same random forest used for the classification of AF-IR was trained using the four attributes from the AF only, obtaining a new classification model.

### Validation

The random forest classifiers were evaluated using a leave-one-out cross-validation: the model was trained on observations from 17 of the 18 eyes, and was validated on the eye ‘left-out’, repeating the process for each eye.^21^ Results were analyzed to report the percentage of correct classifications, sensitivity and specificity of the model, as well as Dice's coefficient of similarity with the segmentation by the grader.^22^

### Case Study

A time series of IR and associated AF images acquired over 42 months from 35 consecutive visits was identified in the database and used for a case study. The 34 IR from follow-up visits were aligned to the IR of the first visit. Then, all AF images were aligned to their associated IR. Resulting pairs of IR-AF were classified by the trained random forest classifier. The generated classification-maps were processed to fill the holes in the segmentation; to remove spurious classifications of individual pixels as lesions with a morphological opening and closing; and to force pixels classified as lesion at a time point to retain the classification for the rest of the time series. Using the pixel-mm^2^ conversion provided by the manufacturer (1 pixel = 0.01118 mm^2^), the total segmented area at each visit was converted to millimeters squared. The first derivative of the total area was calculated to estimate the expansion speed in mm^2^/days.

## Results

The 16 patients selected for the study were all female with a mean (range) age of 41 (31, 62) years. All but one patient had both eyes affected by the condition and twelve patients were on systemic immunosuppression.

Automatic alignment was successful in nine out of the 18 AF-IR pairs, with the others requiring manual intervention. Failed alignments were associated with the presence of large lesions or large areas with low illumination in at least one of the two photographs (IR or AF).

**Figure 2.**
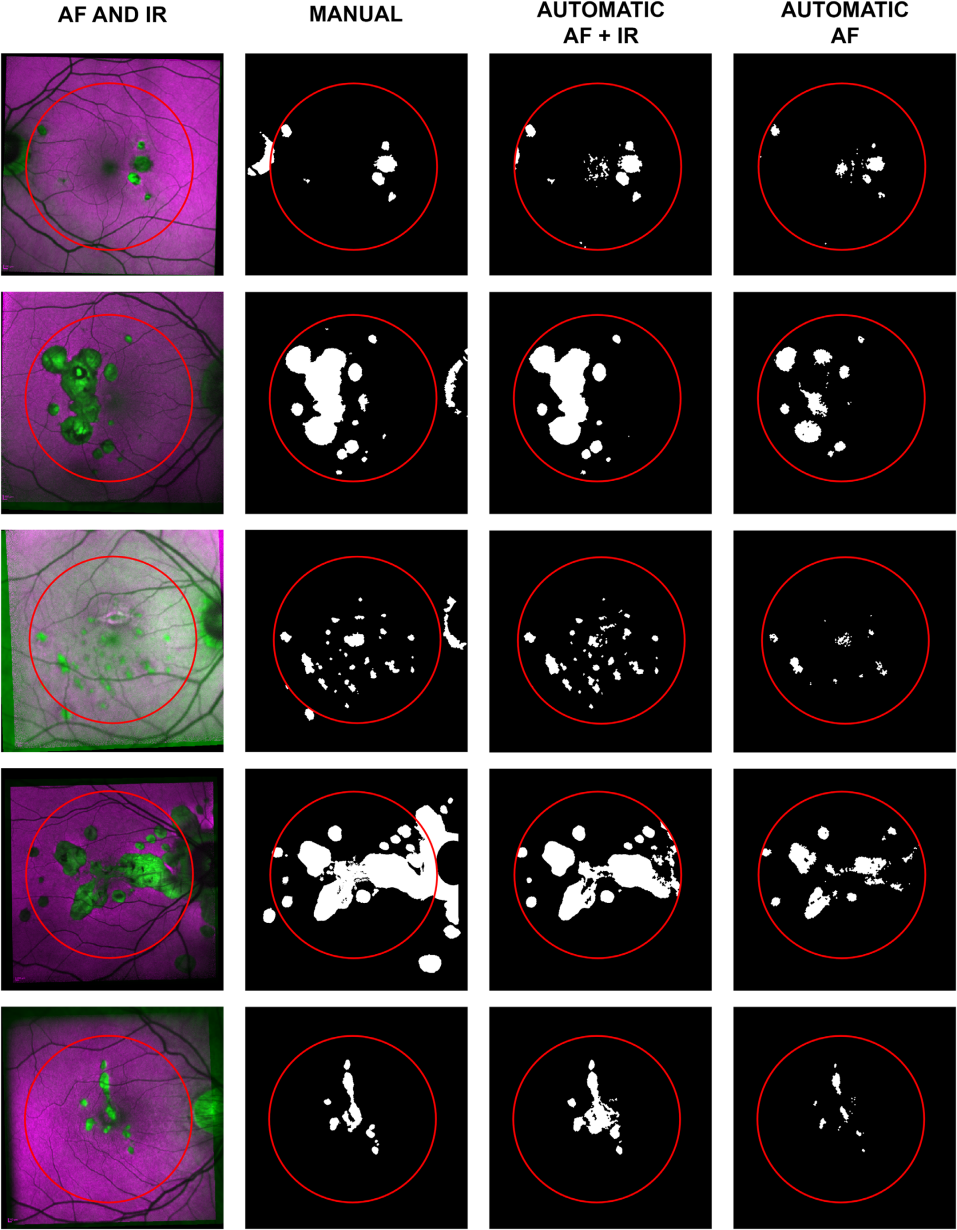
A random subset of five of the 18 selected eyes. The first column shows a combination of the IR and AF. The second column shows the manual segmentation as a binary map of “0” (non-lesion, in *black*) and “1” (lesion, in *white*). The third column shows the automatic segmentation based on IR and AF for the central 22.5°, delimited by a *red circle*. The fourth column shows the results of the same classification model trained on AF only.

The AF-IR model correctly classified 95.9% of the pixels in the dataset with sensitivity and specificity of 0.83 and 0.98 respectively. Dice's coefficient was 0.85, showing a good similarity between the automatic and manual segmentations. The AF model correctly classified 94.6% of the pixels in the dataset with sensitivity and specificity of 0.79 and 0.97, respectively; Dice's coefficient was 0.80. For reference, we trained the same model on IR only. The IR model correctly classified 90.0% of the pixels; sensitivity and specificity were 0.40 and 0.98 respectively; Dice's coefficient was 0.53 showing poorer correlation with the reference segmentation than the other models. Figure 2 shows the results of the automatic segmentation for a randomly selected subset of the dataset. Segmentation results of the whole dataset are available in the Supplementary Figure.

## Discussion

This work introduces a novel method for the segmentation of atrophic chorioretinal lesion. We demonstrate how this method could be feasibly used to provide clinicians with real-time objective metrics such as lesion area and growth rate.

PIC was used as a case-example of the wider group of chorioretinal inflammatory diseases (posterior uveitis) because there is a clear “use case” here in that the presence or absence of lesions, and their change over time, directly impacts on treatment decisions.

Our approach to chorioretinal lesion segmentation used a combination of two standard scanning laser ophthalmoscopy (SLO) imaging techniques—IR and AF—both of which can be routinely acquired from the Heidelberg Spectralis system. Our automated segmentation technique shows strong agreement with manual segmentation by a clinical grader while using only 18 images for the training of the algorithm.

The model based on the IR only performed poorly compared to the other two (AF only and IR-AF) and this result is consistent with previous literature.^8^^,^^9^ This report demonstrates new knowledge because merging the information from multimodal images proved to be effective, outperforming the classification model based on AF only. Percentage of correctly classified pixels and specificity do not highlight major differences in performance due to the much higher number of pixels from non-lesions. However, higher sensitivity and higher Dice's coefficient achieved by the IR-AF based model reflects a significant improvement in the segmentation, also clearly visible inspecting the segmentation results (Fig. 2 and Supplementary Figure). In fact, a challenge in lesion segmentation with traditional single modality techniques, is delineating between a pathological feature and a normal structure such as fovea, optic disc, and retinal vessels that could be wrongly classified as lesions. This problem requires particular attention and can be time-consuming when the task is performed semiautomatically with the aid of the Heidelberg Engineering Region Finder software.^23^ In this task, our proposed method outperforms the same algorithm trained on AF only, in part because the combination of IR and AF reinforces features of normal structures and differentiation of abnormalities: retinal features like the fovea and vessels (all darker in AF images and possibly confused with atrophic lesions) can therefore be ignored, and pathological features can be highlighted. In particular, better identification of lesions in the foveal region is of extreme importance due to their sight threatening implications. The better performance of the model based on multimodal images can be explained in part by its ability to exploit the most informative features provided by each acquisition modality, such as the generally sharper features of IR images and the intensities of lesions represented in AF images.

**Figure 3.**
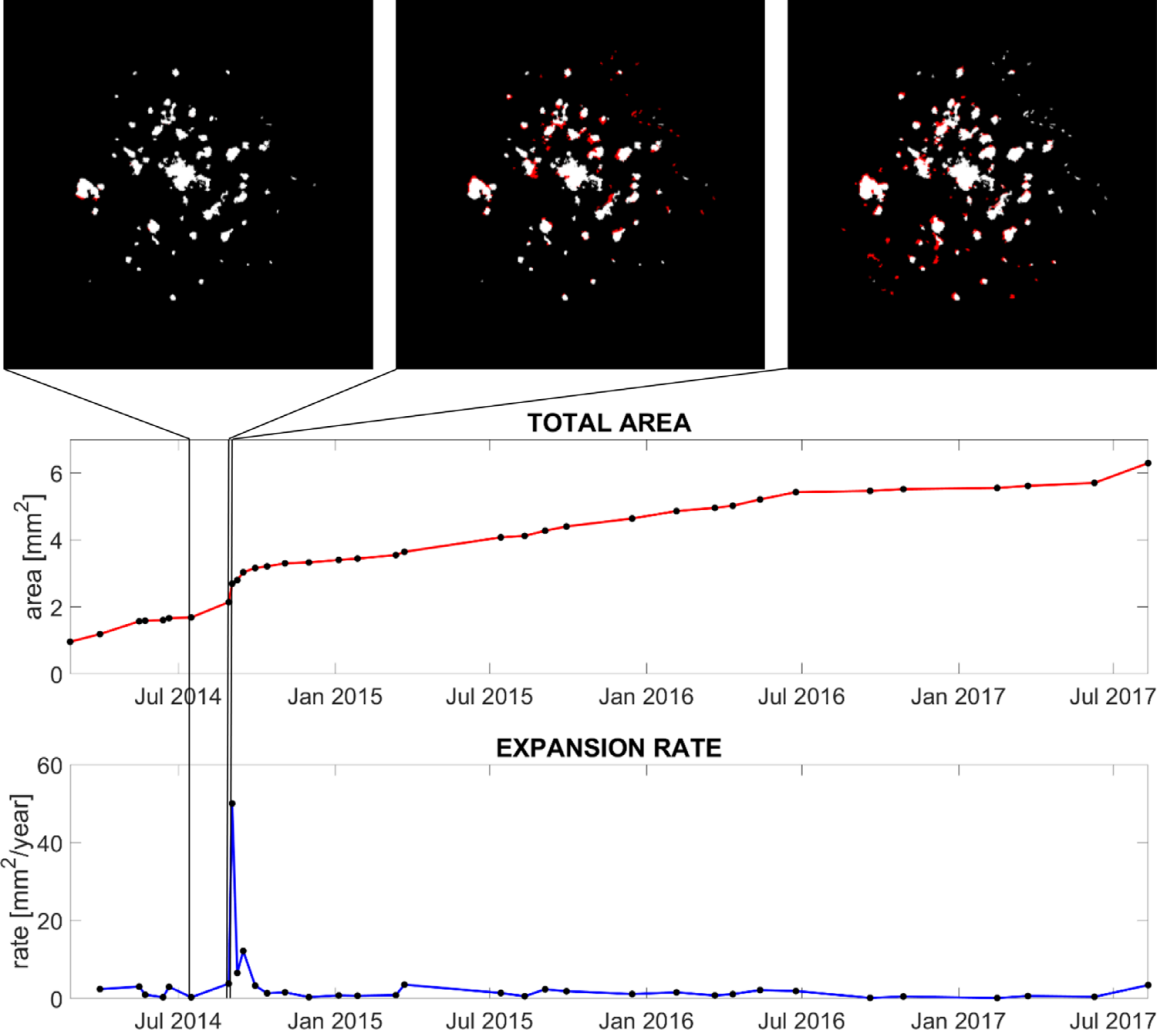
Case study of chorioretinal lesions development in the left eye of a young woman with PIC. The *red line* shows the total area of PIC atrophic lesions measured from the segmentation of the time series. The *blue line* shows the first derivative of the total area, or expansion rate. The three images on top of the plots show the segmentation of three acquisitions taken just before the peak in the expansion rate. *Black* represents nonlesions; *white* represents lesions already segmented in the previous visit; *red* highlights newly segmented lesions.

Although the proposed method still requires some modest manual intervention for the alignment, this allows for the tracking of areas that become atrophic during follow-ups. Thanks to the alignment, newly developed atrophic areas can be directly identified and highlighted for each visit (Fig. 3). This tracking not only increases the level of detail in the monitoring of the condition but can also represent an important quantification tool for outcomes for prospective research studies.

We have previously reported the use of commercial OCT segmentation software (HEYEX; Heidelberg Engineering) to identify new inflammatory PIC lesions on the OCT volume scans, using the heat-map function including the ability to generate heat-maps of change from a baseline scan.^7^ We see these techniques as complementary, since they provide information about different aspects of the disease process at different stages in the pathway. The heat-map technique (and indeed direct careful perusal of the volume scans) will identify inflammatory PIC lesions (and PIC-associated CNV) from a very early stage. At these early stages, the lesions may not be detected by our IR-AF technique if they have not caused sufficient disruption to the RPE to be seen as a hypoautofluorescent signal. The IR-AF technique detects these lesions slightly later in their development i.e. once they have caused loss of the RPE. Yet the technique segments the lesions themselves rather than the retinal layers and this represents a significant advantage. In contrast, the heat-map technique is primarily qualitative as it does not provide a direct measurement of individual lesions, total lesion area or change in lesion burden.

Although, to the best of our knowledge, ours is the first work to combine IR and AF images for semantic segmentation of PIC, some have used AF images for semantic segmentation of Geographic Atrophy (GA). These contributions can be roughly divided into three categories. First, region-based approaches, level-set methods and other computer vision techniques;^10^^,^^15^^,^^16^ second, heuristic methods and handcrafted features that are then input to machine learning classifiers;^14^^,^^17^ third, supervised Deep Learning methods, which do not require handcrafted features, but automatically learn useful features directly from the input data.^18^^,^^19^ Works in the first category typically achieved lower accuracy than methods using supervised machine learning, because they tend to generalize less well to unseen cases. However, these do not rely on manually segmented labels for model training and can therefore be applied successfully to situations where a large labeled dataset is difficult or impossible to obtain, as for example in the case of rare pathologies. Acquiring training labels is typically an expensive step in any machine-learning pipeline, and data scarcity led models in the third category to poor generalization. Models in the second category can represent a compromise between those in the other two, attempting to strike a balance between data efficiency and generalization. These models are well suited in clinical contexts where labelled data is scarce. Our results suggest that the combination of information from different image modalities can generate a new class of handcrafted features, which could help improve the performance of this category of models.

Our methods and the study used to evaluate them have some limitations.

Although the proposed method was able to train on a small dataset, calculated performance metrics can be affected by the small number of images available.

Our estimate for measured sensitivity (0.83) indicates a limitation of our automated segmentation technique. This suggests that our technique was unable to identify all pixels that were classified as abnormal by the manual approach. This may be due to the variable reflectivity of larger lesions in the IR, which could have confused the classification model, resulting in the underestimation of some atrophic areas.

One limitation of blue AF images is the masking from the macular pigment near the fovea (see for example the misclassification of the foveal lesions in Fig. 2). The recent introduction of green AF (514 nm wavelength) could overcome this issue and provide better segmentation results^24^^,^^25^ using essentially the same methodology but simply pairing the IR with green (rather than blue) AF.

It should be recognized that the areas provided with our technique are estimates based on the manufacturer-provided conversion factor of 1 pixel = 0.01118 mm^2^. This is an average conversion factor, because the actual area that pixels equate to will vary slightly between patients. Although this means that there is some uncertainty in these estimates of area if comparing between patients, these conversion factors will remain constant within the same patient, and therefore the primary objective of monitoring disease within the same patient will likely be unaffected.

In terms of implementation, the main limitation of the proposed method is the need for aligned images. The automatic alignment could only complete the task successfully on nine of the 18 eyes evaluated, and this is therefore an area that requires further development. Unless a more robust algorithm is available, manual alignment may be required in a significant number of cases. It should be recognized however that the cases selected in this series are likely to have been particularly challenging given the advanced stage of the condition for many of the selected eyes, and that alignment performance across an unselected population would be expected to be better than this. Additionally, although automatic alignment is preferred, manual alignment is not onerous because it is facilitated with the selection of only four control points per image (eight points per alignment) making the process fast enough even for a clinical setting. We suspect that most clinicians would find this a reasonable investment of time in order to gain better objective quantitative metrics of chorioretinal lesions that the technique provides.

Our case study illustrates the clinical application of the technique as applied to a patient in their 30s with PIC over a 3.5-year time period, which we have visualized in both static and dynamic graphical forms (Fig. 3 and Supplementary Video). This example shows how the quantification of the chorioretinal lesions enabled by our technique could bring greater precision to monitoring progression including a sharp increase in the expansion rate, which might have been missed by simple visual inspection. In PIC and other forms of sight-threatening posterior uveitis, treatment decisions depend on the evaluation of these chorioretinal inflammatory lesions.

In addition to its value to routine clinical practice, our approach may provide a sensitive, reliable, objective measure for clinical trials which include patients with PIC or other posterior uveitis syndromes. We think this is particularly noteworthy. Currently, most such trials “lump together” all forms of “posterior segment involving uveitis” (PSIU). One way of dealing with the wide variation in which these forms of uveitis may demonstrate disease activity is to include “new or active chorioretinal lesion” as part of a composite endpoint of “active disease” or “treatment failure.” This therefore reduces a complex disease process (chorioretinitis) to a binary variable based on a subjective evaluation. The technique we have described here would provide objectivity and enable a more nuanced approach to evaluating impact of any intervention in these conditions.

No test, whether diagnostic or monitoring, should be considered in isolation, but rather within the context of the care pathway it supports. Future work should include the evaluation of this method within its testing pathway, with consideration of the actual consequences of the provision of this test data to clinicians, the treatment decisions made and the short and long-term consequences of those decisions.

Automated segmentation of chorioretinal lesions using multimodal images shows closer alignment to traditional manual segmentation than segmentation based on AF only, as indicated by a high Dice's coefficient. The proposed technique provides an automatic and objective segmentation of chorioretinal lesions that could offer much-needed quantitative measurements in clinical practice as demonstrated by its performance in lesion detection, automated area estimation and progression tracking in the sight-threatening posterior uveitis syndrome, PIC.

## Supplementary Material

Supplement 1

Supplement 2
