# Supplementary figures and images for: Merging Information From Infrared and Autofluorescence Fundus Images for Monitoring of Chorioretinal Atrophic Lesions
Source: Transl Vis Sci Technol. 2020 Aug 25;9(9):38. doi: 10.1167/tvst.9.9.38 (PMC7453042; doi:10.1167/tvst.9.9.38)

| AF AND IR                                                                         | MANUAL                                                                              | AUTOMATIC<br>AF + IR                                                                | AUTOMATIC<br>AF                                                                      |
|-----------------------------------------------------------------------------------|-------------------------------------------------------------------------------------|-------------------------------------------------------------------------------------|--------------------------------------------------------------------------------------|
| 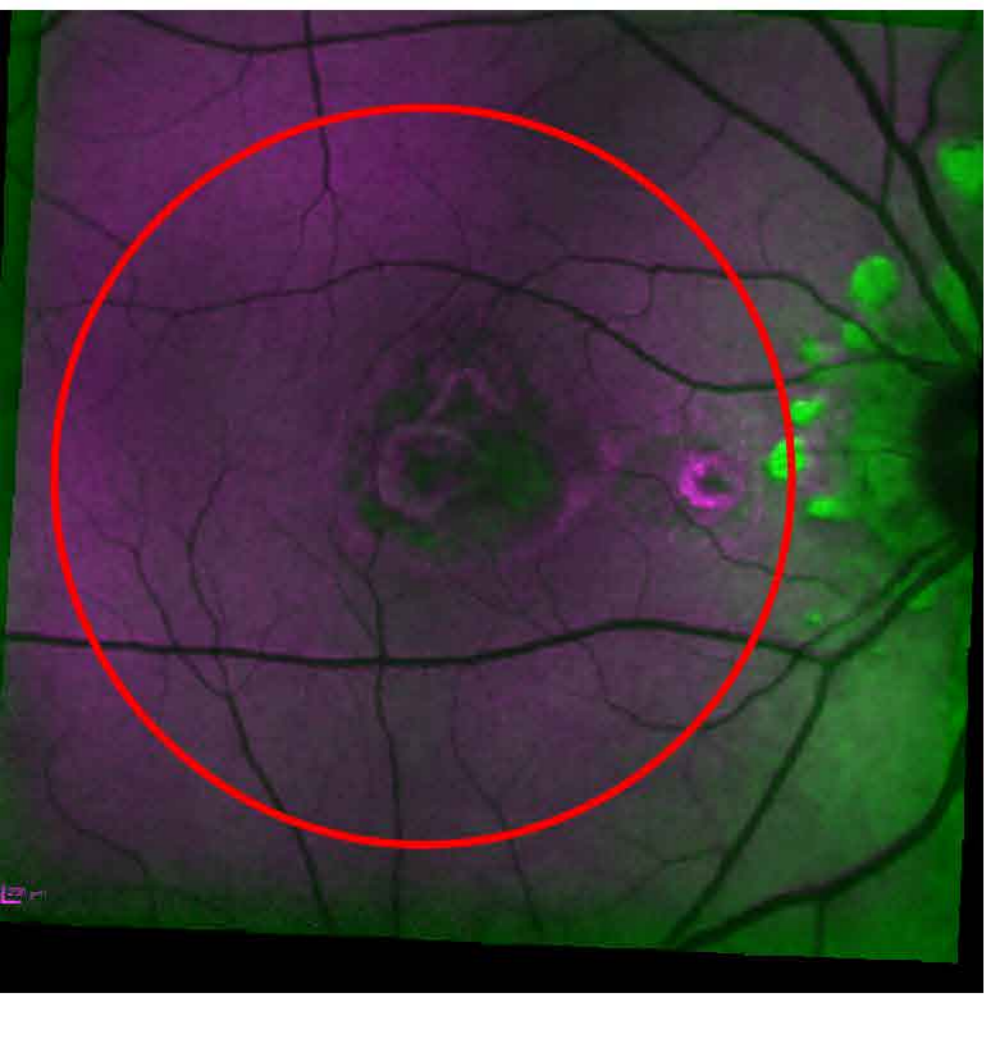    | 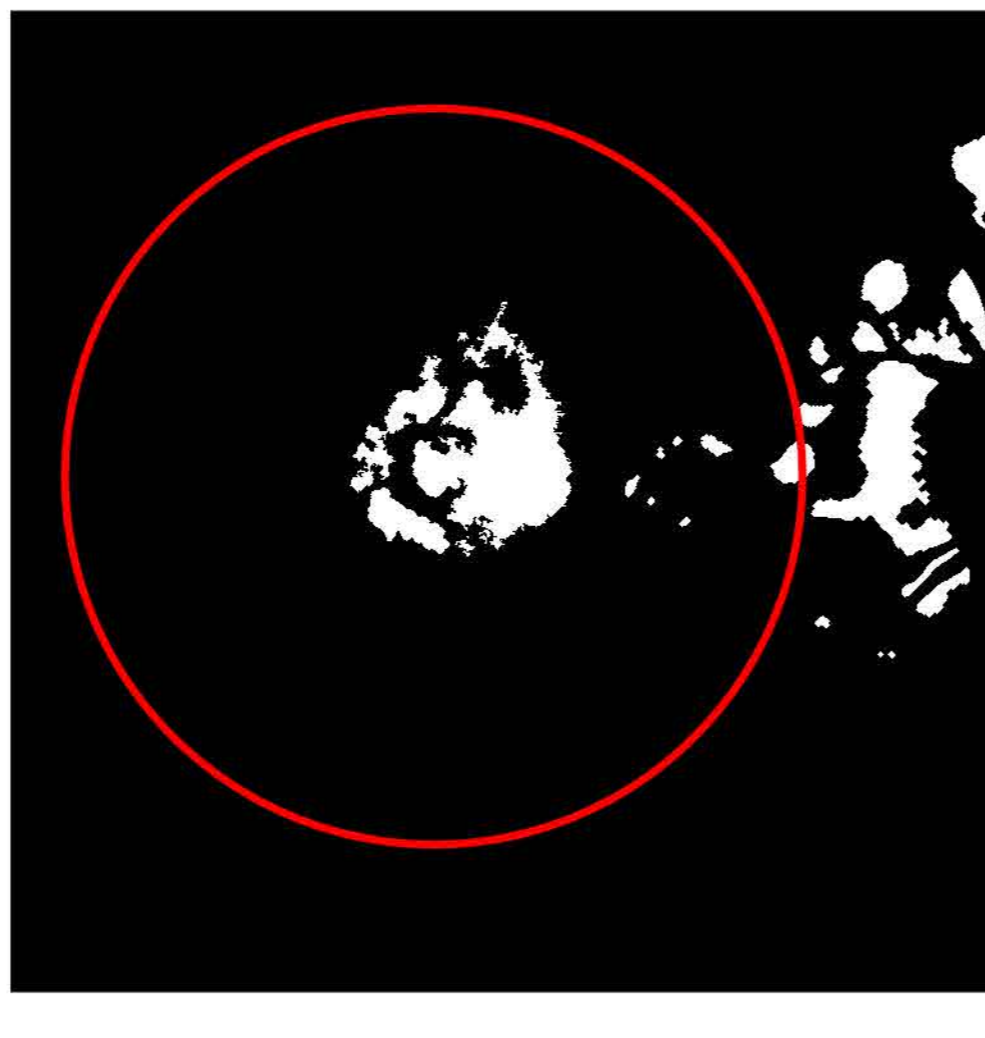    | 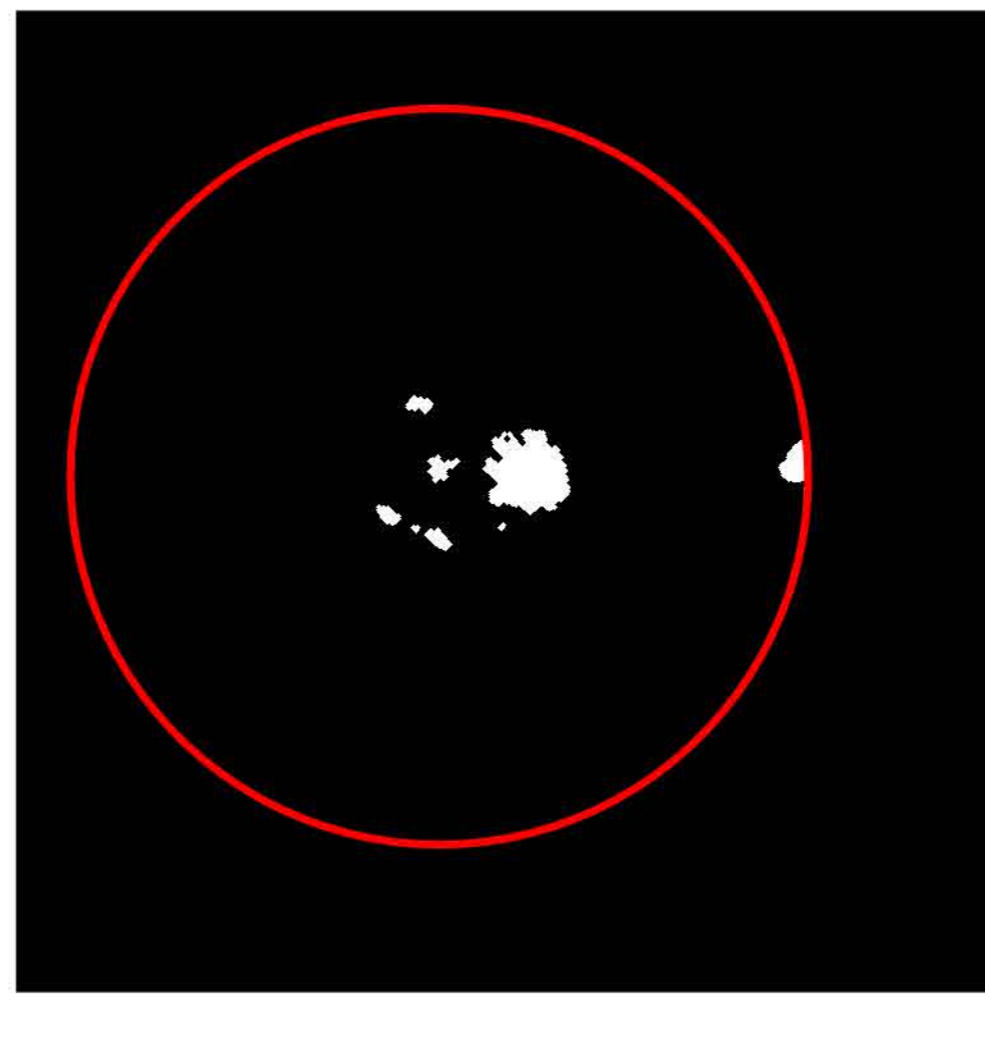    | 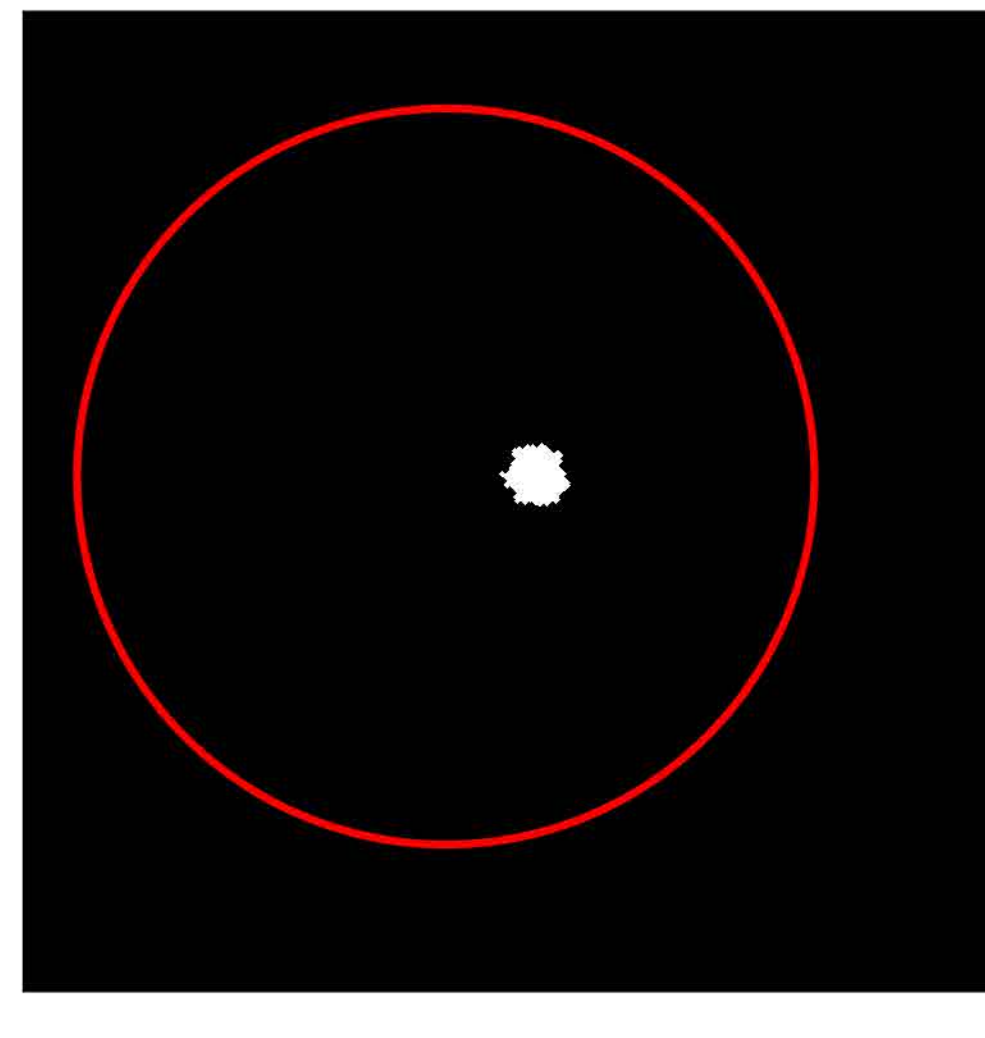    |
| 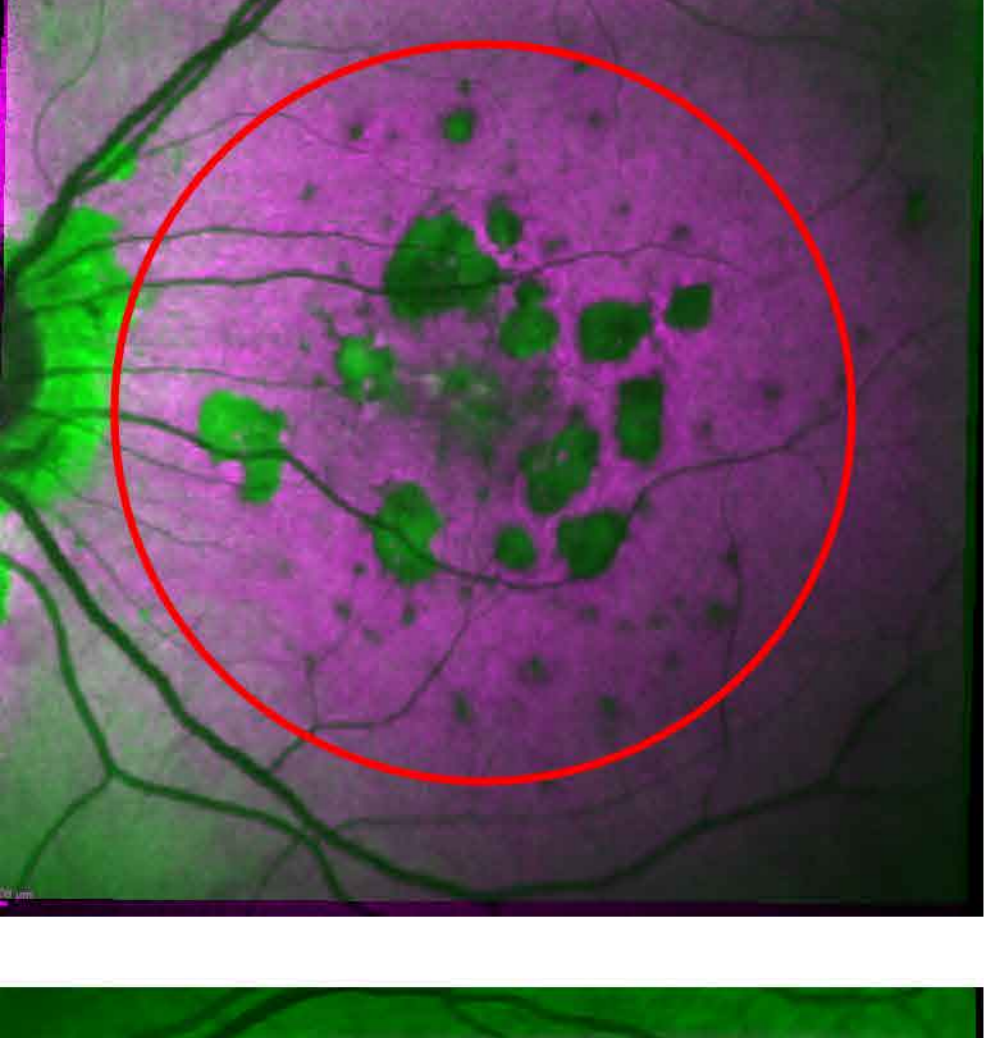   | 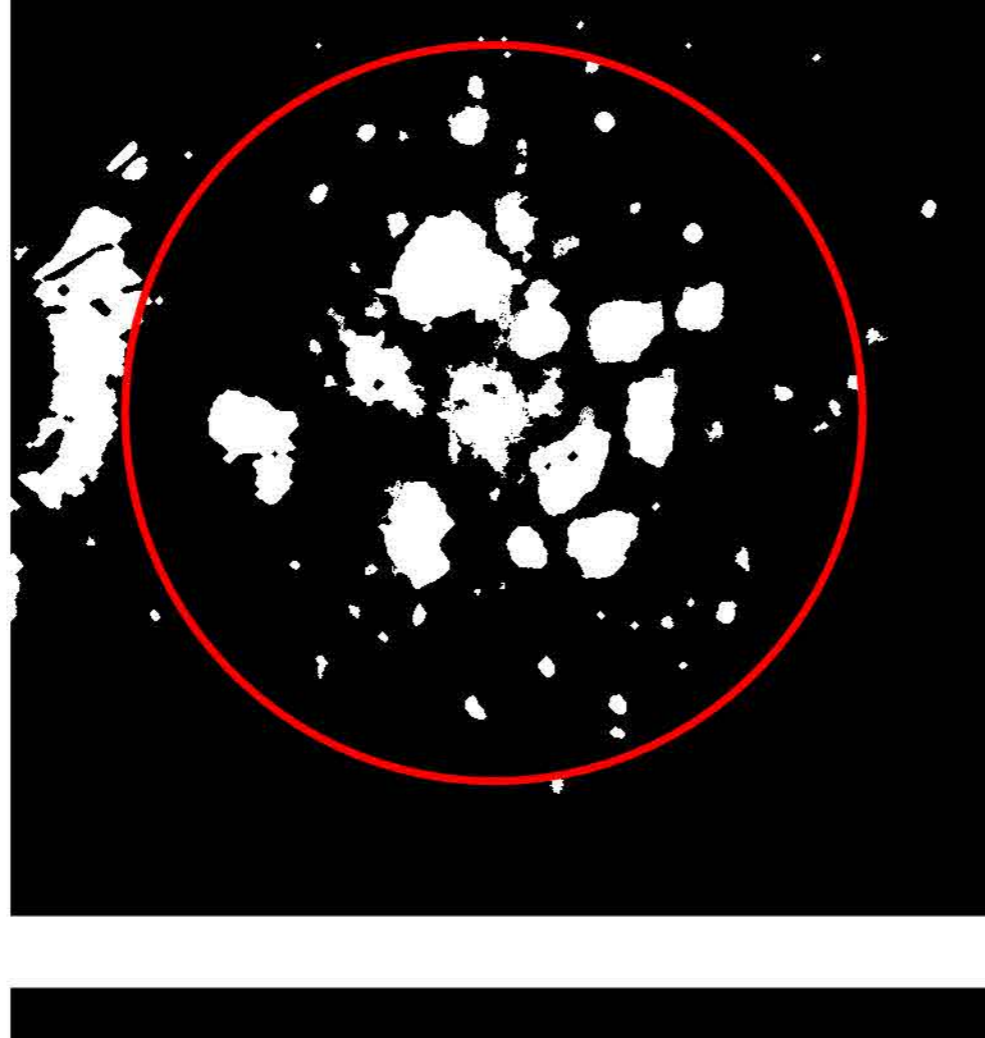   | 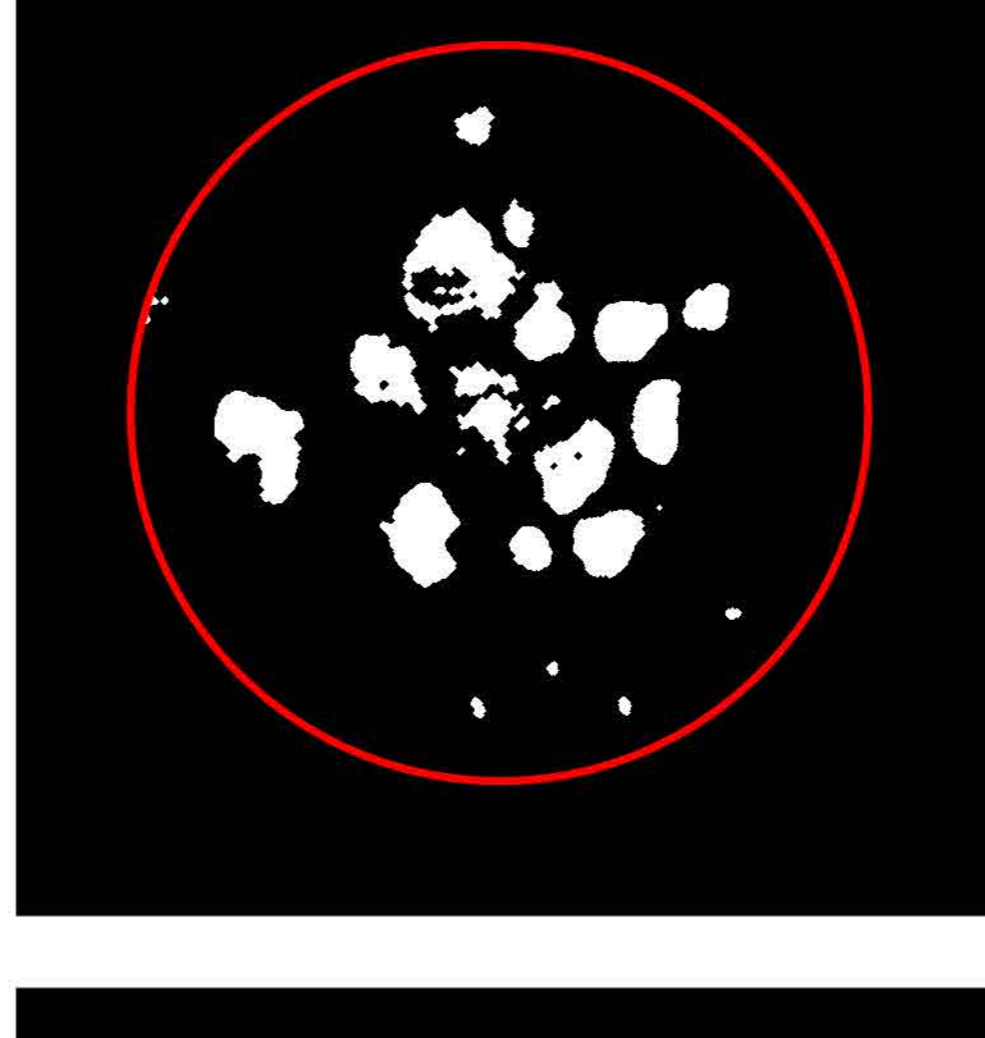   | 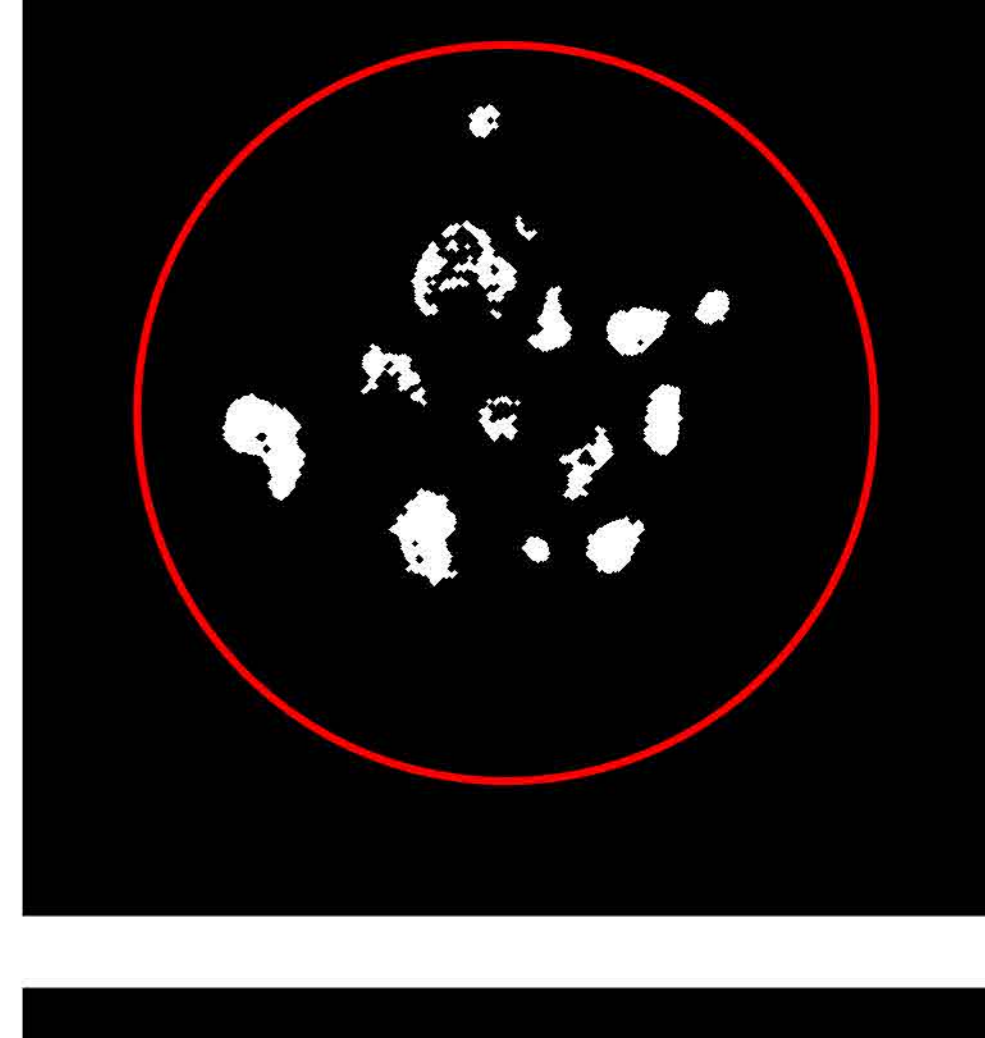   |
| 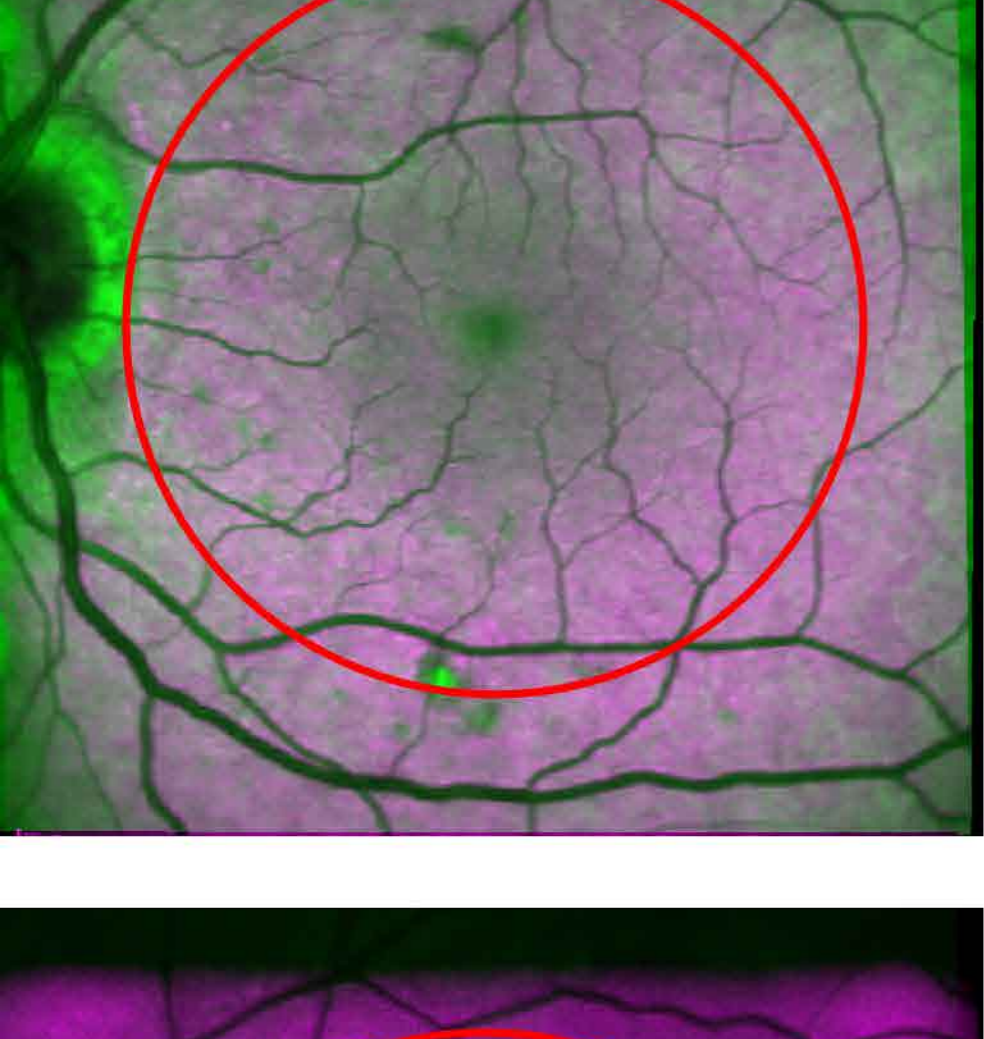  | 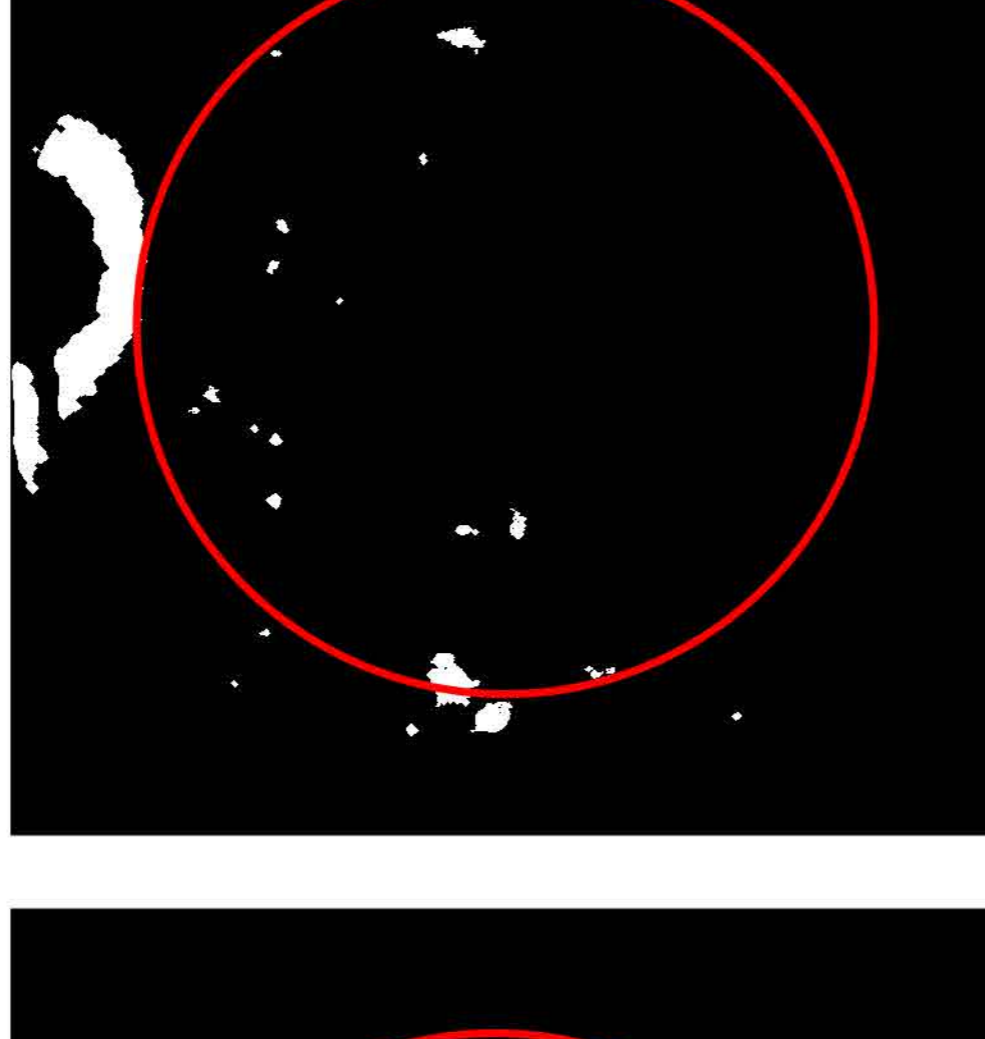  | 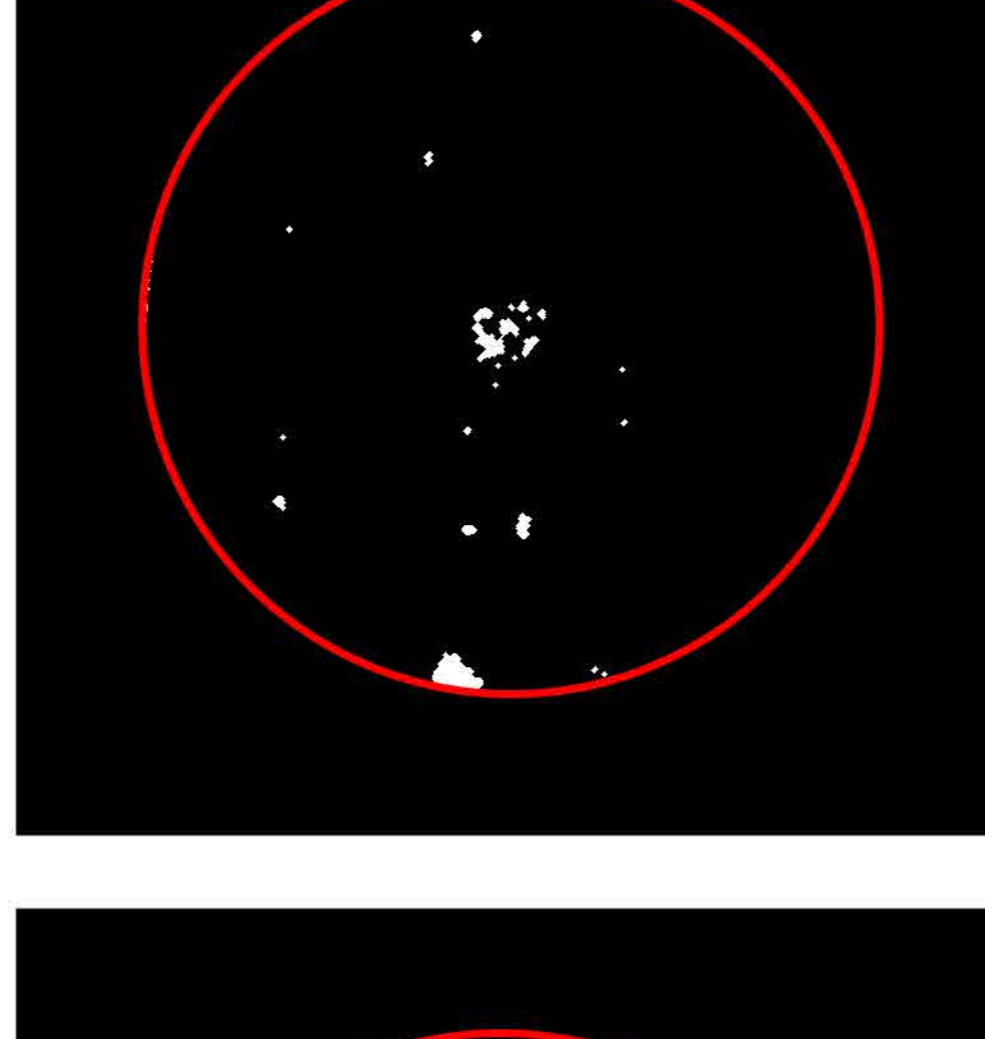  | 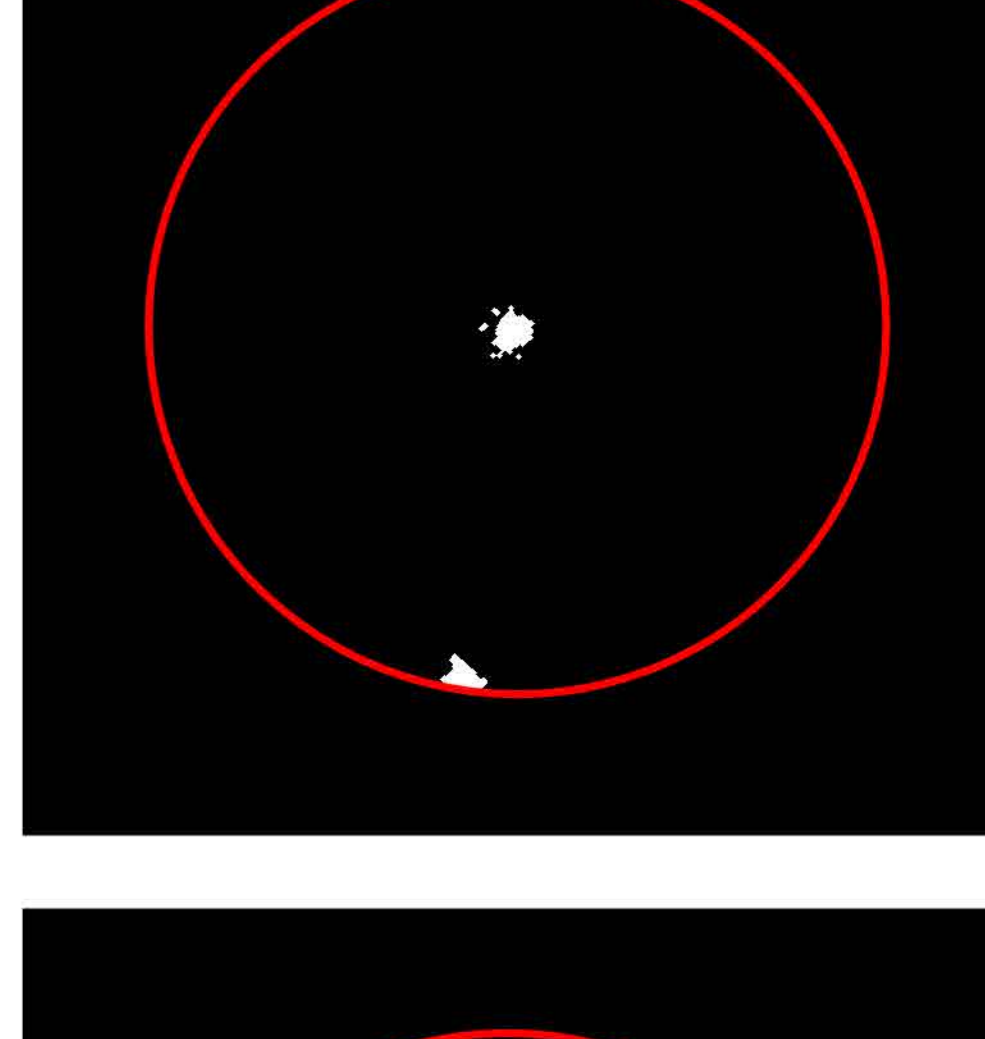  |
| 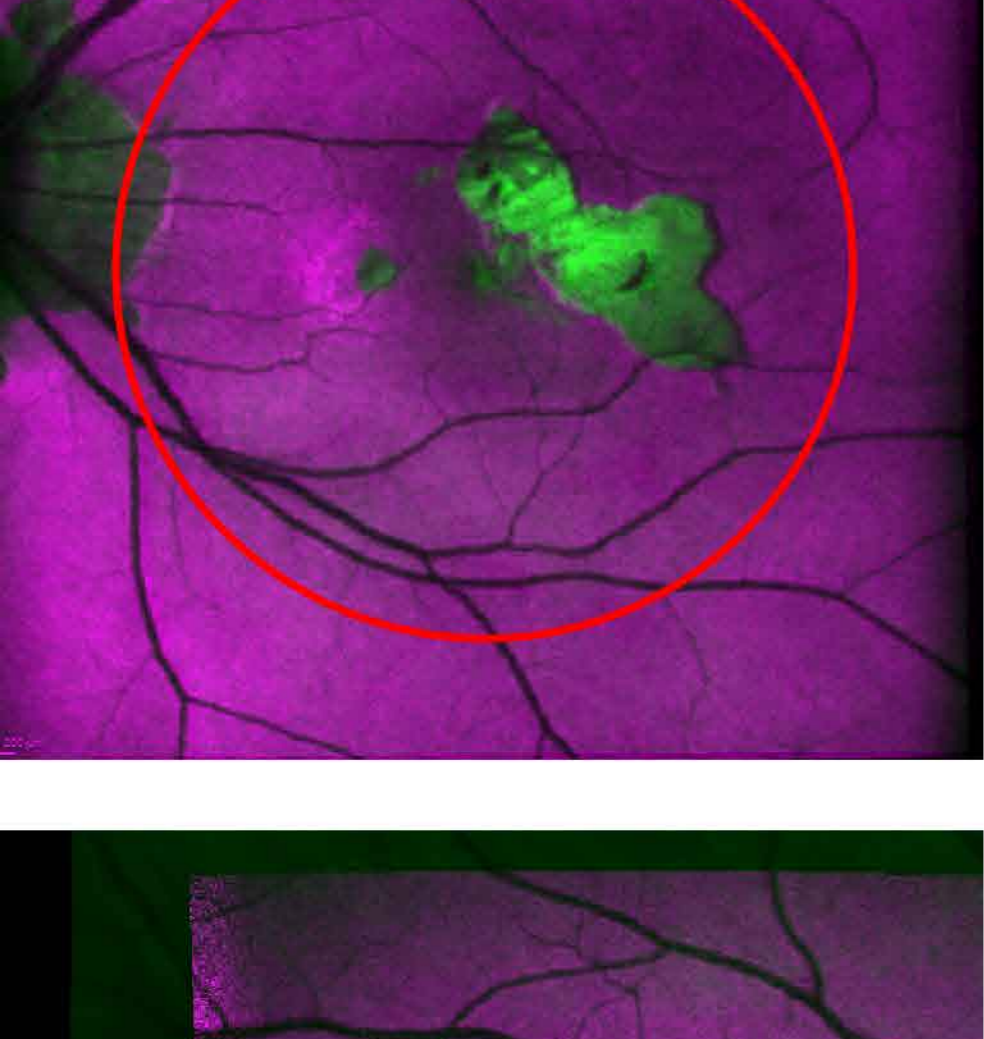 | 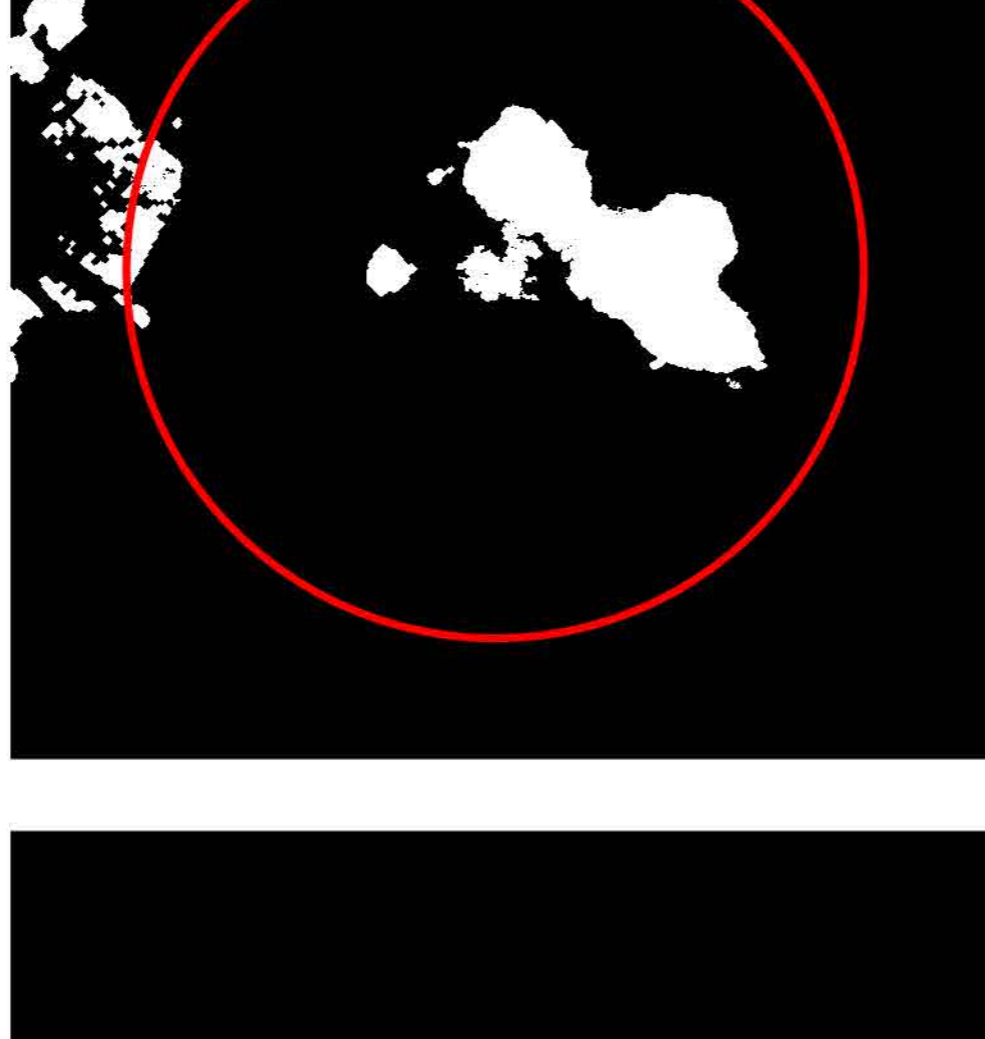 | 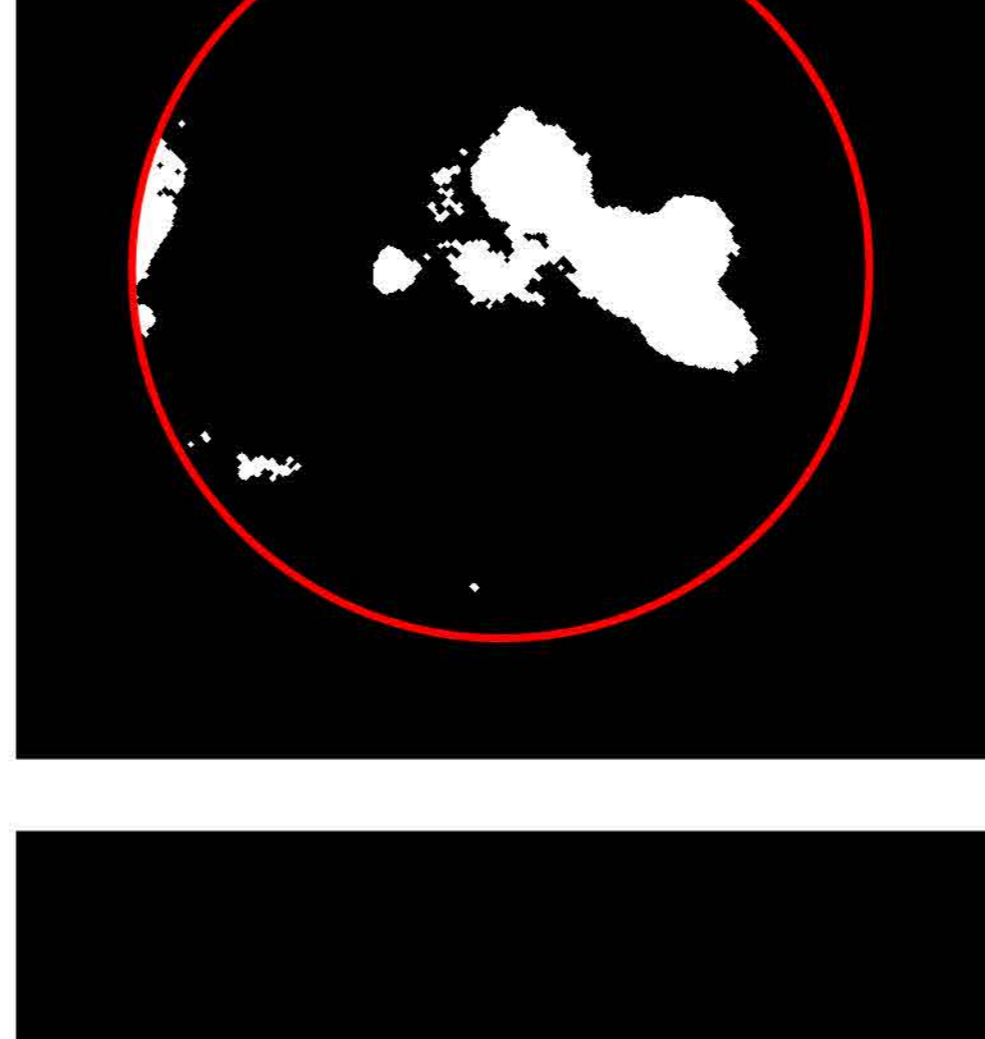 | 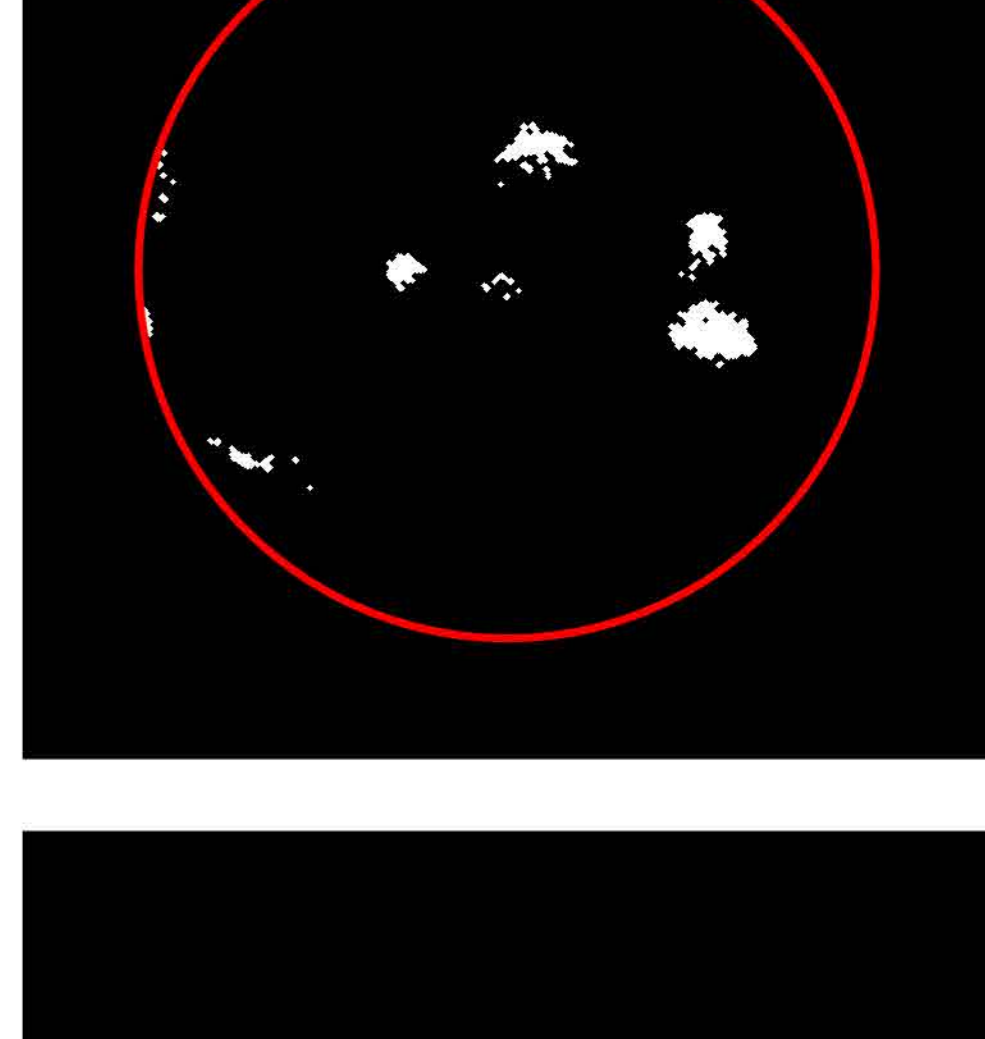 |
| 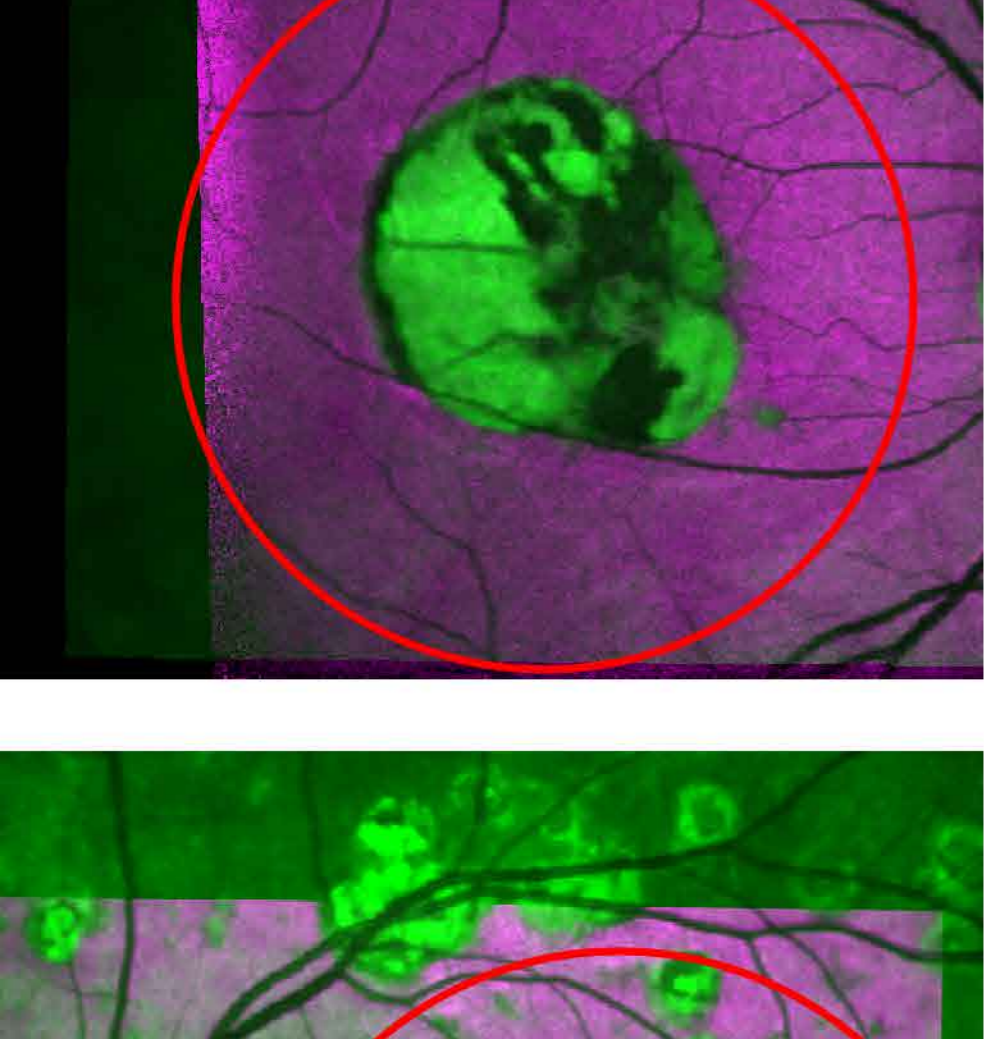 | 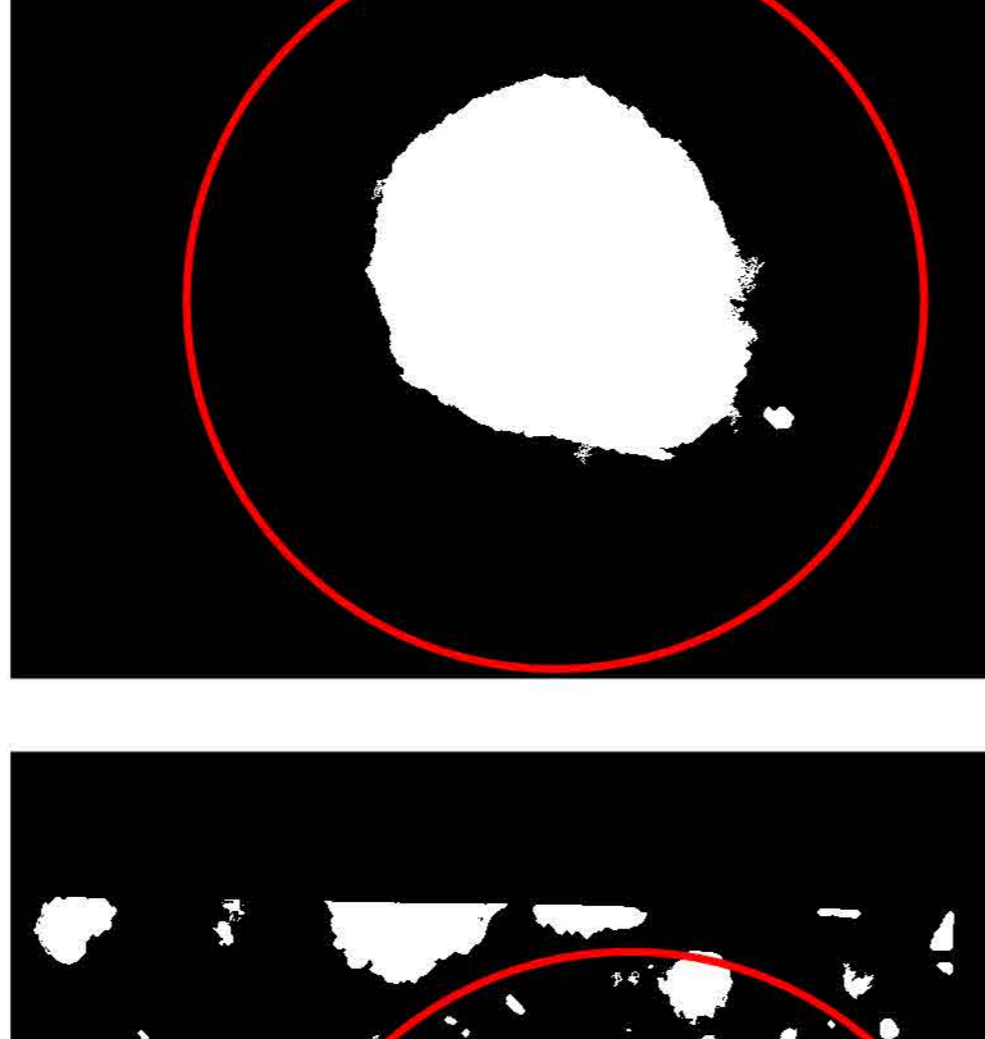 | 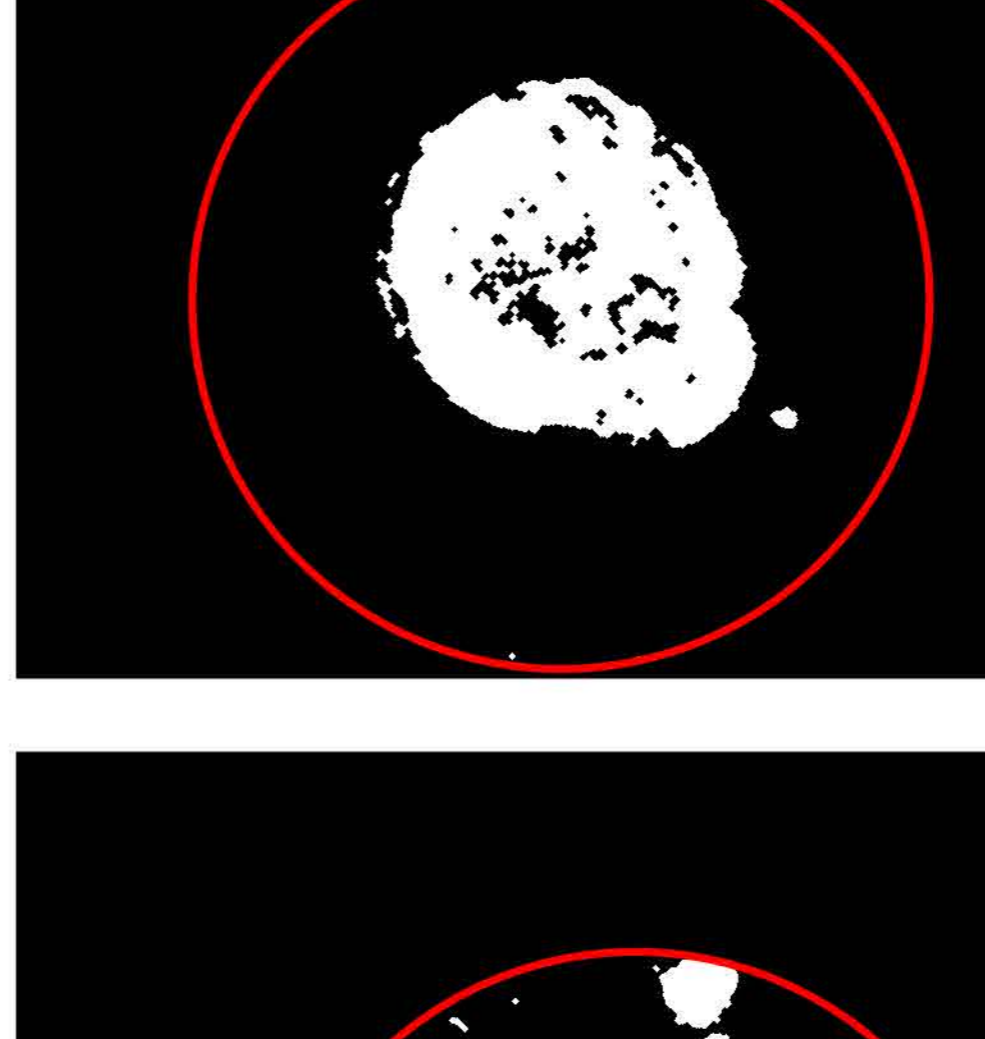 | 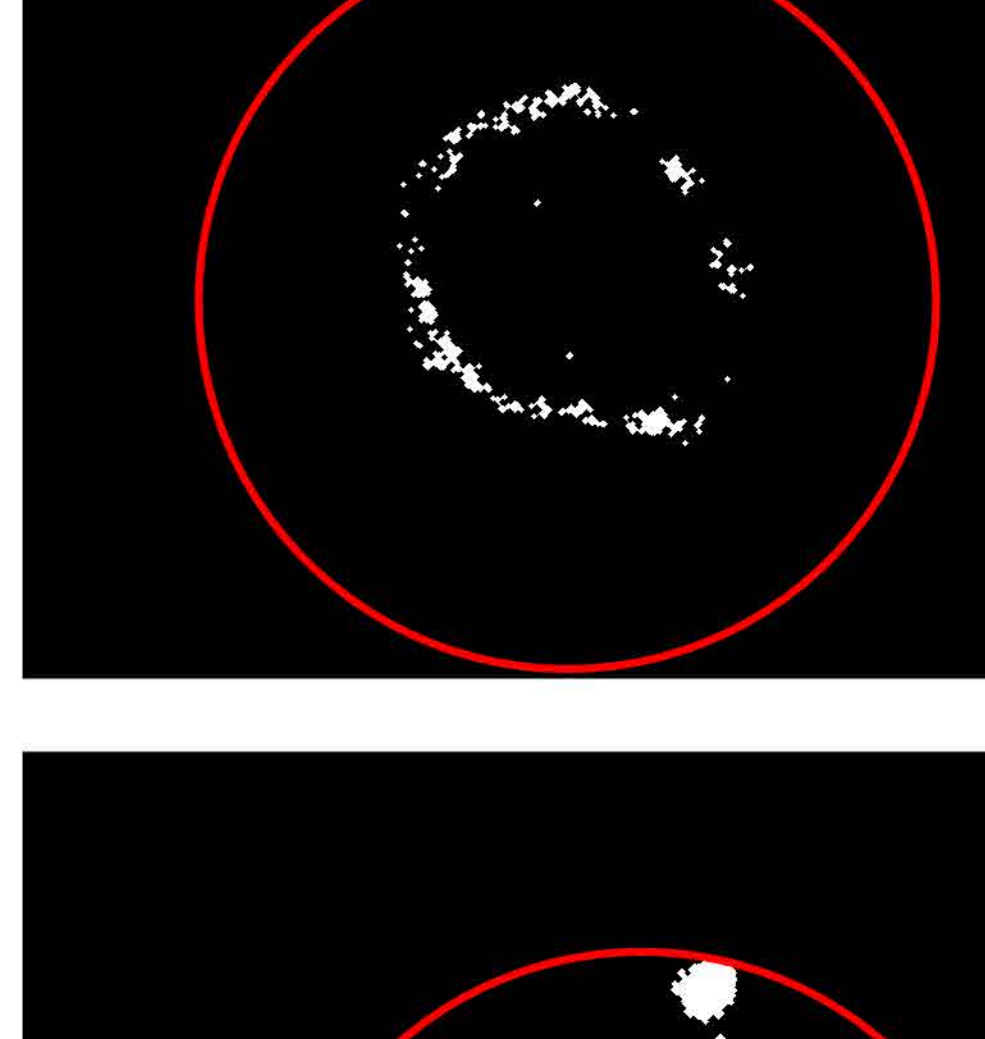 |
| 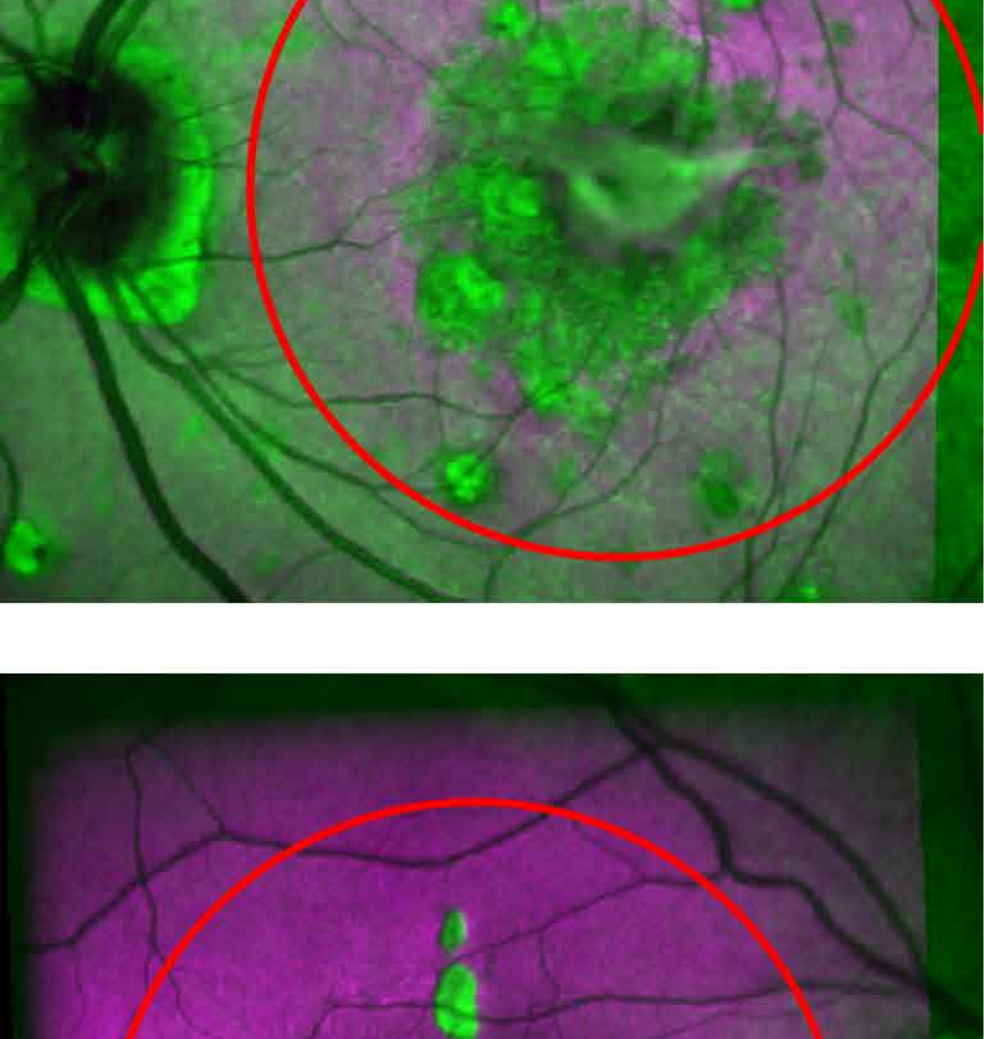 | 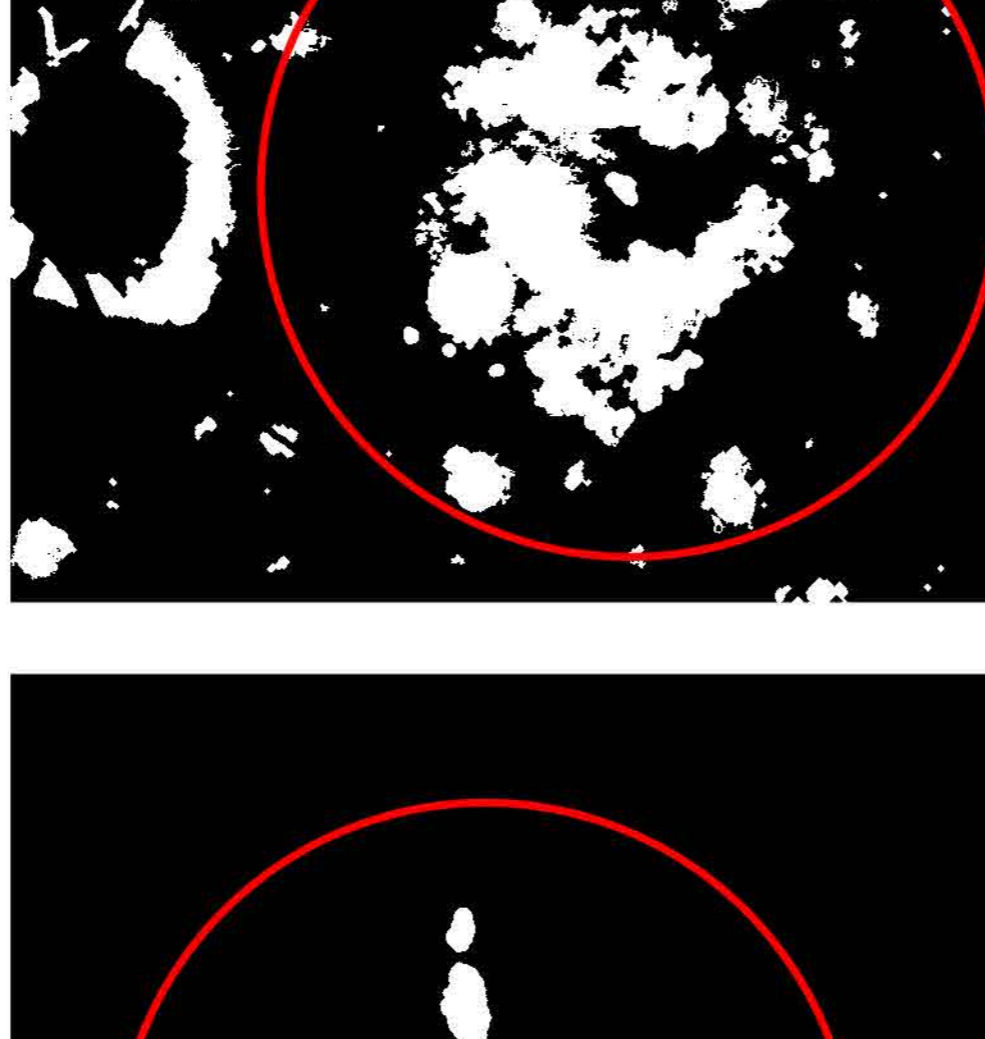 | 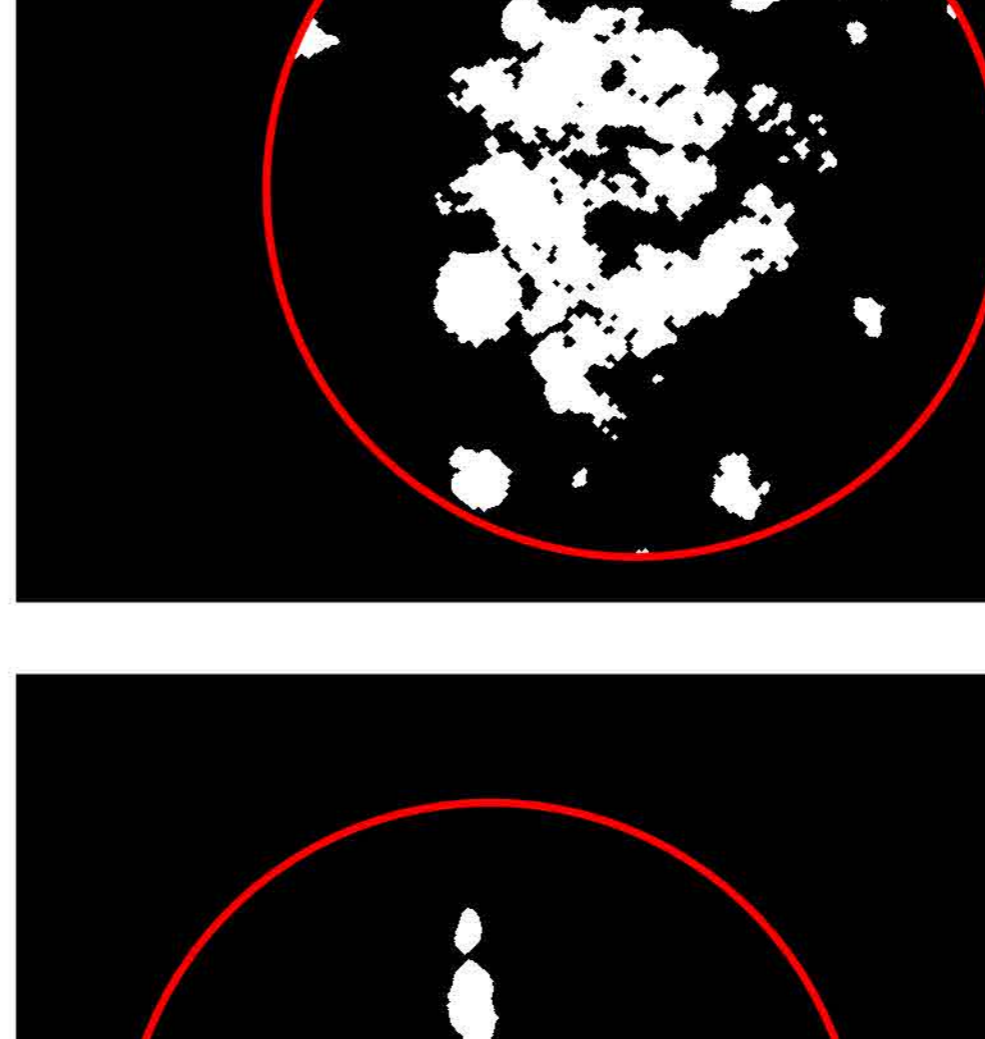 | 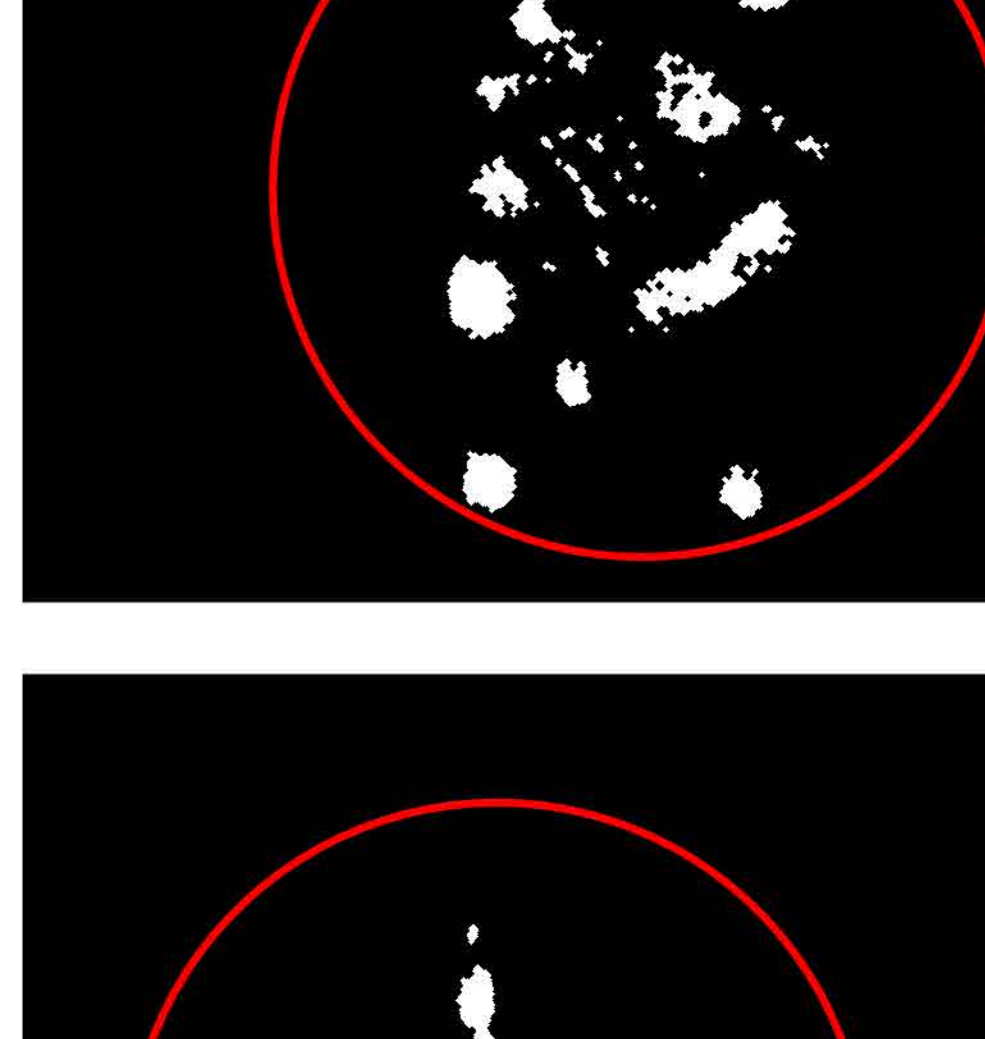 |
| 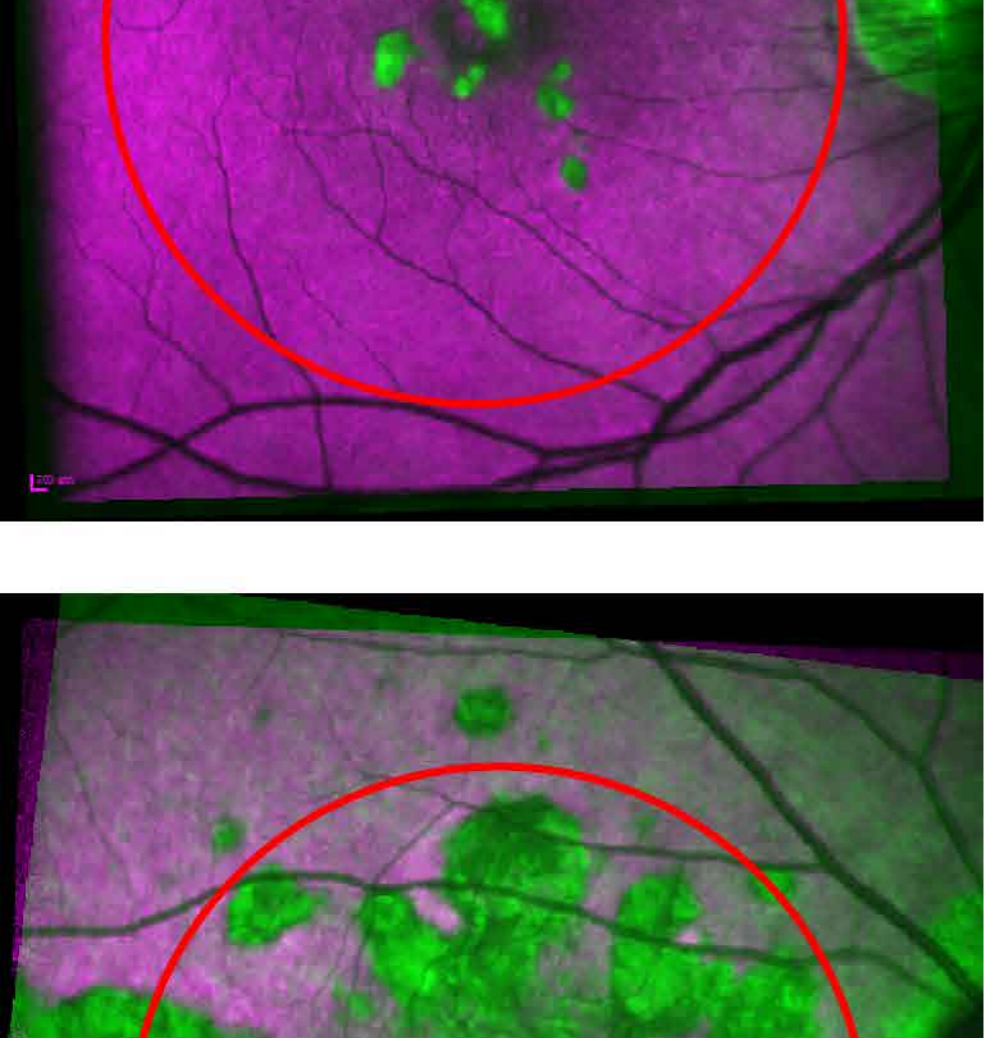 | 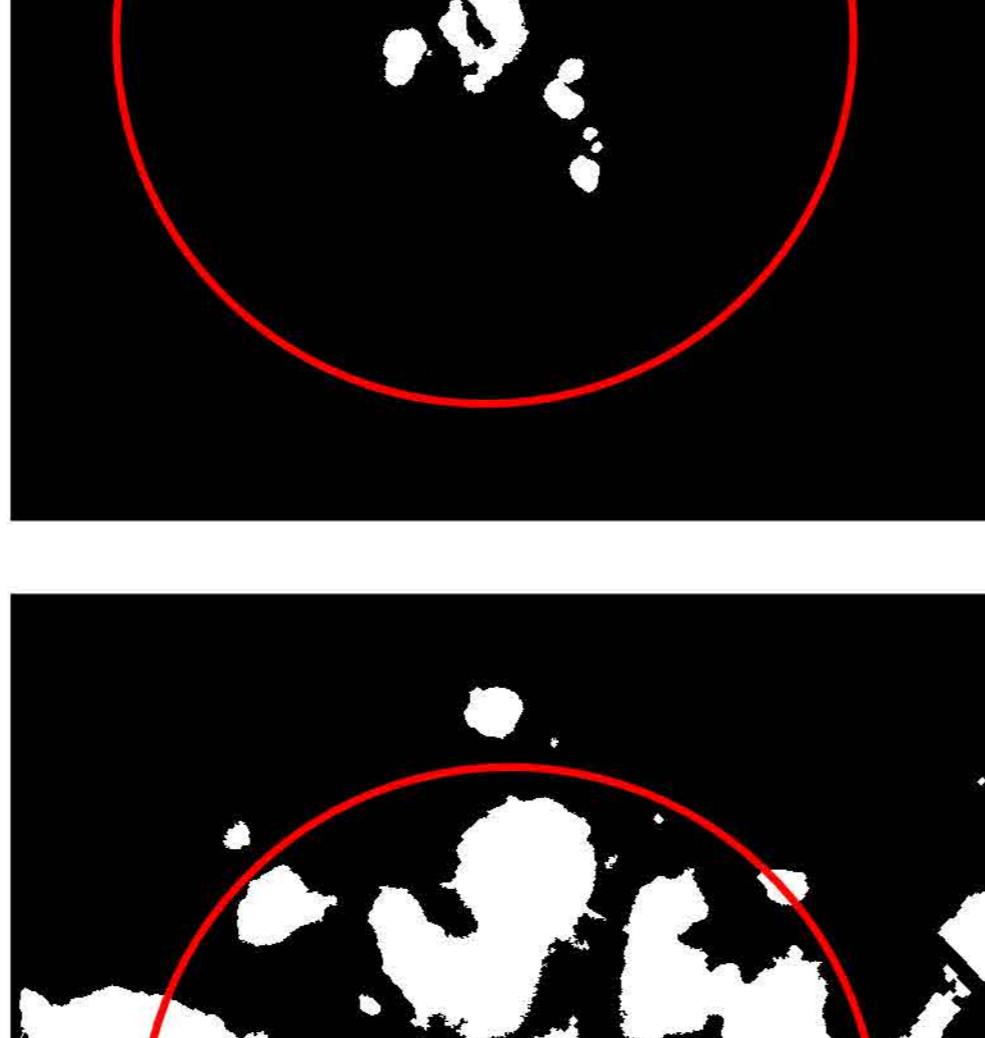 | 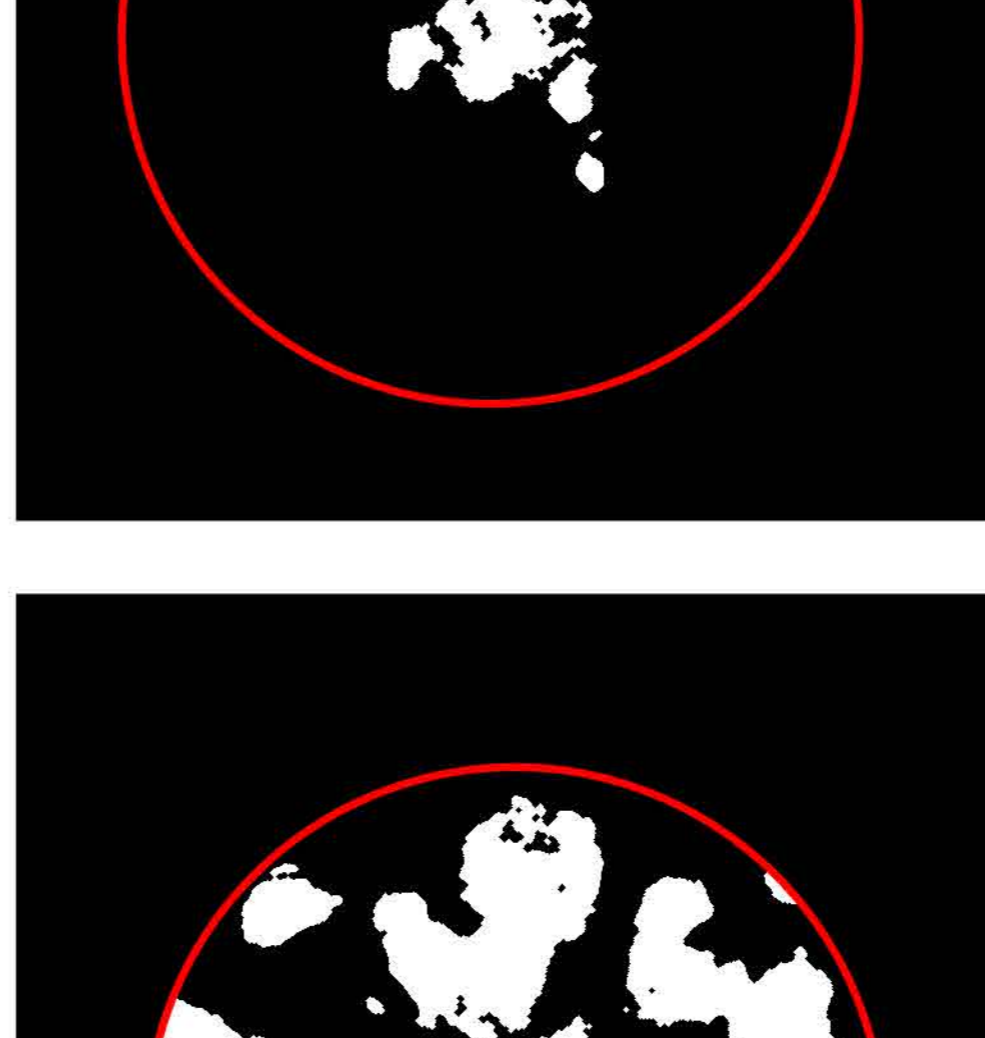 | 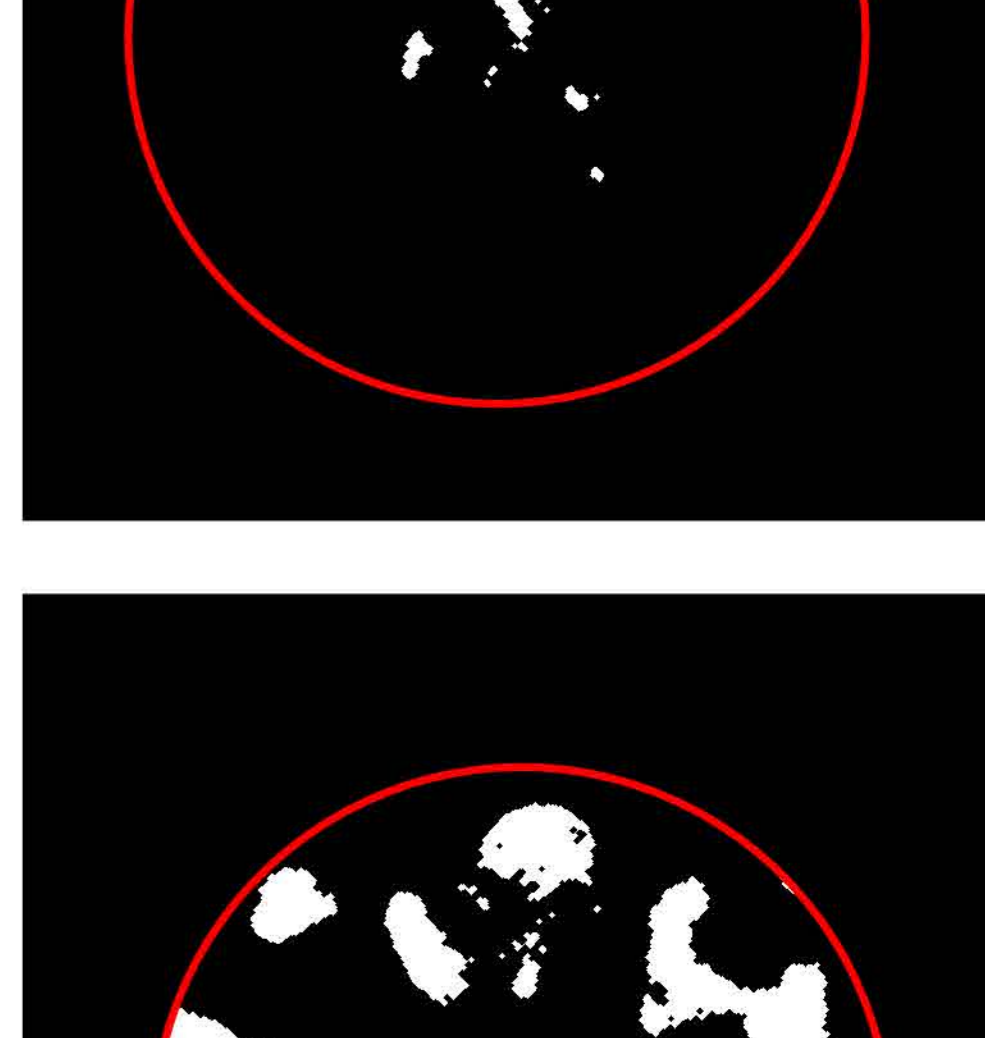 |
| 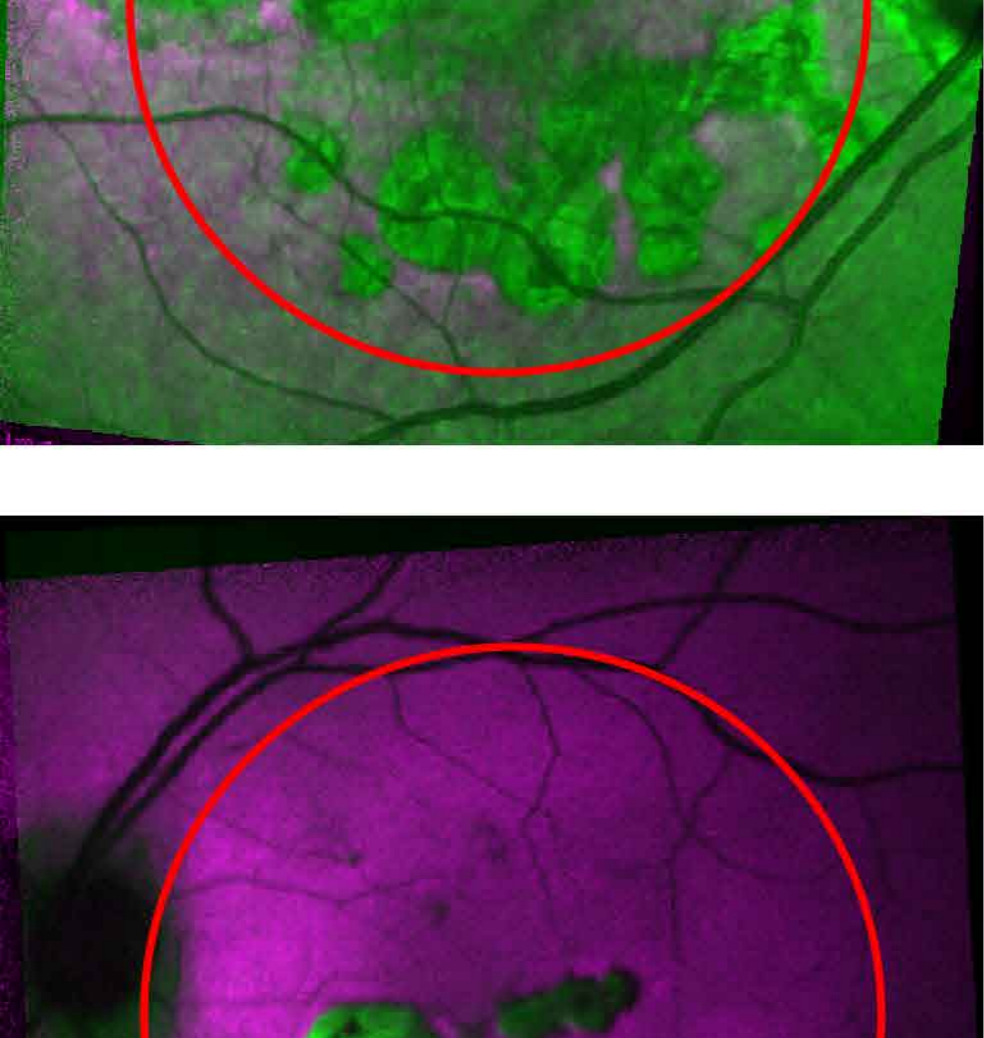 | 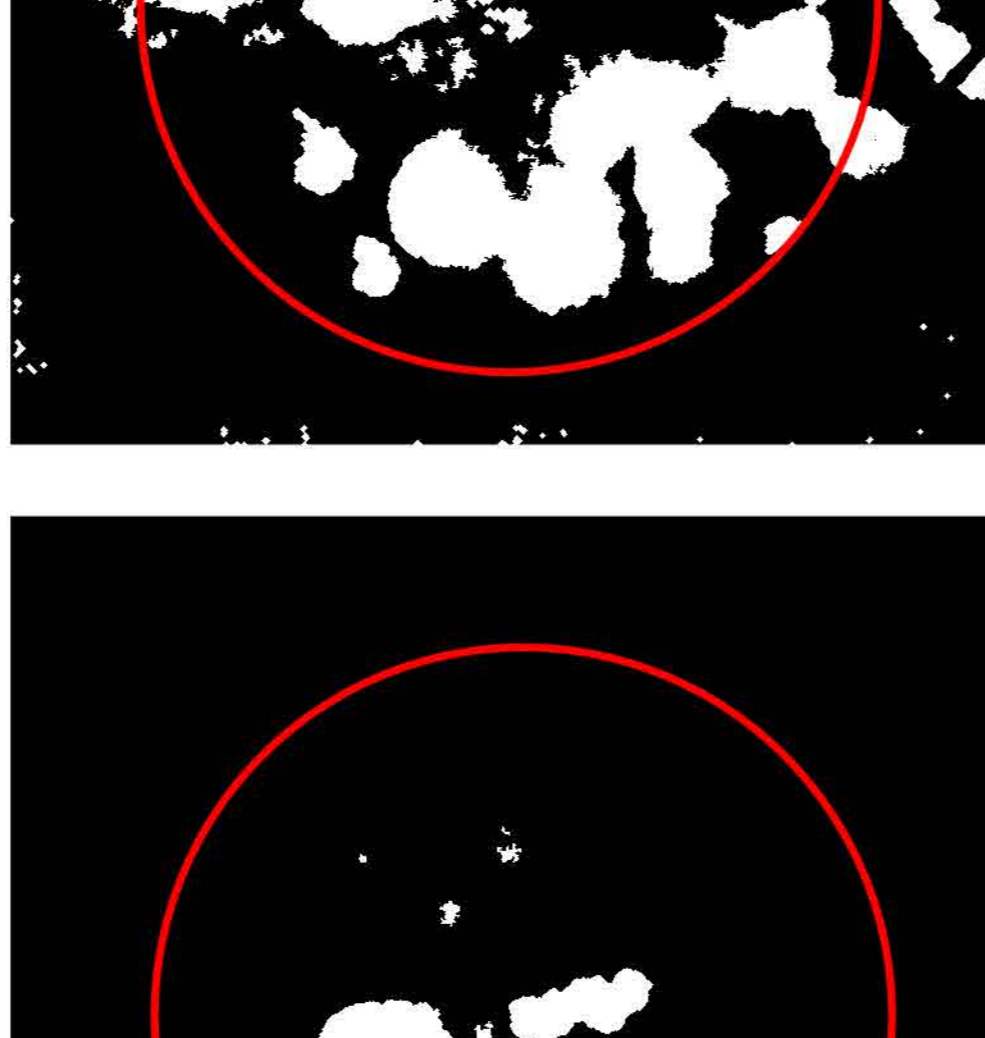 | 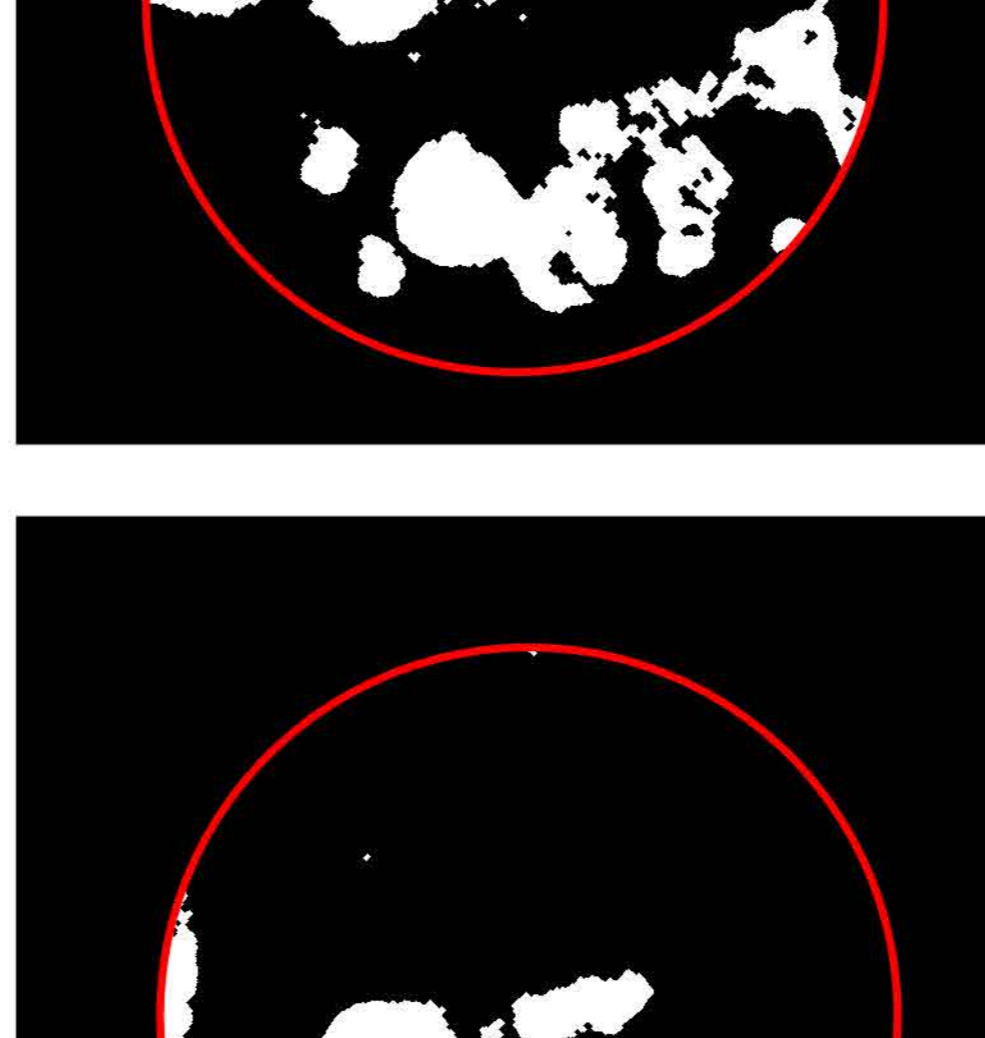 | 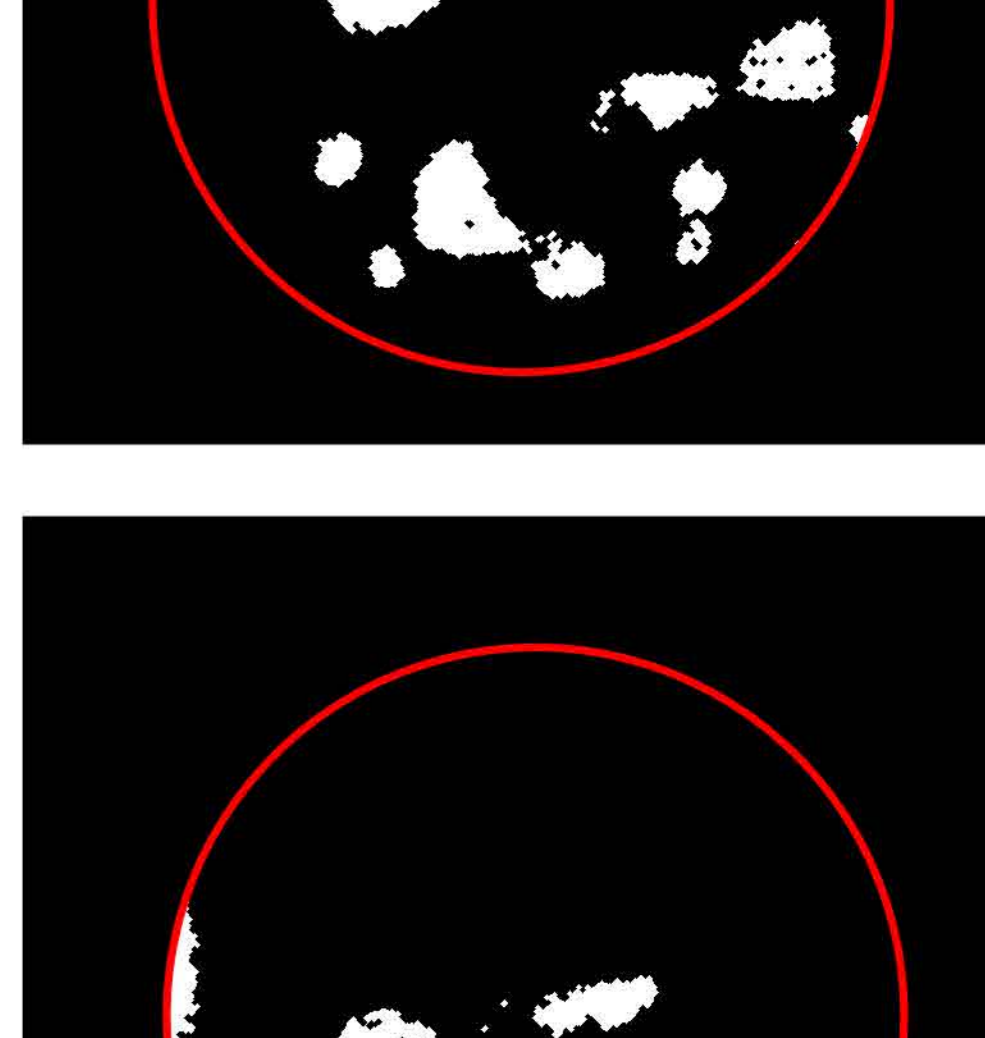 |
| 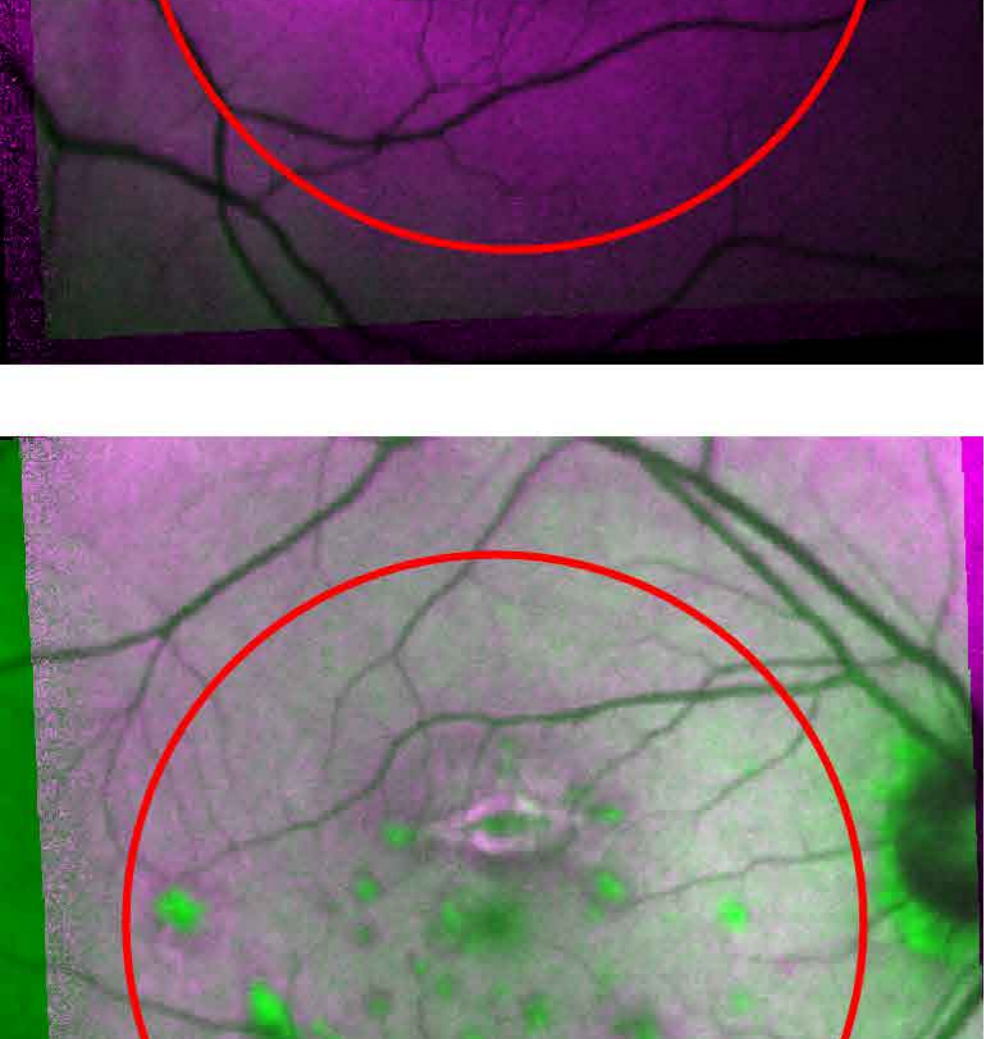 | 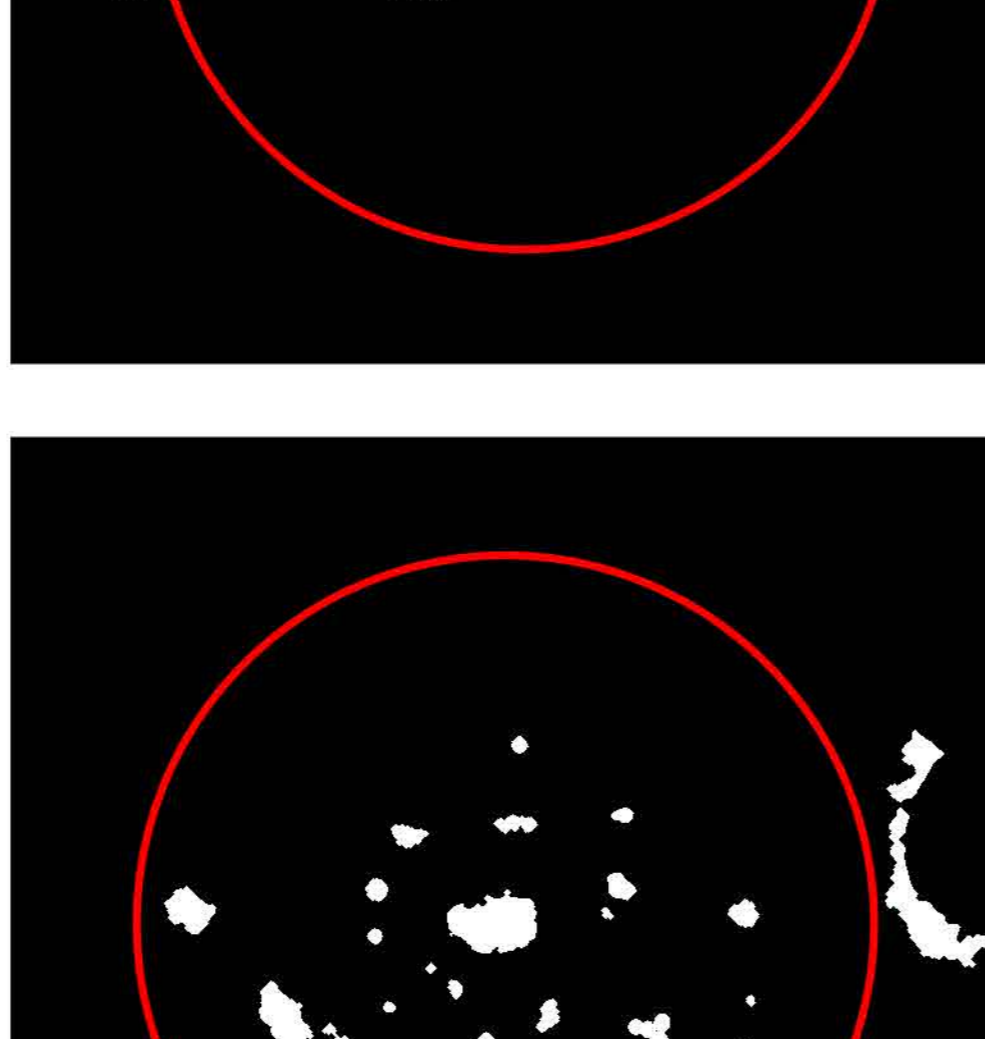 | 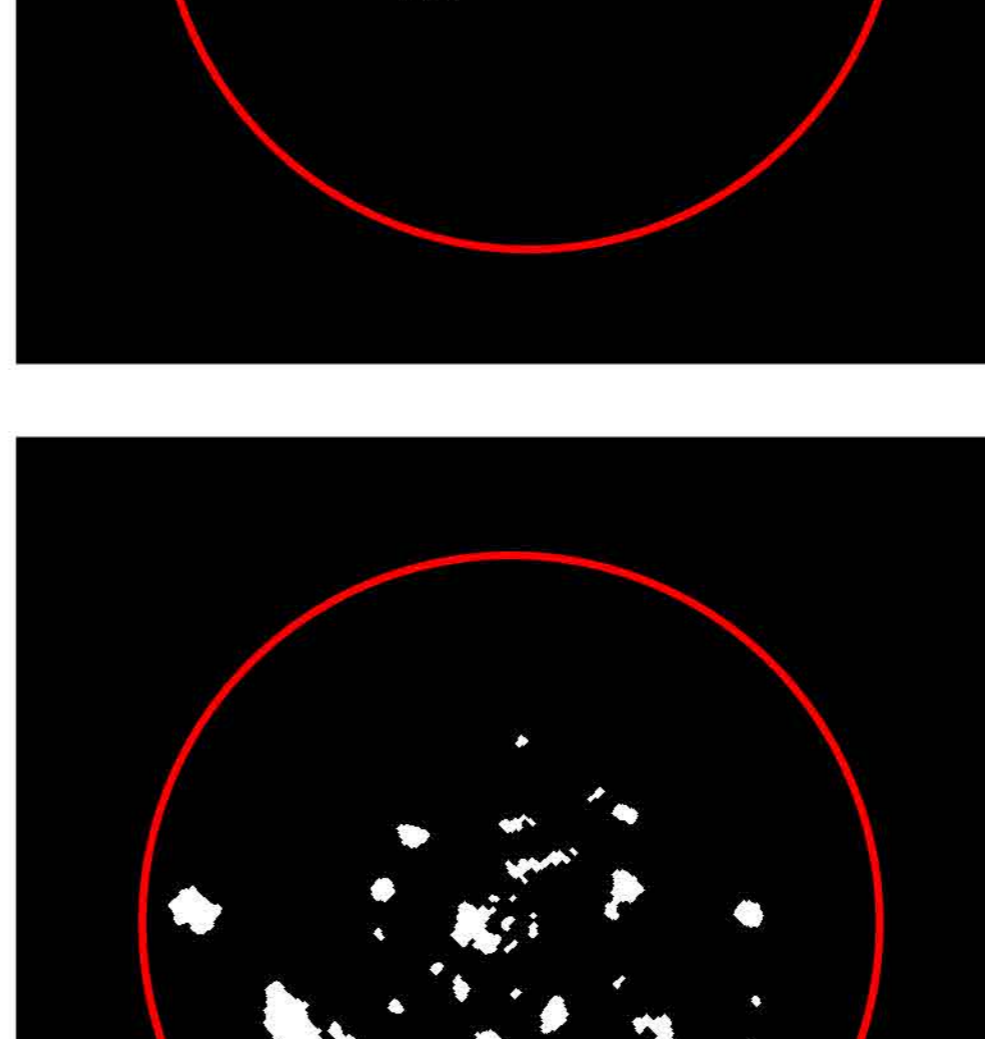 | 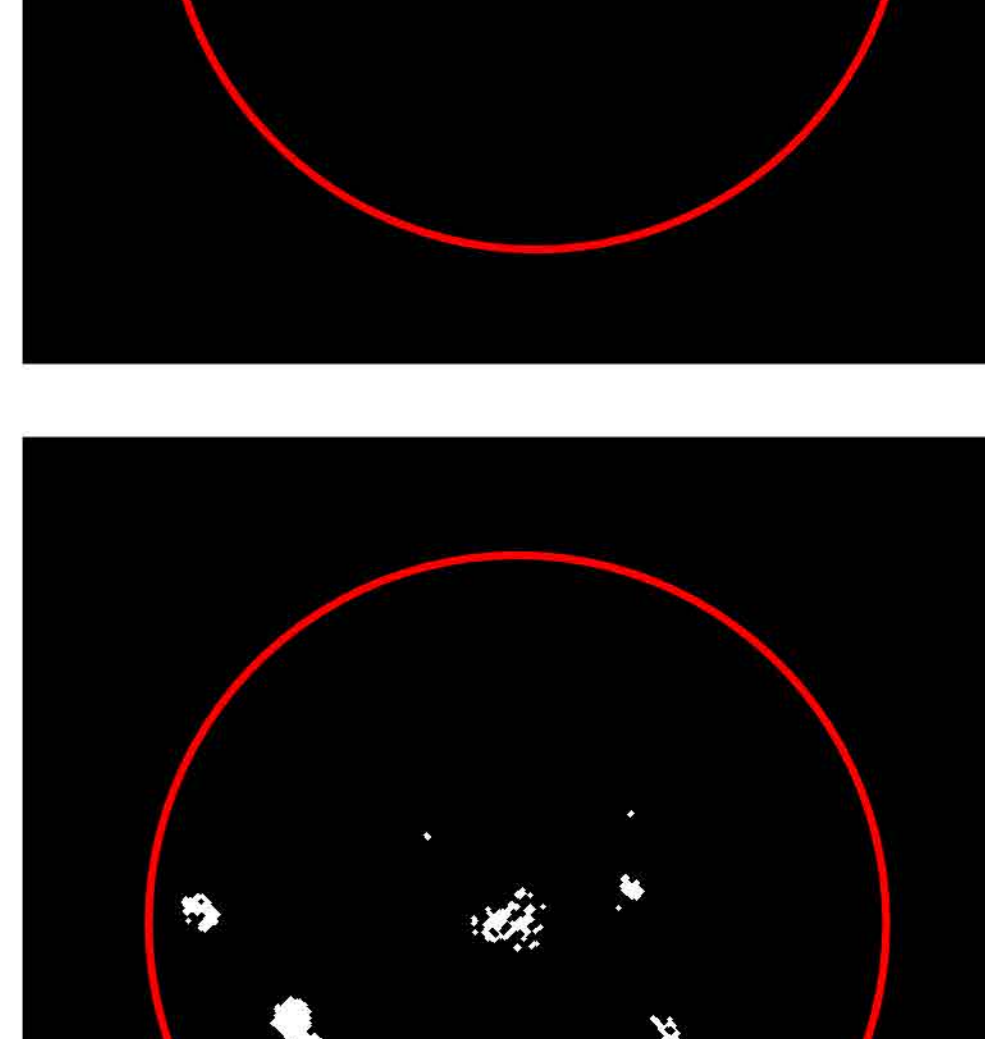 |
| 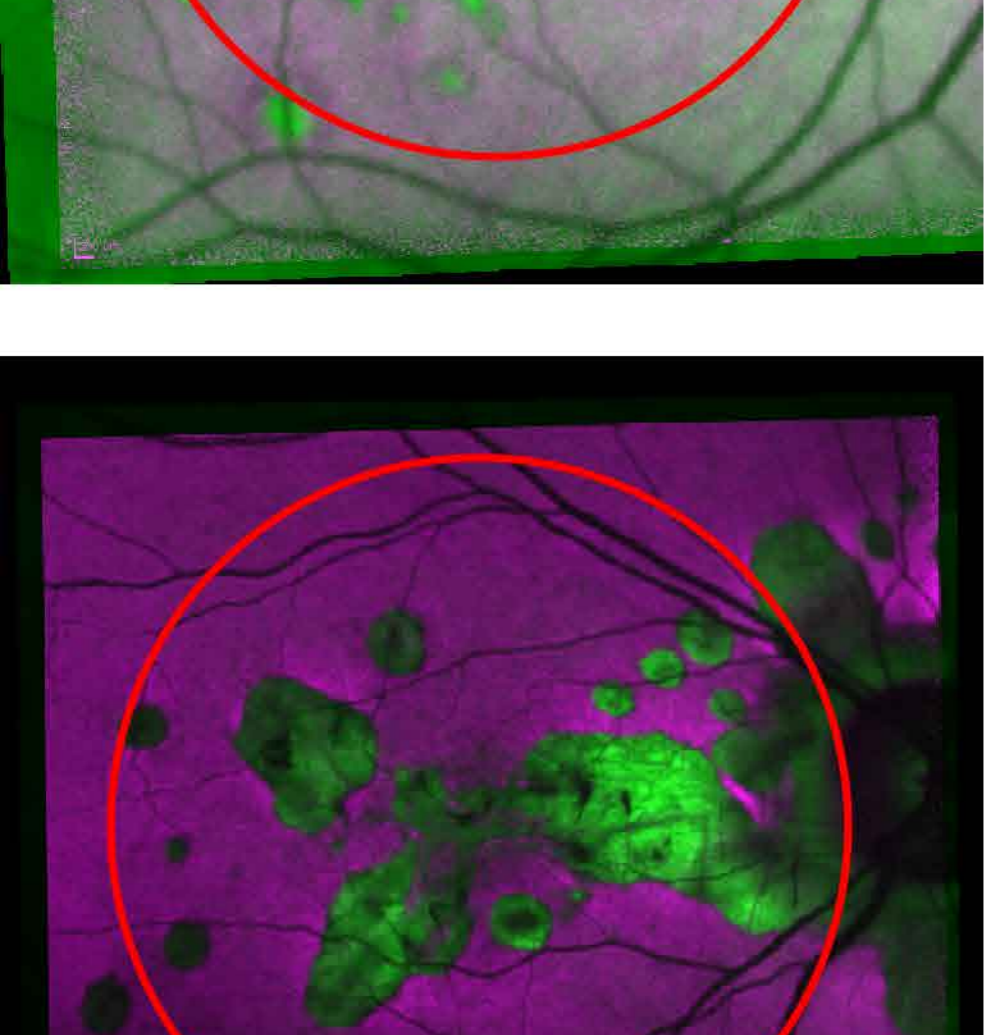 | 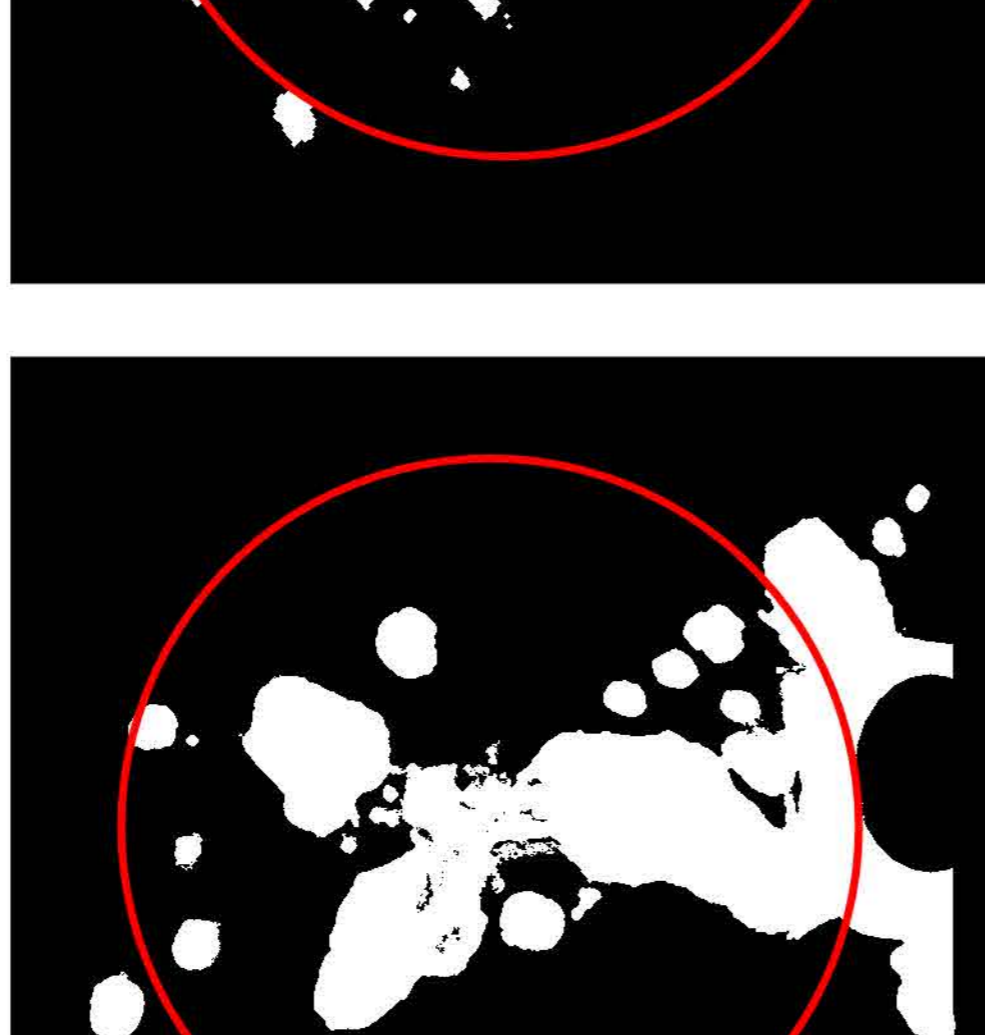 | 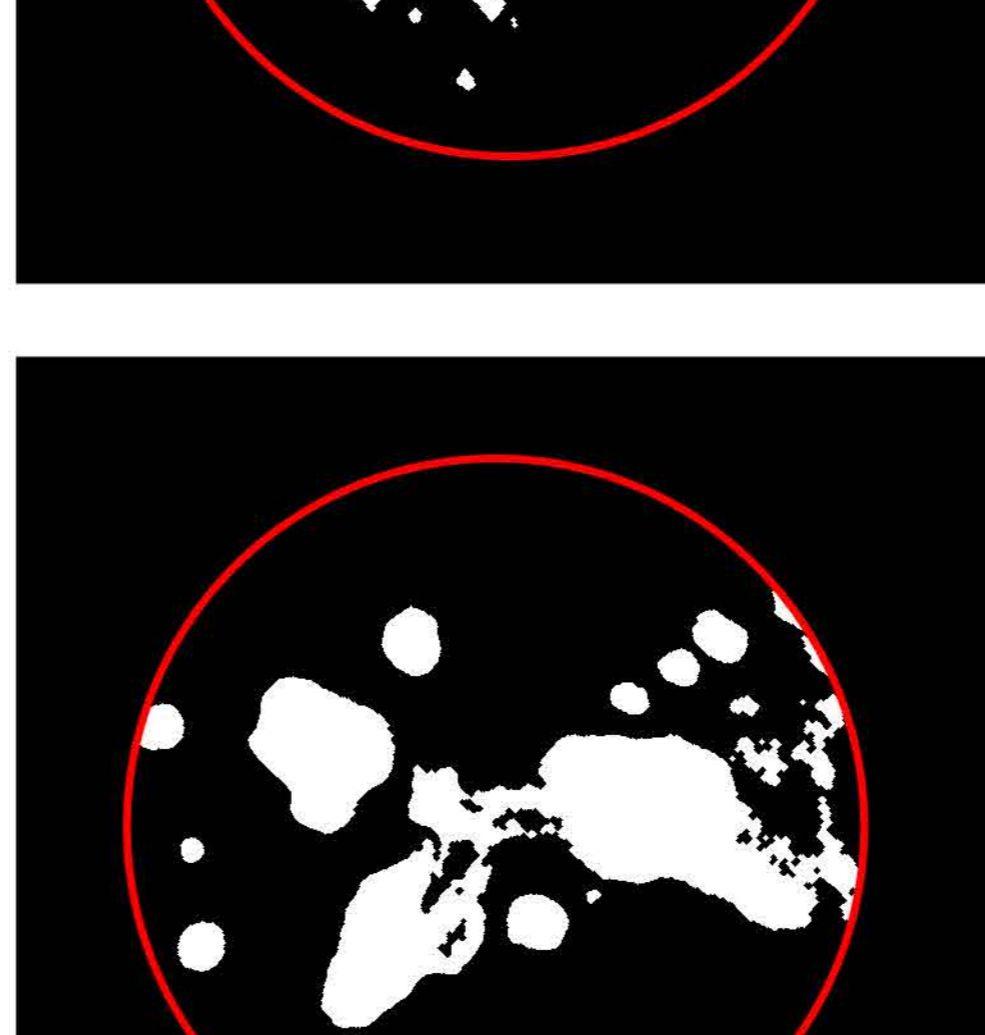 | 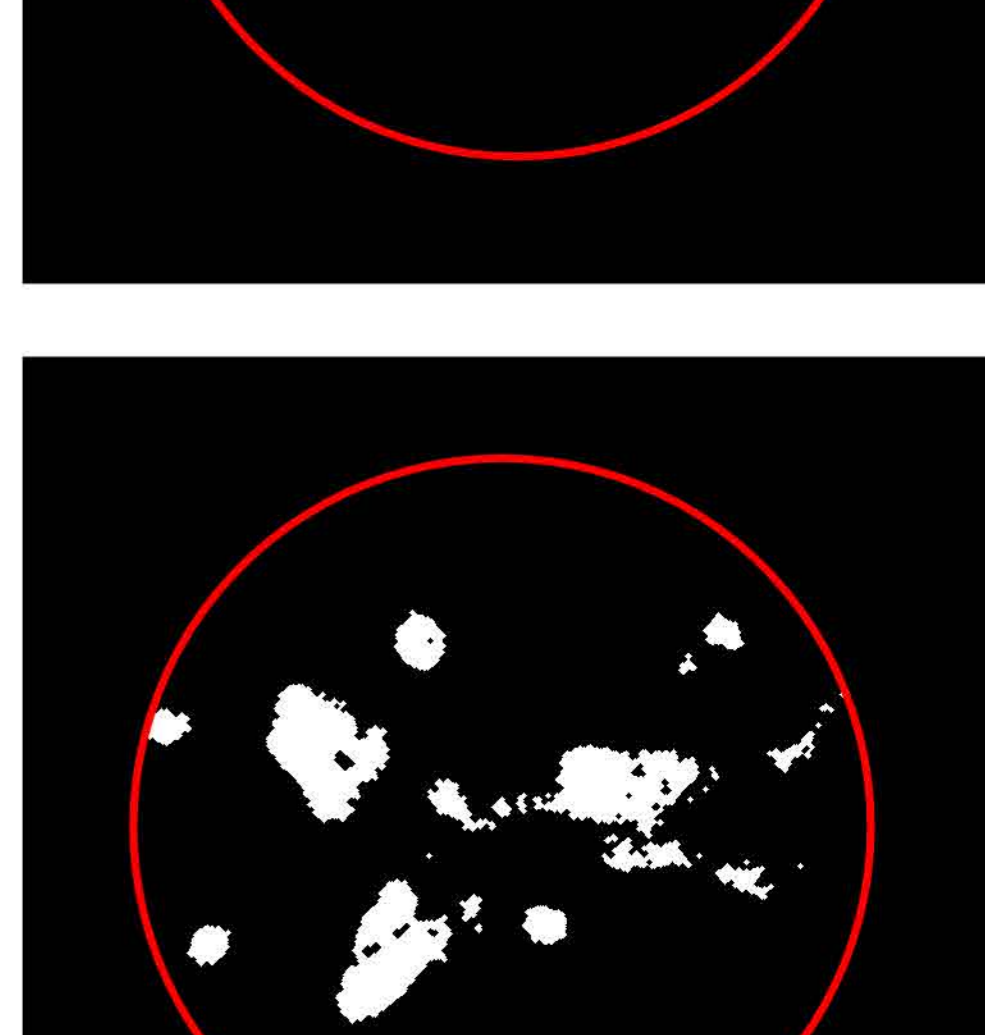 |
| 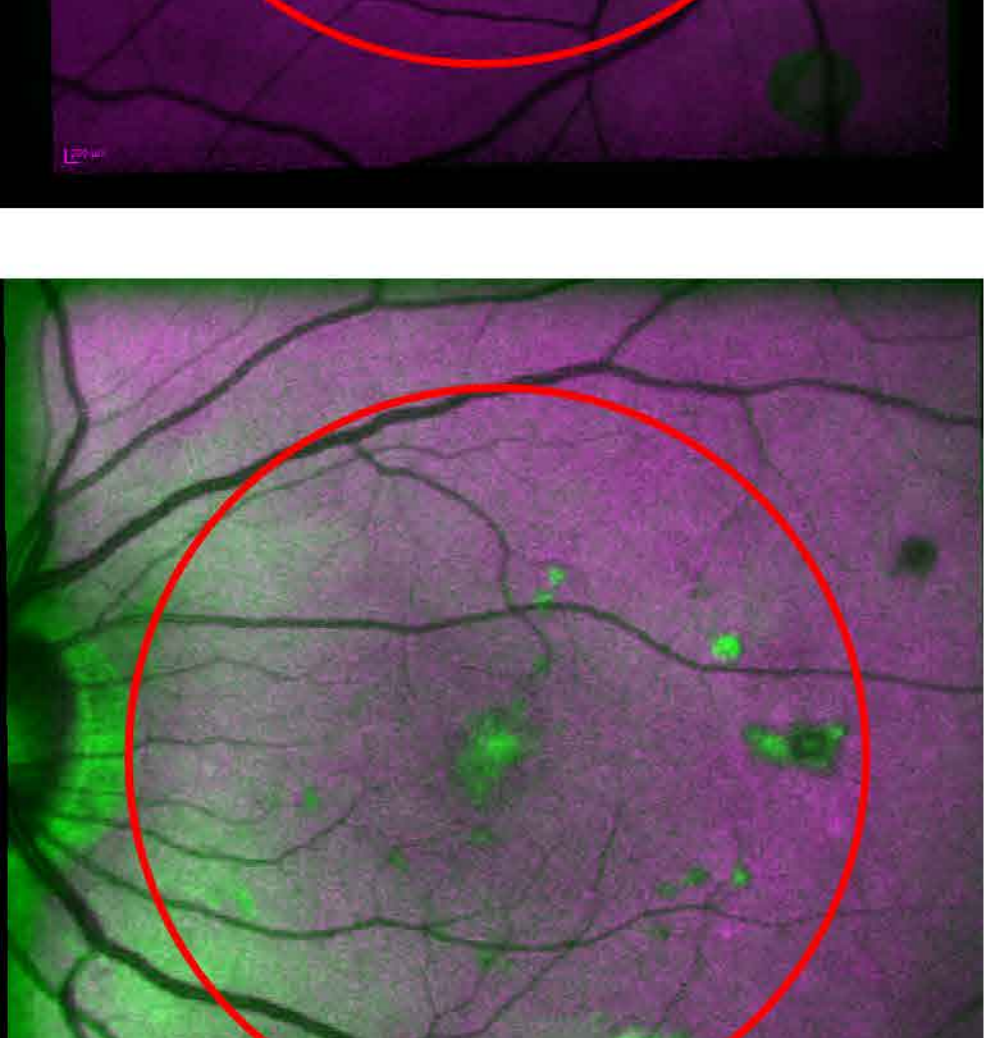 | 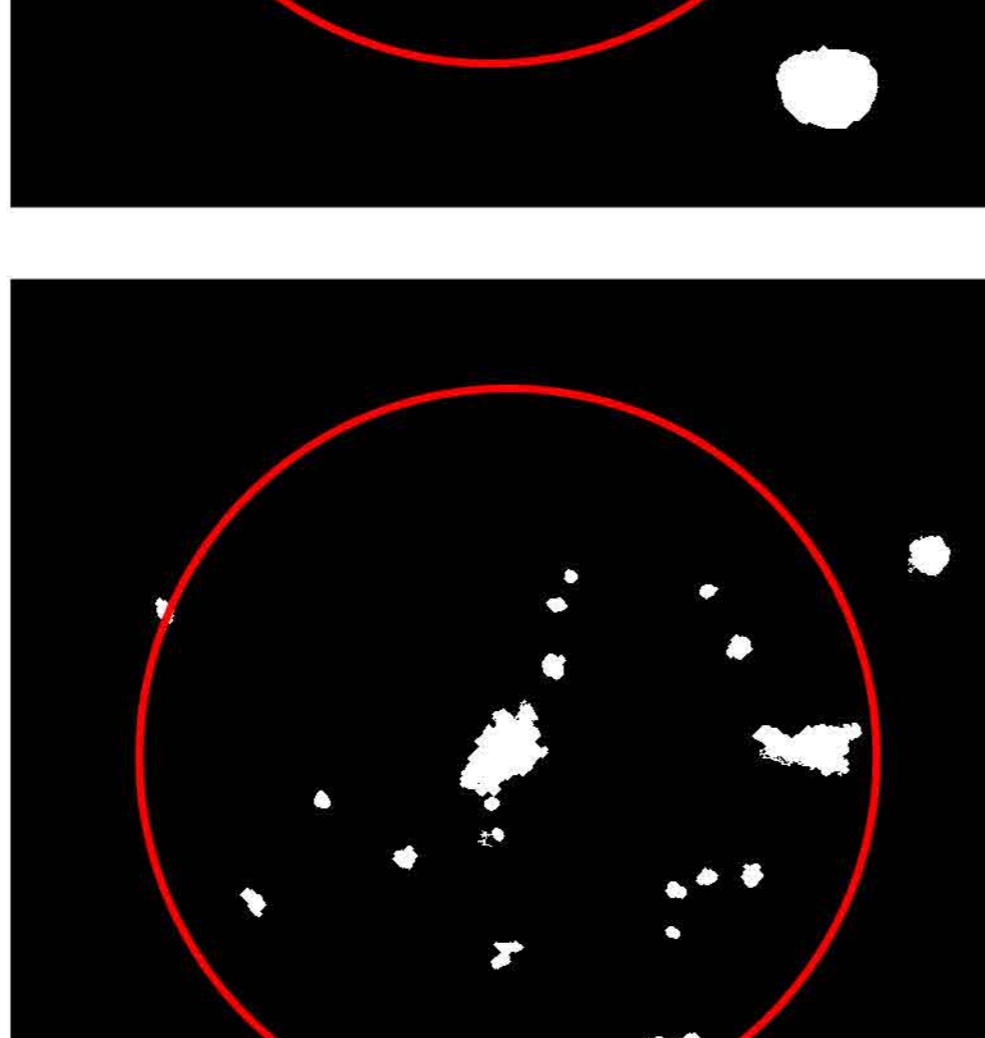 | 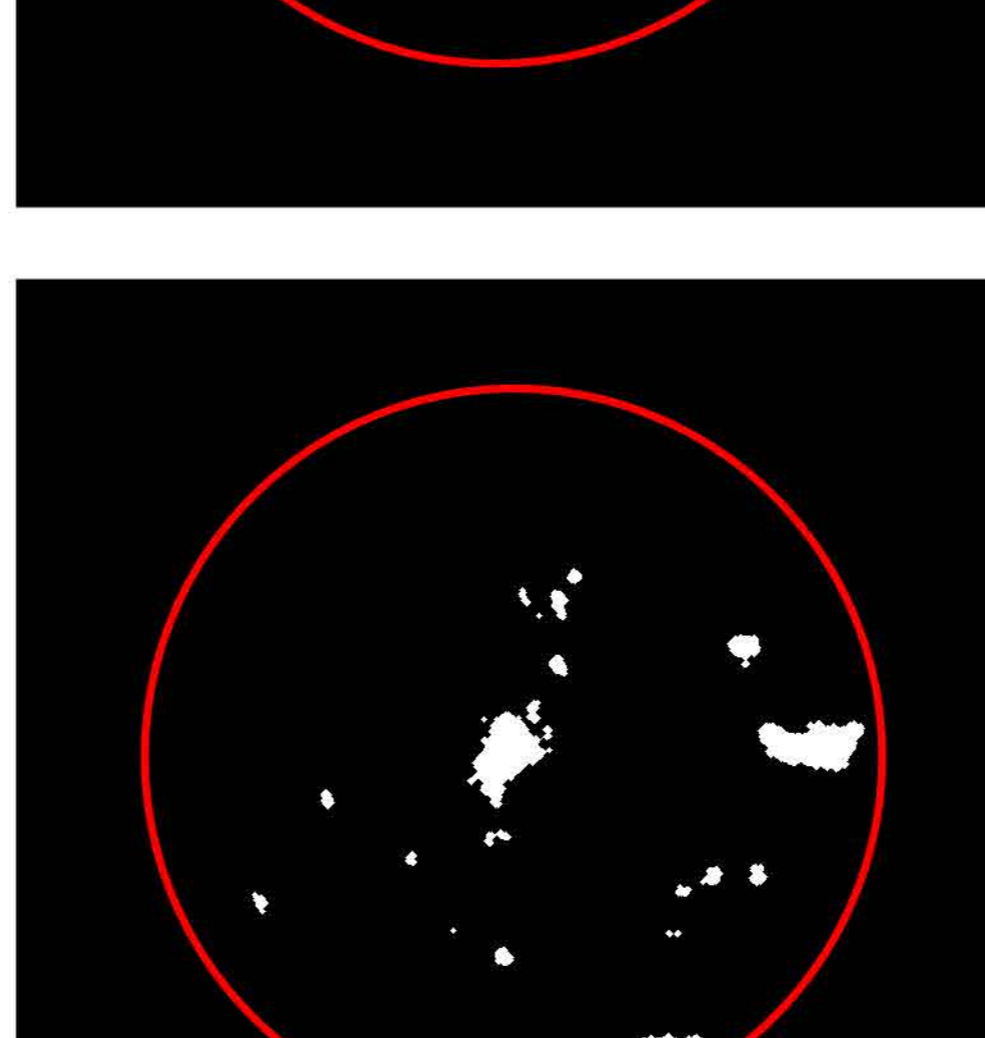 | 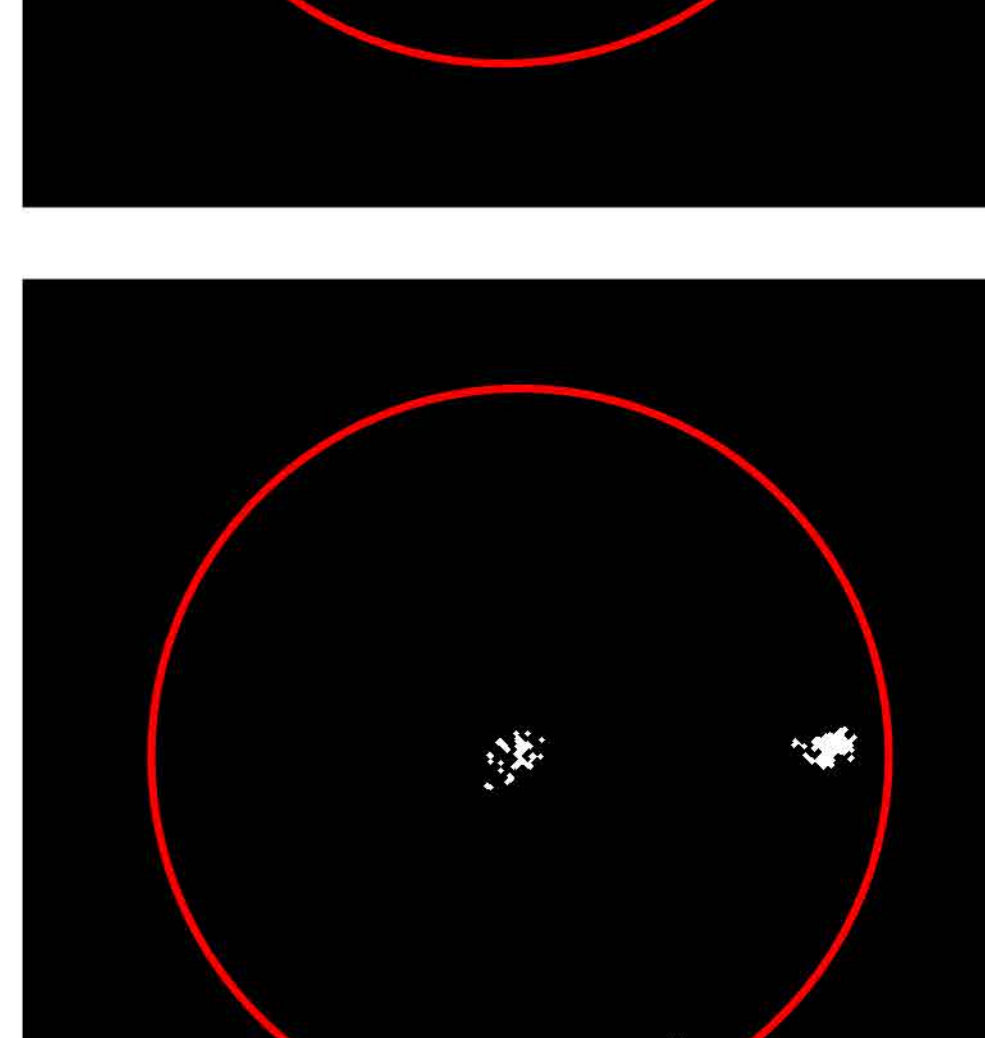 |
| 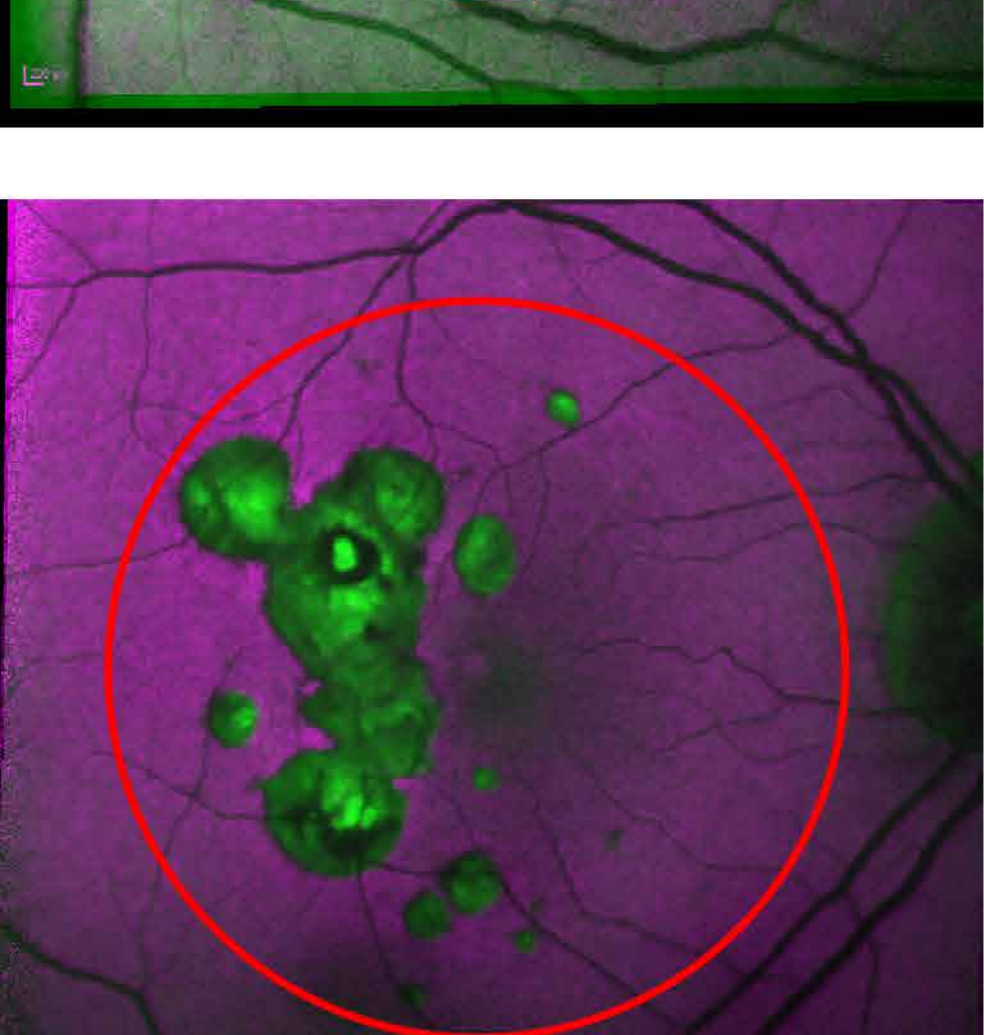 | 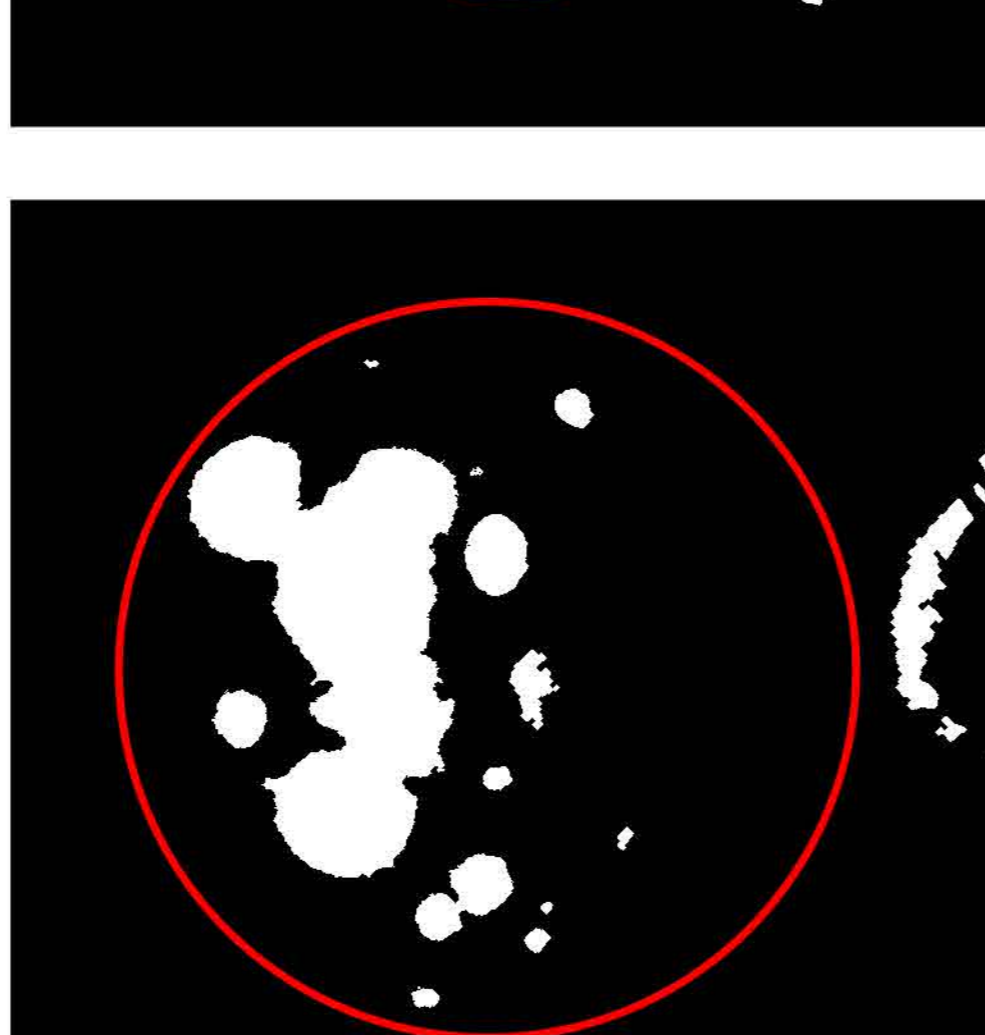 | 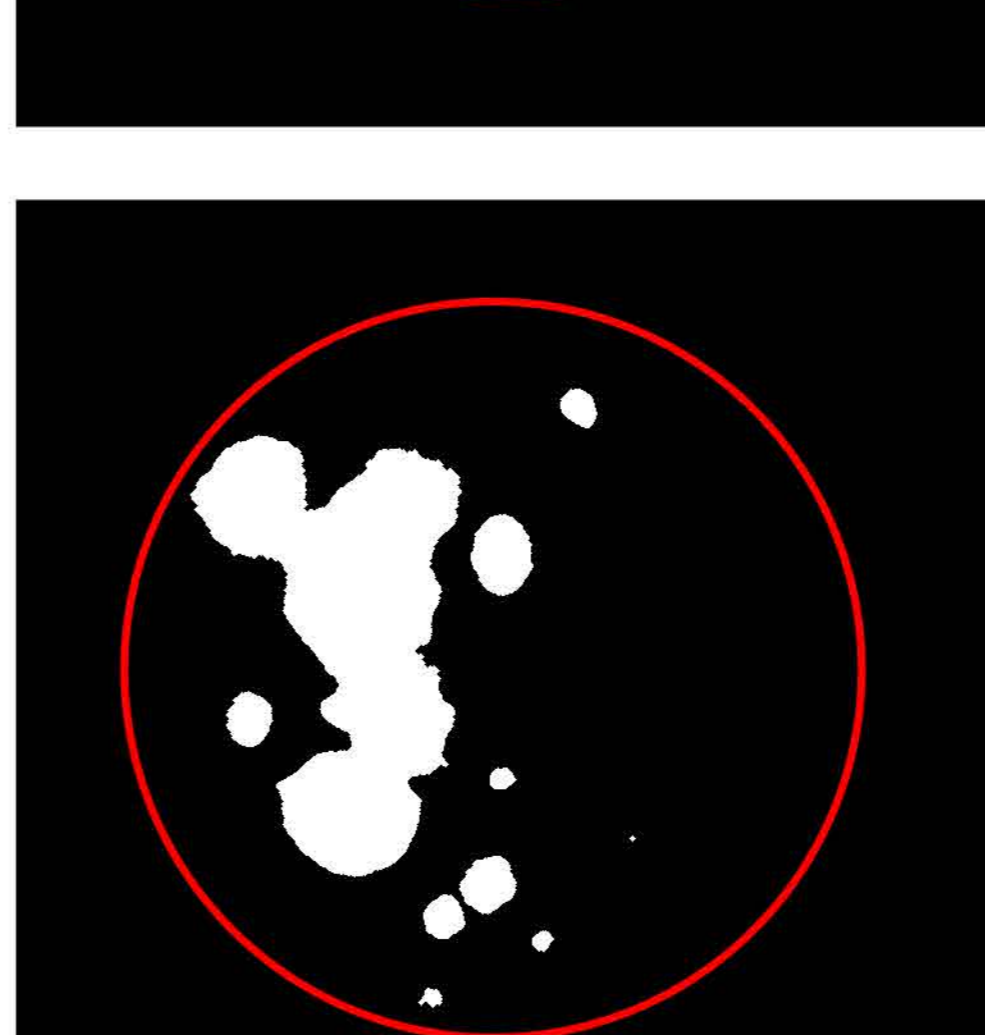 | 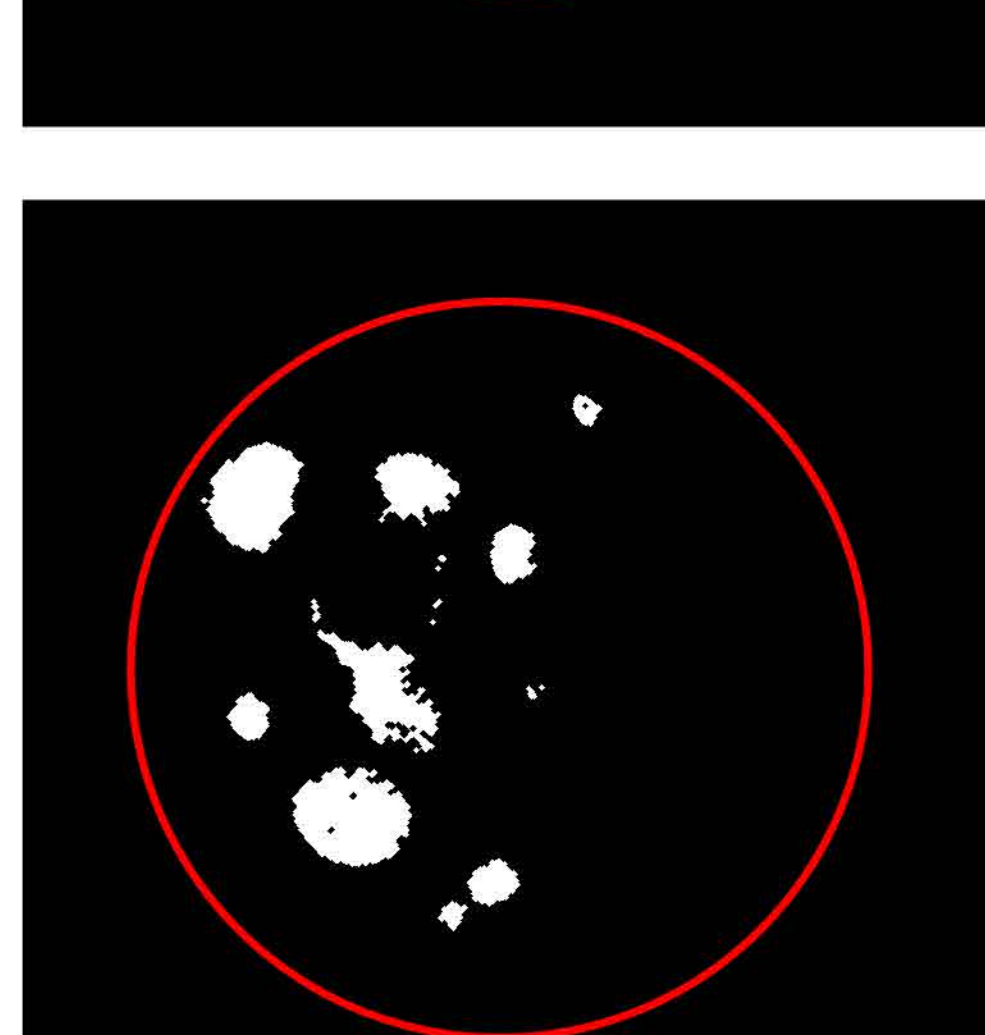 |
| 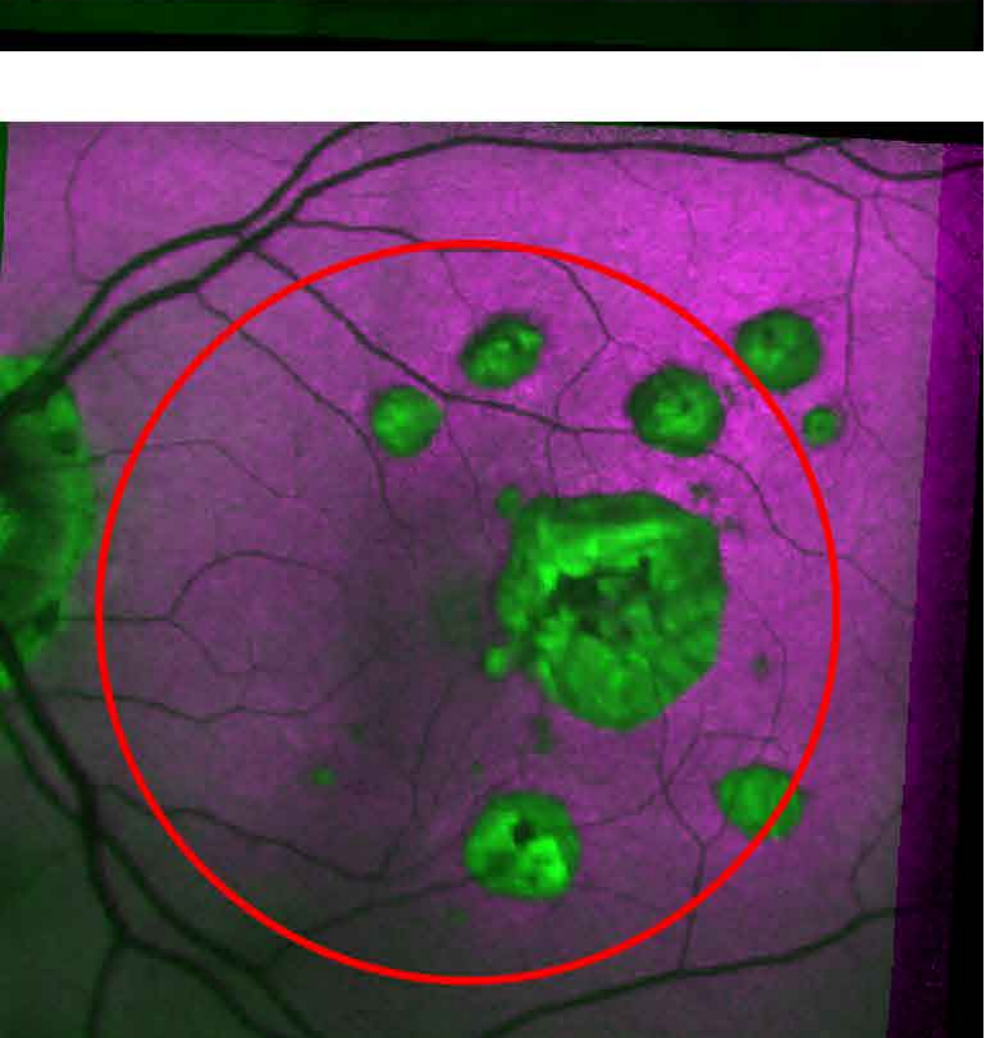 | 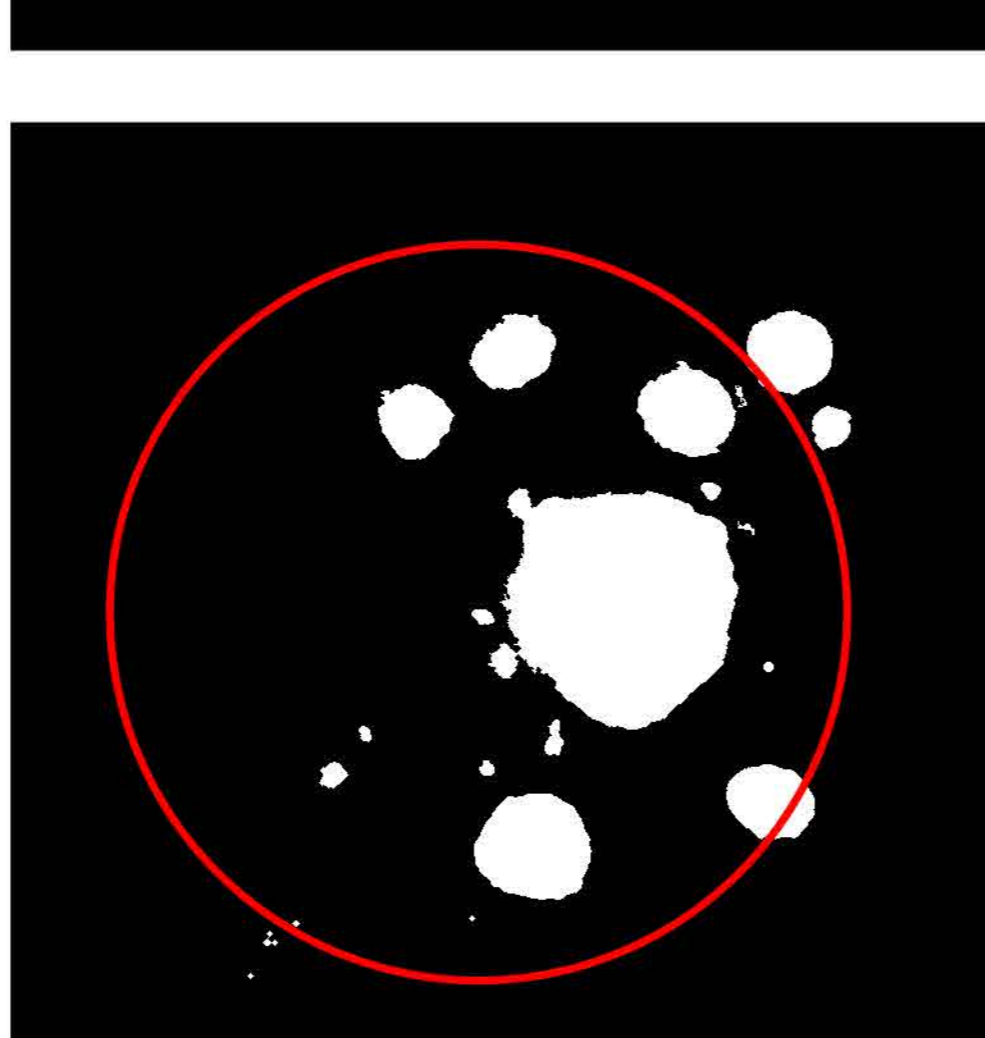 | 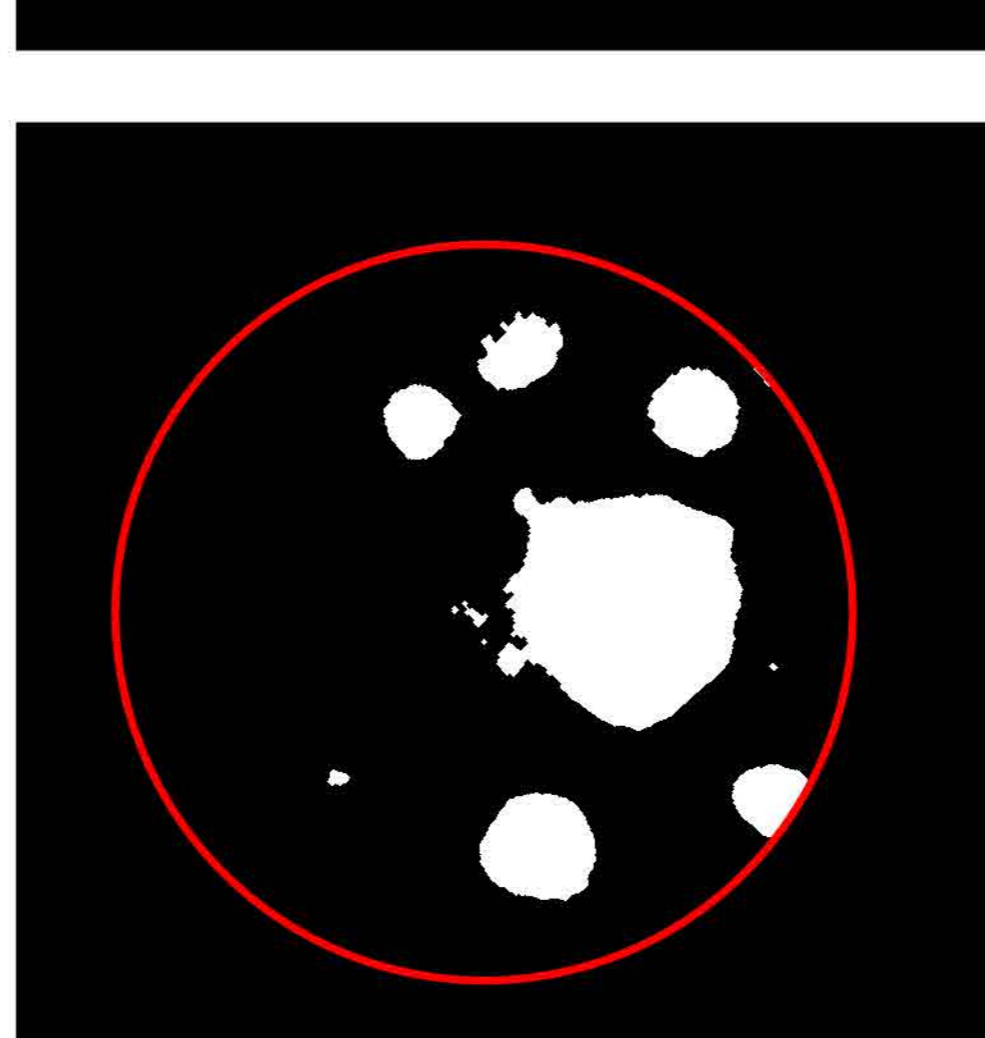 | 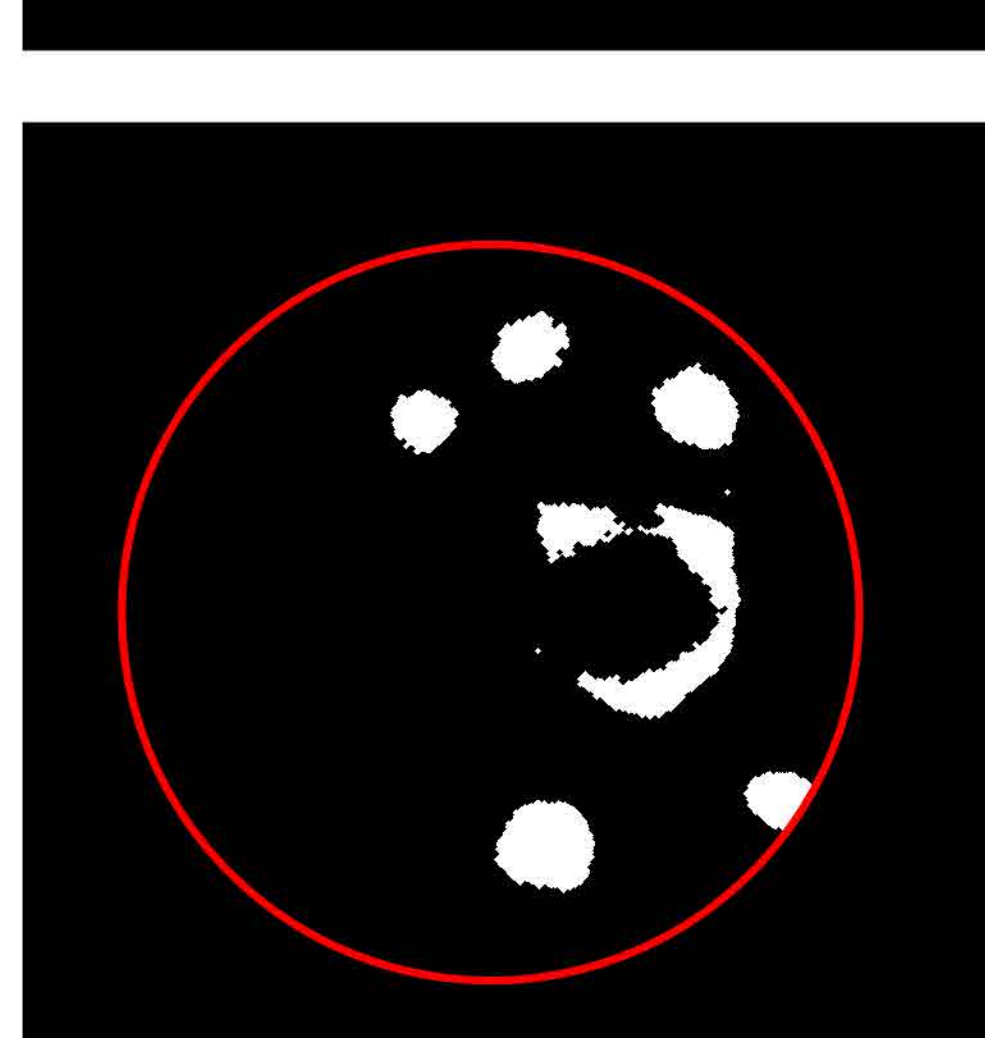 |
| 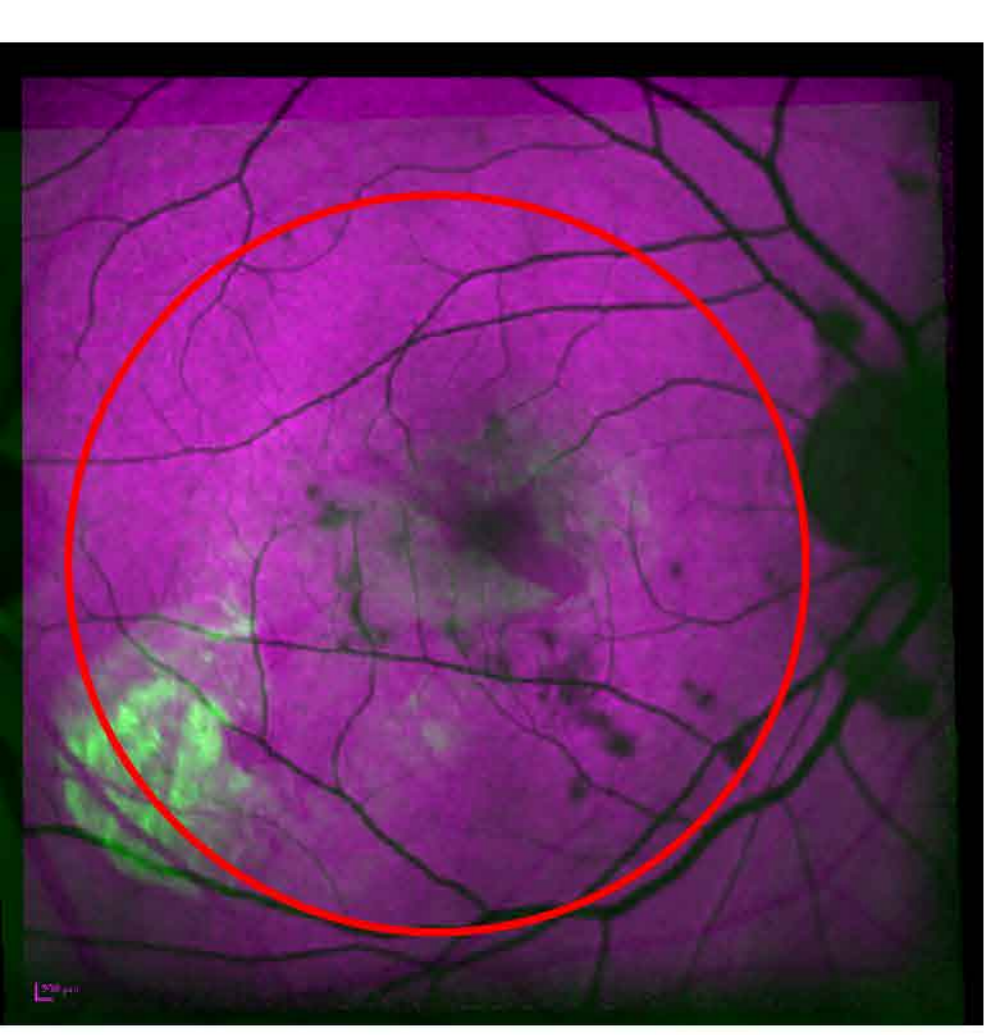 | 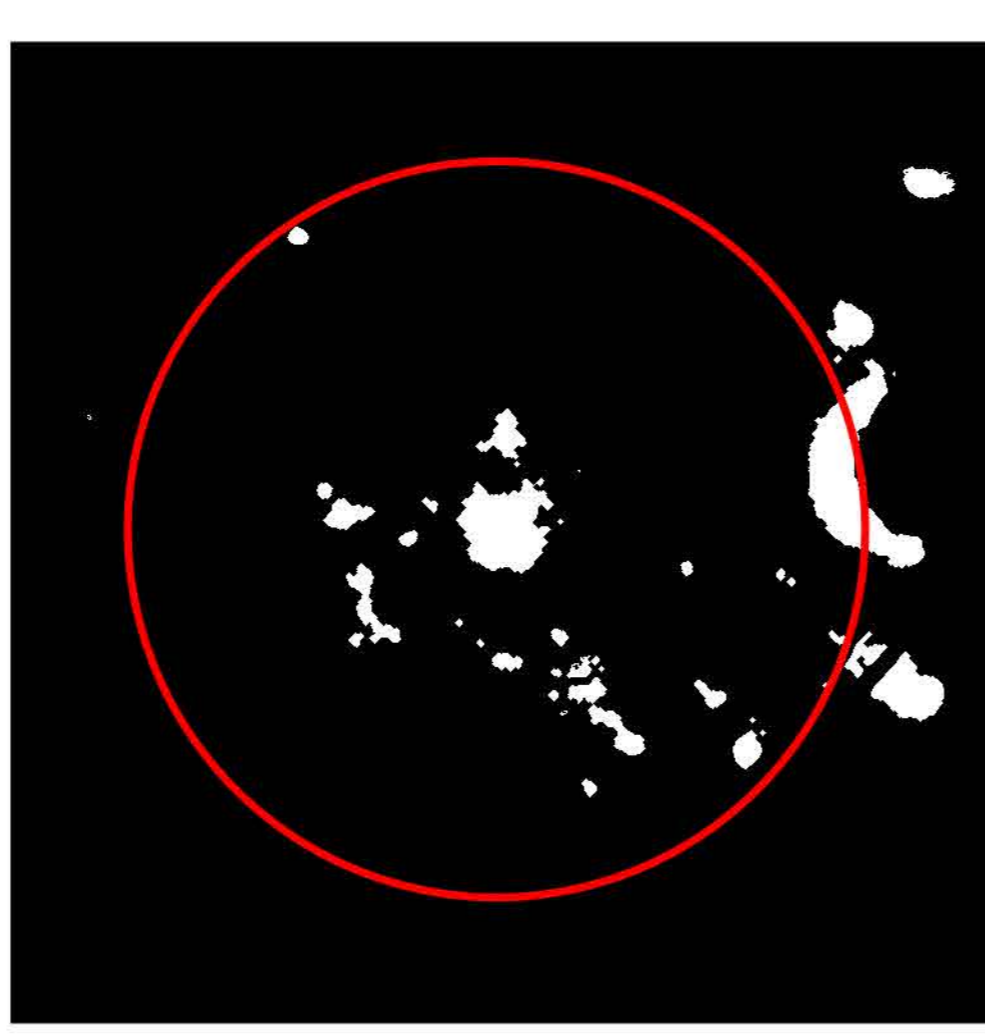 | 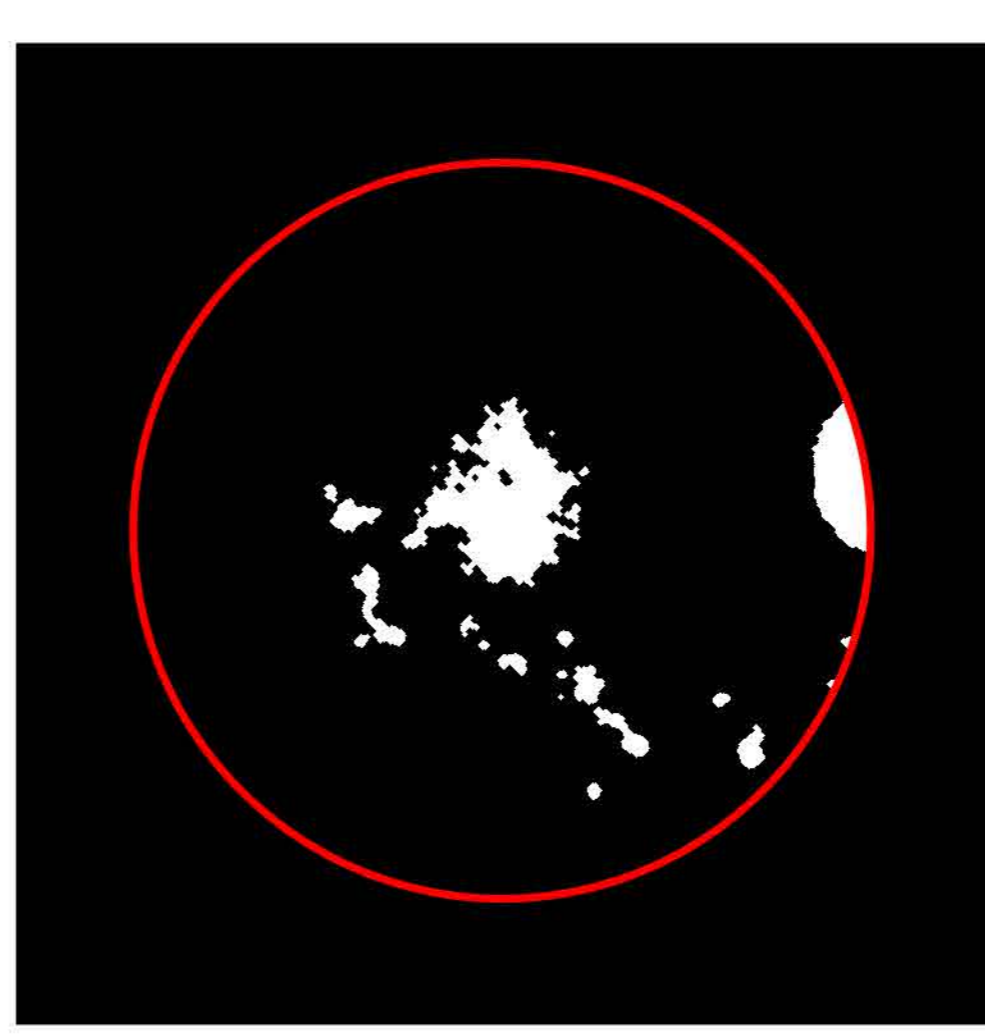 | 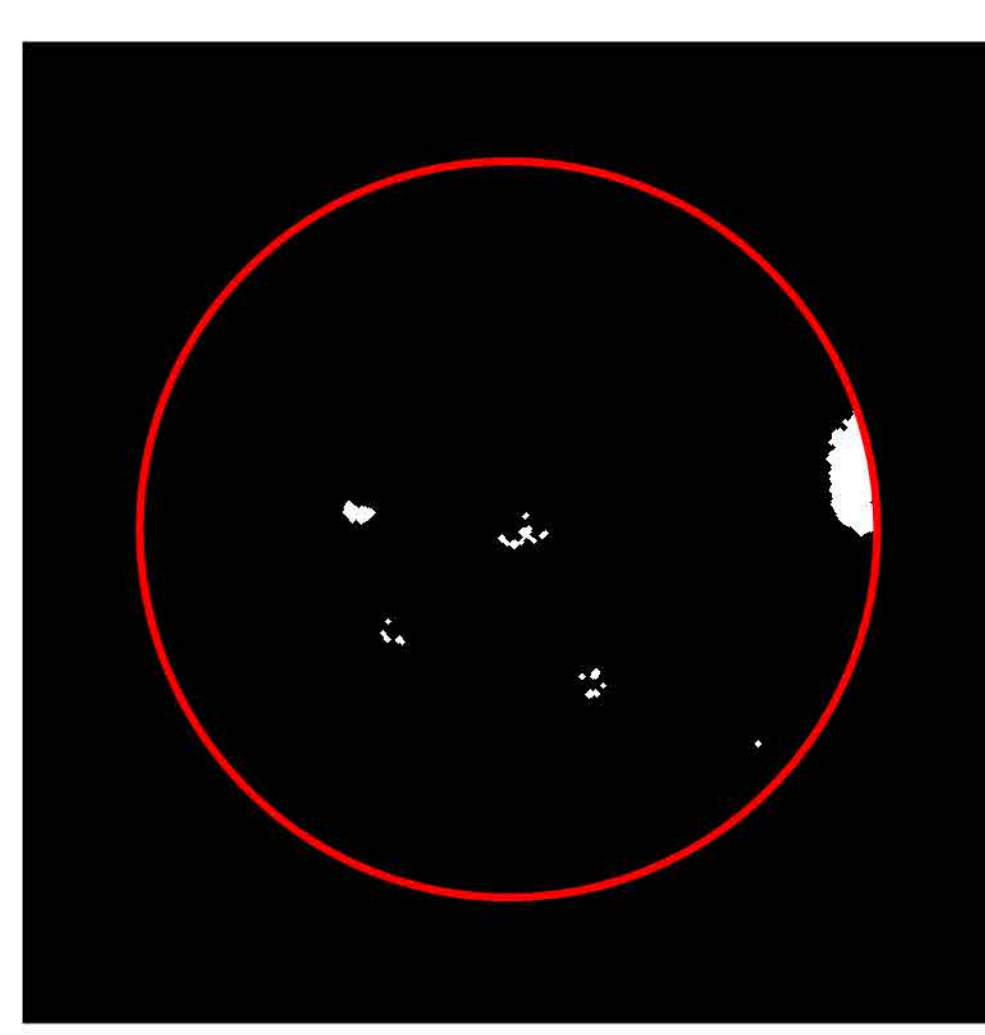 |
| 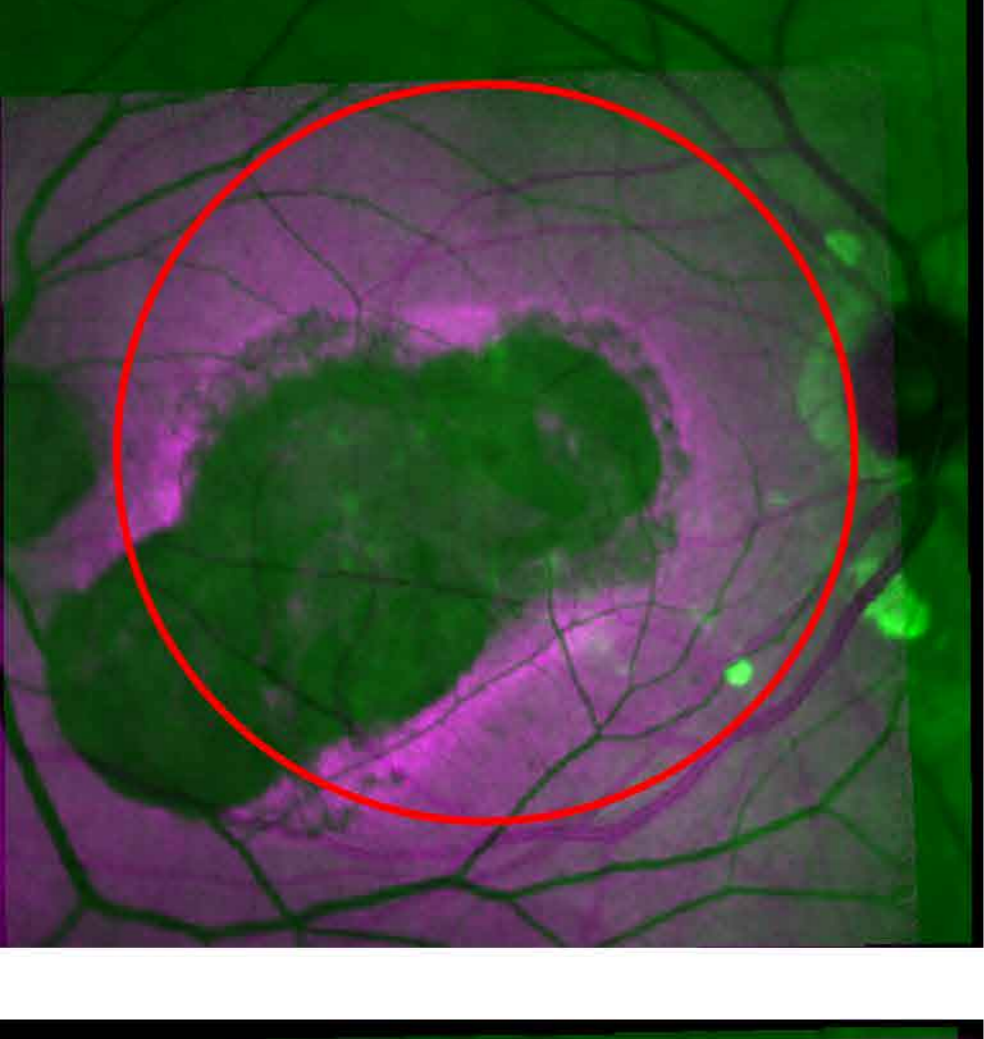 | 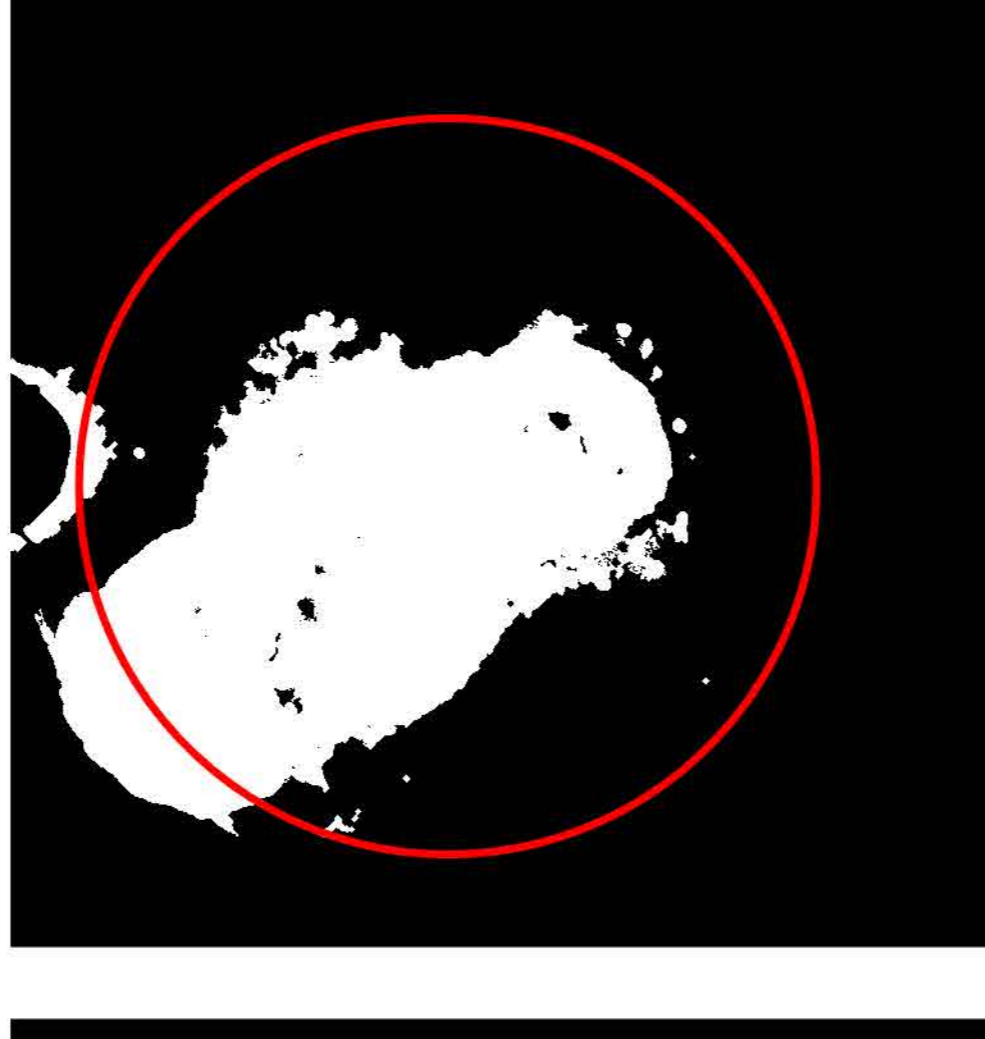 | 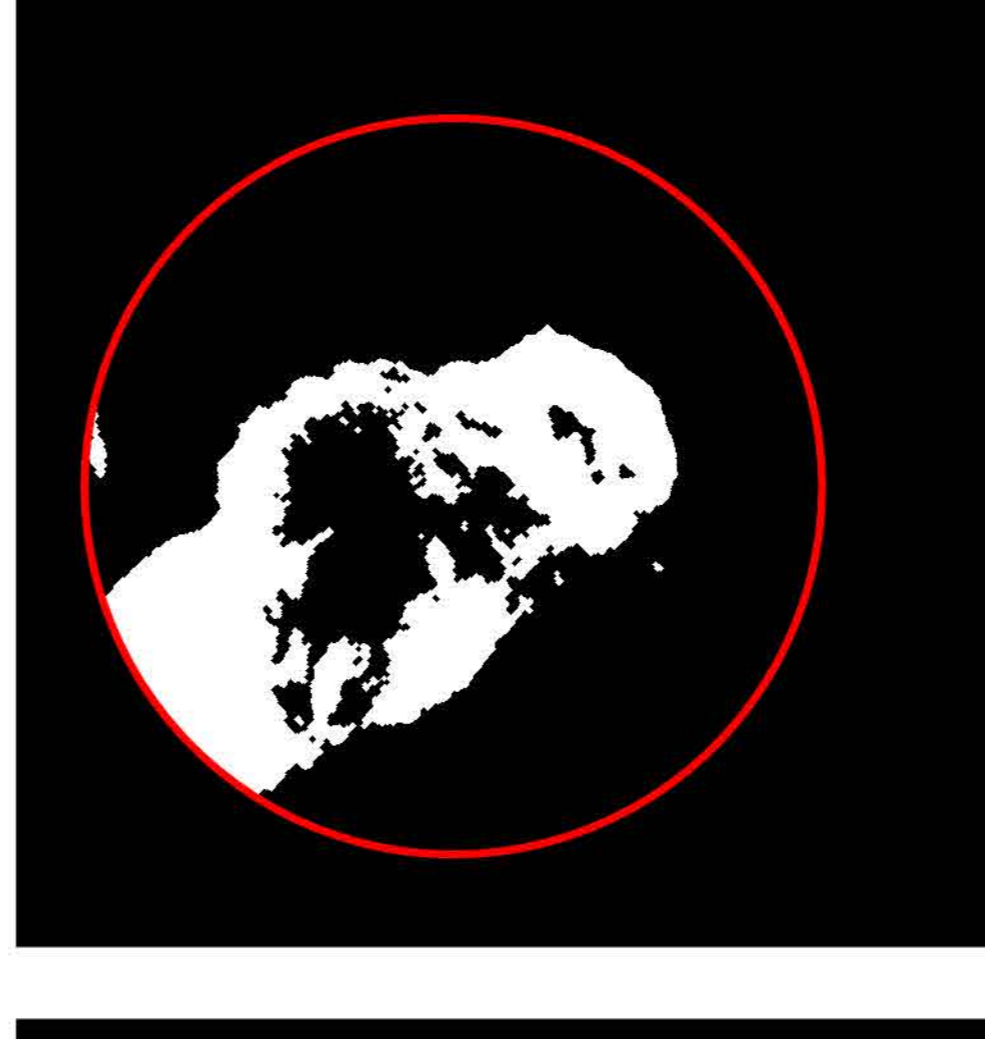 | 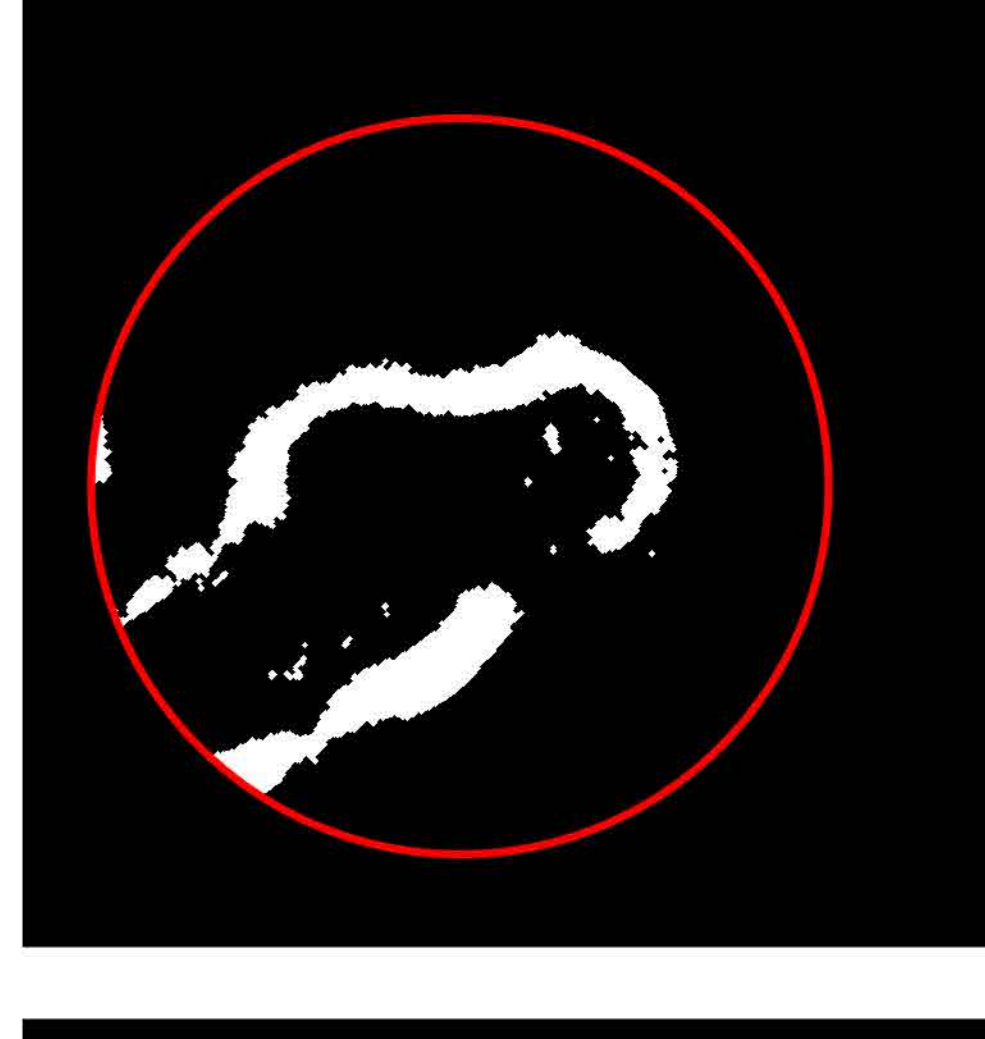 |
| 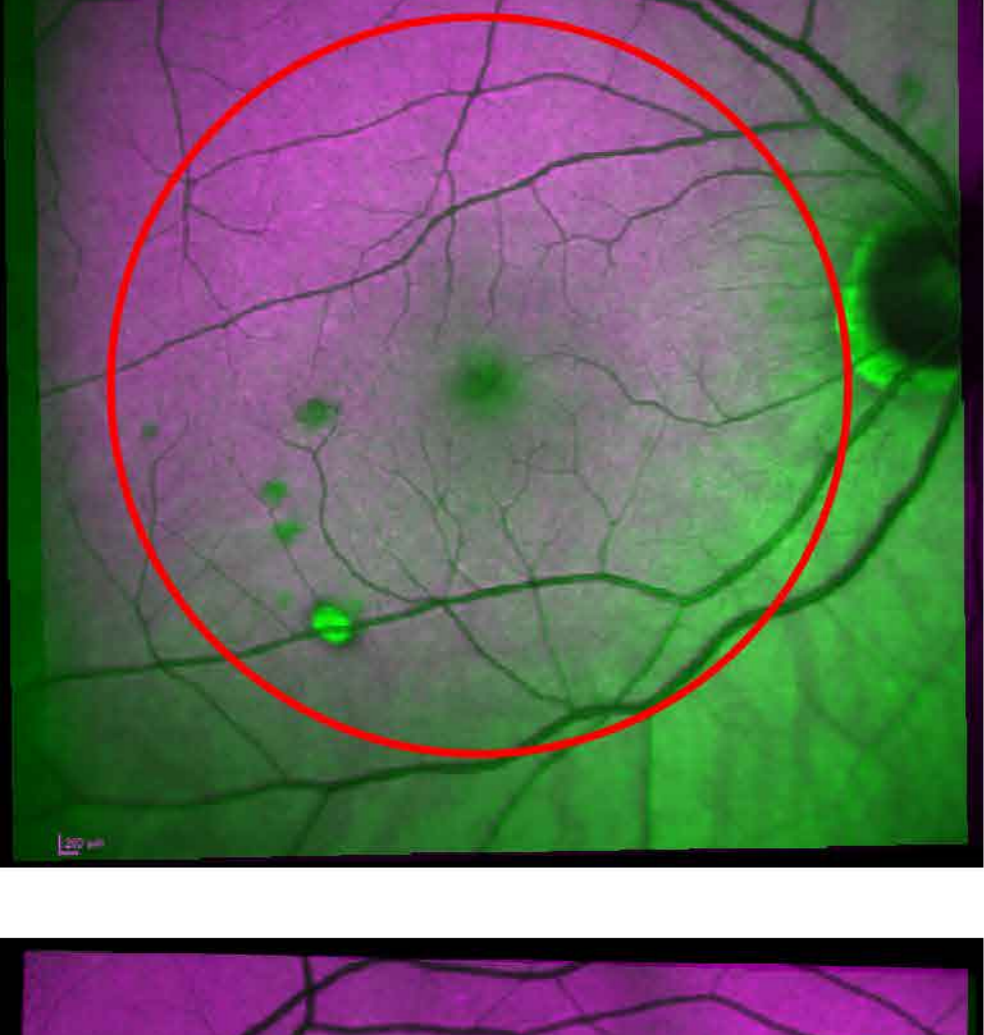 | 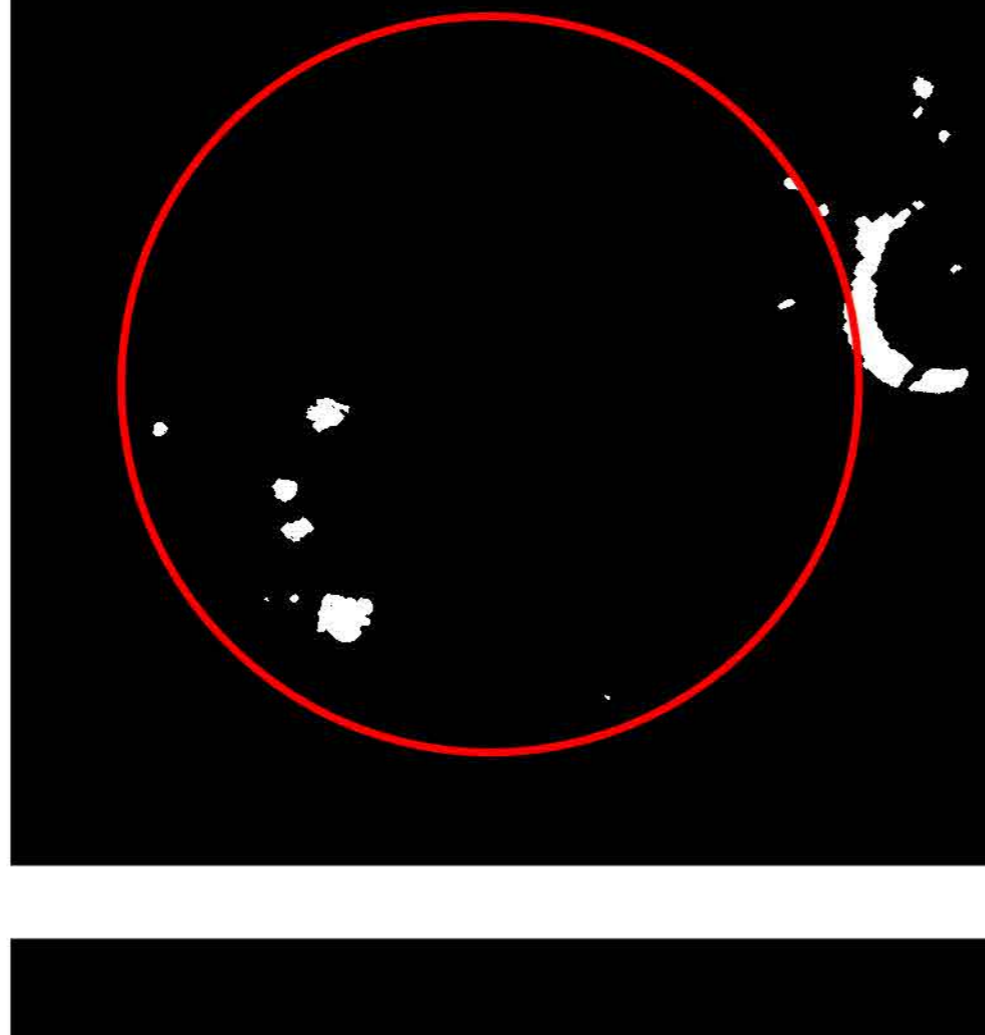 | 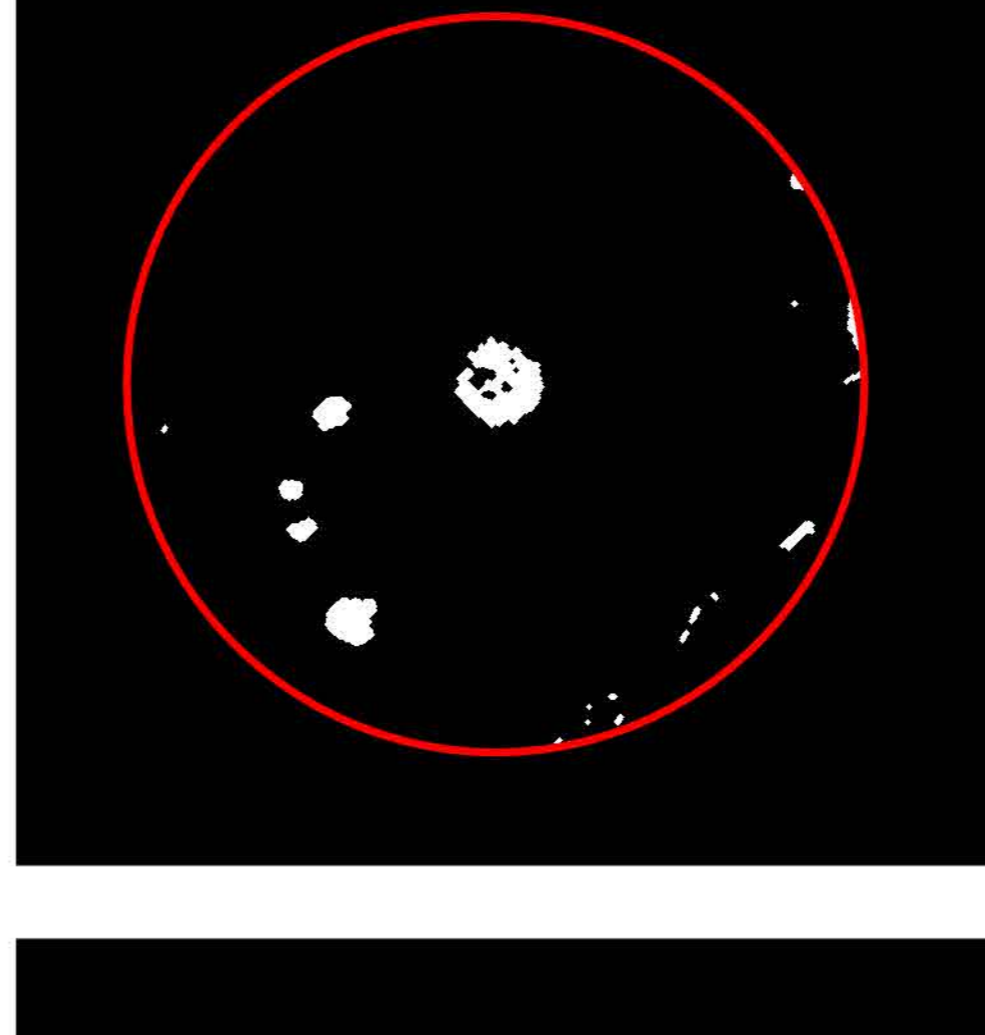 | 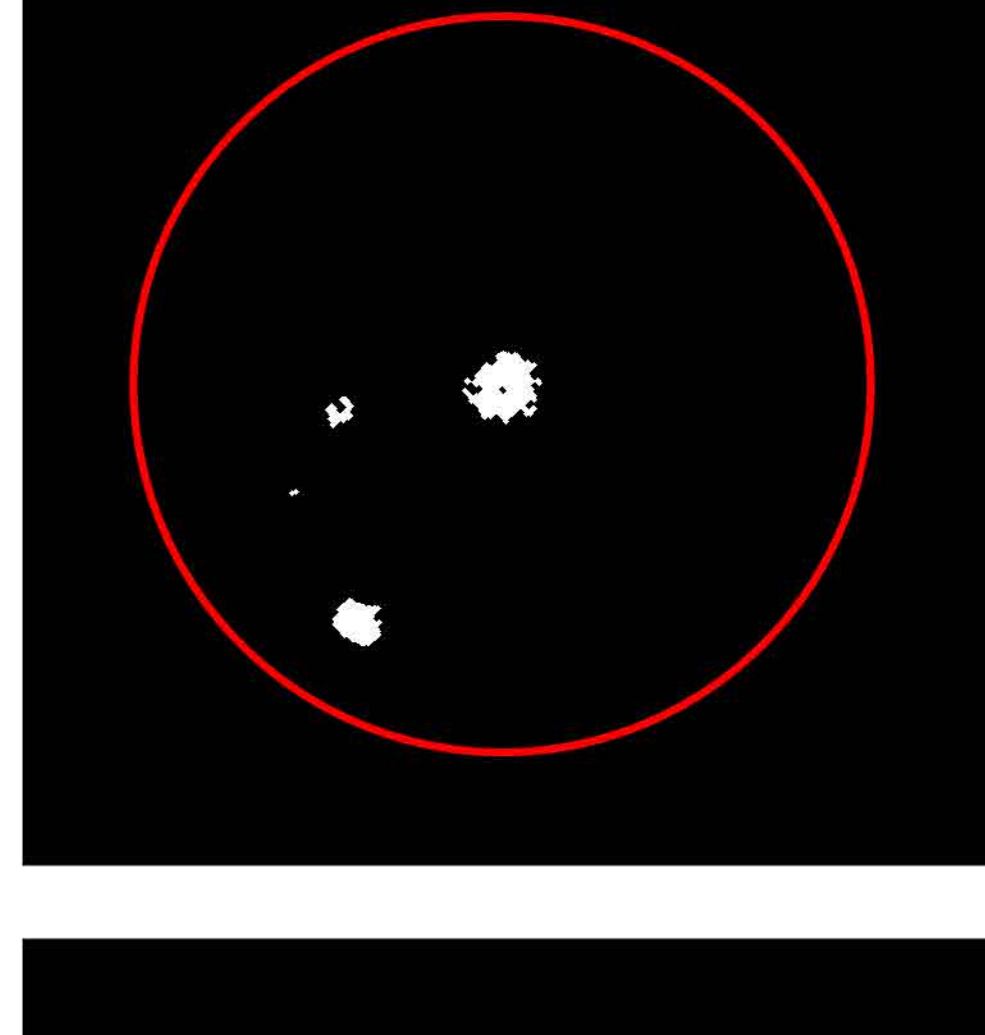 |
| 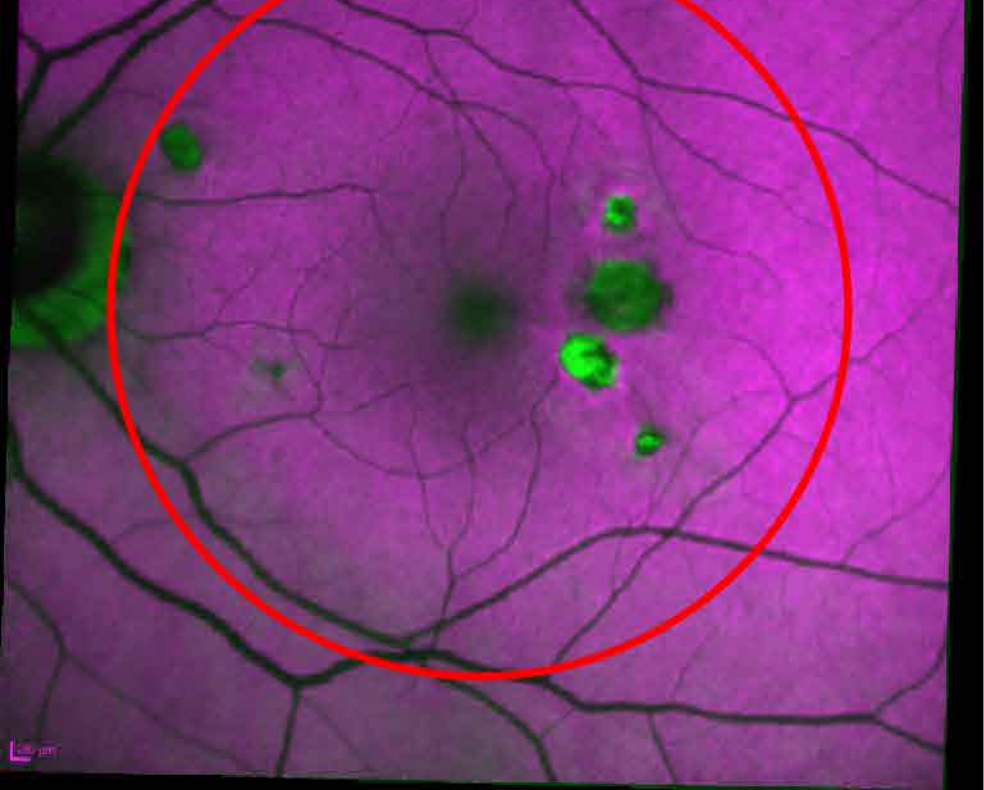 | 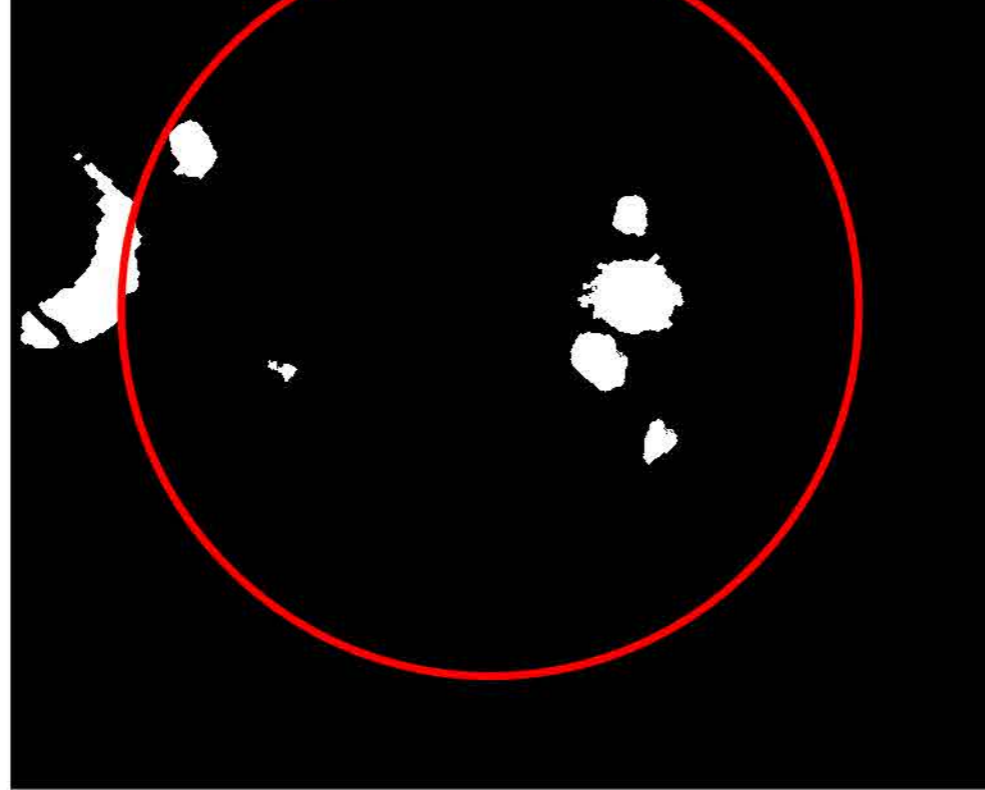 | 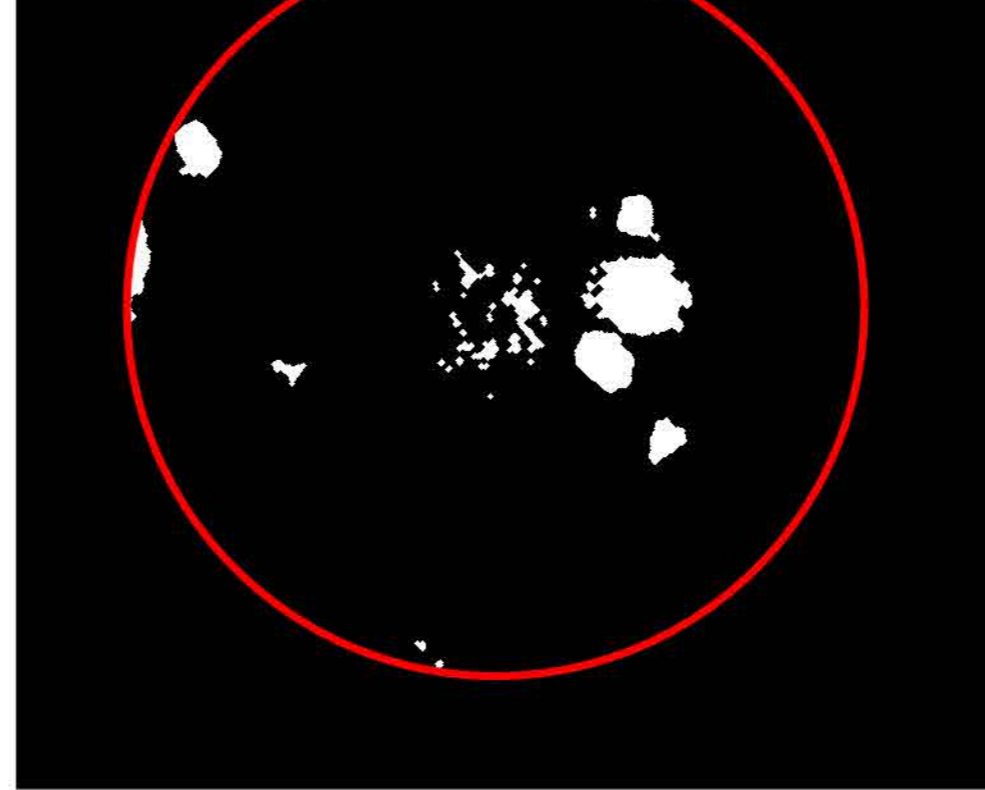 | 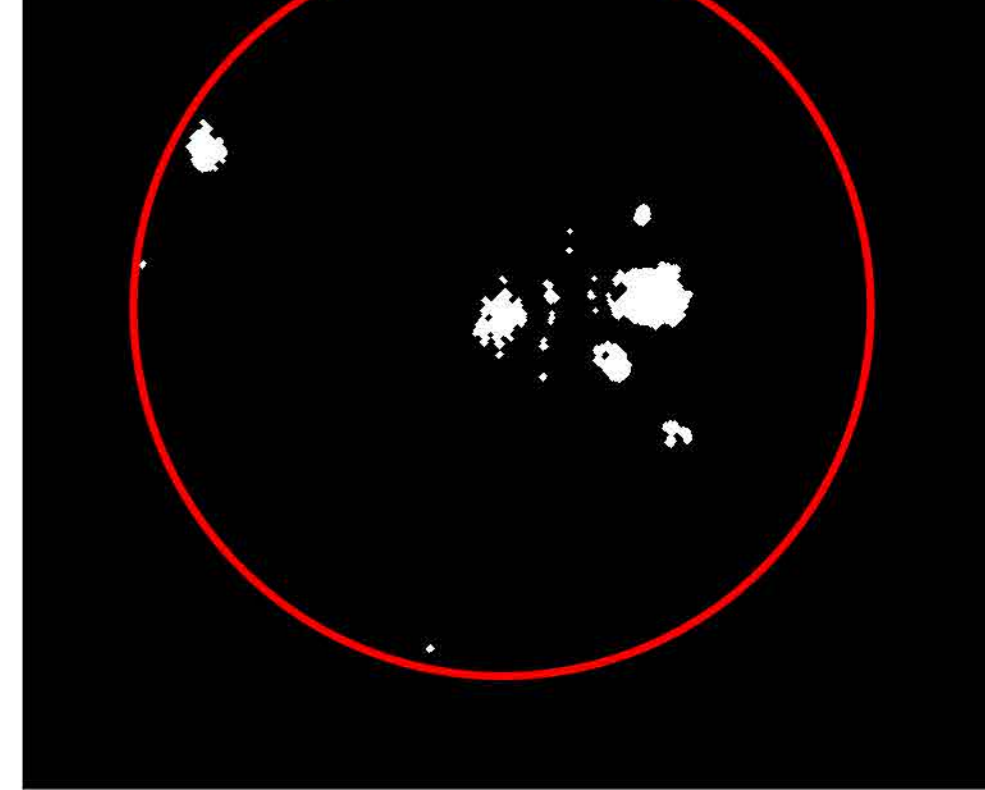 |
|  |  |  |  |
|  |  |  |  |
|  |  |  |  |

Supplement: Supplement 1 [file tvst-9-9-38_s001.pdf]
